# Protecting Athletes: The Clinical Relevance of Meta-Analyses on Injury Prevention Programs for Sports and Musculoskeletal Body Regions: An Overview of Systematic Reviews with Meta-Analyses of Randomized Clinical Trials

**DOI:** 10.3390/healthcare13131530

**Published:** 2025-06-27

**Authors:** Saúl Pineda-Escobar, Javier Matias-Soto, Cristina García-Muñoz, Javier Martinez-Calderon

**Affiliations:** 1 Department of Physiotherapy, Faculty of Nursing, Physiotherapy and Podiatry, University of Seville, 41009 Seville, Spain; saulpinedaescobar@gmail.com; 2 CTS 1110: Understanding Movement and Self in Health from Science (UMSS) Research Group, 41009 Andalusia, Spain; msjavi93@gmail.com (J.M.-S.); jmcalderon@us.es (J.M.-C.); 3 Instituto de Biomedicina de Sevilla-IBiS, Hospitales Universitarios Virgen del Rocío y Macarena, Universidad de Sevilla, 41013 Sevilla, Spain; 4 Departamento de Ciencias de la Salud y Biomédicas, Facultad Ciencias de la Salud, Universidad Loyola Andalucía, 41704 Sevilla, Spain

**Keywords:** athlete, incidence, meta-analysis, musculoskeletal, overview, review, sport

## Abstract

**Background**: Musculoskeletal injuries have a substantial impact on athletes, affecting sports performance and increasing the risk of future musculoskeletal disorders (e.g., osteoarthritis). Injury prevention programs are essential to reduce the risk of sport-related injuries and meta-analyses can provide a large amount of information in a single article. **Objective**: To summarize the pooled effects of injury prevention programs focused on any form of physical exercise in the incidence and risk of musculoskeletal injuries and reinjuries by sports and musculoskeletal body regions. **Methods**: The CINAHL (via EBSCOhost), Embase (via Elsevier), Epistemonikos, PubMed, Scopus, SPORTDiscus (via EBSCOhost), and the Cochrane Library e-databases were searched from inception to 7 October 2024. Systematic reviews with meta-analyses of randomized clinical trials were considered. The methodological quality of systematic reviews was assessed with AMSTAR 2. The degree of overlap between meta-analyses of interest was calculated. **Results**: Fourteen systematic reviews were included. Thirteen of these reviews were focused on soccer. Overall, meta-analyses including a specific injury prevention program (FIFA 11+ and FIFA 11+ kids) found that these programs may reduce the risk of musculoskeletal injuries among soccer players. Concretely, FIFA 11+ may reduce the risk of ankle, knee, hip/groin, and hamstring injuries, whereas FIFA 11+ kids may decrease the risk of ankle and knee injuries. **Conclusions**: FIFA 11+ and FIFA 11+ kids may reduce the risk of sports musculoskeletal injuries, mainly in the lower limbs. However, many clinical and methodological issues (e.g., the lack of meta-analyses in many types of sports) were discussed and highlighted the difficulty of making robust clinical recommendations with the current data.

## 1. Introduction

Sport-related musculoskeletal injuries are mainly characterized by contact or non-contact injuries that occur in either practices or competitions and affect muscles, bones, tendons, joints, ligaments, and other soft tissues [1]. When musculoskeletal injuries appear they have a substantial impact on athletes, altering sports performance [2] and fostering future musculoskeletal disorders (e.g., osteoarthritis), as well as mental health issues such as distress, anxiety, and sleep disturbances [3,4]. Musculoskeletal injuries have also important implications in sports competitions, delaying the return to play [5], increasing practice and game time loss [6], and raising the economic burden on athletes, teams, and sporting competitions [7,8].

Sport-related musculoskeletal injuries are highly incident in many sports disciplines and the epidemiology, causes, and factors related to the appearance of injuries can vary greatly between sports, levels of play, sex, and age groups. For example, anterior cruciate ligament injuries may be more incident in female basketball players, whereas male basketball players may have a higher incidence of ankle sprain [9]. Hamstring injuries may represent around 10% of all injuries in field-based team sports such as soccer, rugby union, field hockey, Gaelic football, hurling, and Australian football [10]. Furthermore, shoulder strains and tendinopathies may be very common among softball players [11], and shoulder injuries may have a high probability of becoming chronic in sports such as water polo [12].

In this context, injury prevention programs (IPPs) may be essential to reduce the incidence of sport-related musculoskeletal injuries and the risk of sport-related musculoskeletal reinjuries. In sports, IPPs are considered cost-effective, with total cost savings that may reach up to €462 per athlete [13]. Furthermore, IPPs focused on physical exercise have received great interest, and physical exercise is one of the first-line treatments for musculoskeletal disorders [14,15]. Programs such as FIFA 11, FIFA 11 +, FIFA 11 + kids, balance board training, the prevention injury and enhance performance program, the bounding exercise program, and neuromuscular training (Knäkontroll) program are mainly based on different types of physical exercises, and some systematic reviews have underlined their positive effects on hamstring injuries, knee injuries, or ankle injuries in sports disciplines such as soccer [16,17,18].

Recent advances have been made to increase the implementation of IPPs in sports, and an overview of reviews including more than 100 syntheses of the literature has highlighted the importance of establishing IPPs for helping athletes of different sports [19,20]. The proliferation in the number of systematic reviews in this field is a fact, but many of these systematic reviews have not developed meta-analyses [21,22], or have not performed specific meta-analyses by sports, as well as by body regions [23,24]. To our knowledge, there is no overview of systematic reviews with meta-analyses that has focused on including only those meta-analyses showing results that may have direct clinical applicability. Therefore, a new overview of systematic reviews with meta-analyses on this topic is timely and may help sports clinicians to consume a large amount of critically appraised information in a single article [25,26].

The objective of this overview of systematic reviews with meta-analyses was to summarize the pooled effects of IPPs focused on any form of physical exercise (e.g., aerobic training, regular physical activity, or yoga) in the incidence and risk of musculoskeletal injuries and reinjuries by sports and musculoskeletal body regions (e.g., IPPs on ankle injuries in soccer).

## 2. Materials and Methods

This overview of systematic reviews with meta-analyses followed the PRIOR statement [27] and the PRISMA statement for abstracts [28]. The review protocol was prospectively registered at Open Science Framework: https://doi.org/10.17605/OSF.IO/QB6JA.

### 2.1. Deviations from the Protocol

Deviations in the protocol were reported in Supplementary File S1.

### 2.2. Data Sources and Search Strategy

One co-author (JMC) screened the following e-databases: CINAHL (via EBSCOhost), Embase (via Elsevier), Epistemonikos, PubMed, Scopus, SPORTDiscus (via EBSCOhost), and the Cochrane Library from inception to 7 October 2024. Search filters by type of document (conference abstracts were not considered) were imposed when possible. The full search strategy was reported in Supplementary File S2. A manual search was developed to supplement search strategies. We manually screened syntheses of literature related to our scope that were retrieved during search strategies (e.g., scoping reviews or overviews of reviews).

### 2.3. Eligibility Criteria

The PICOS (Population, Intervention, Control, Outcome, Study Design) framework was used to develop the eligibility criteria [29].

Inclusion criteria:

P: Athletes without clinical (e.g., duration of musculoskeletal injury), personal (e.g., age groups), and sports (e.g., level of play) restrictions.

I: IPPs focused exclusively on physical exercise, regular physical activity, and/or mind–body exercise interventions (e.g., Nordic hamstring exercises). We also included those IPPs where any of these interventions were a core part, although other interventions were considered.

C: No restrictions were imposed.

O: Pooled incidence or risk of developing sport-related musculoskeletal injuries or reinjuries. We considered both non-contact and contact injuries. Musculoskeletal injuries are defined as those injuries that occur in either practices or matches and affect muscles, bones, tendons, joints, ligaments, and other soft tissues [1]. We focused these outcomes on meta-analyses conducted by sports and musculoskeletal body regions (e.g., IPPs on the incidence of ankle injuries in basketball). If possible, we also considered any subgroup meta-analyses developed by sports and musculoskeletal body regions, but adding other important factors (e.g., sex-differences IPPs ankle injuries in basketball). In terms of age groups, the results were divided into children, adolescents, and adults, when possible. We followed the suggestions of the World Health Organization to consider children and adolescents those participants 19 years old or younger (https://www.who.int/health-topics/adolescent-health/#tab=tab_1, accessed on 10 September 2024).

S: Systematic reviews with meta-analyses of randomized clinical trials (e.g., cluster randomized controlled trials) published in peer-reviewed journals. We included all studies that were justified as systematic reviews. Meta-analyses evaluating two or more original studies were only considered.

Exclusion criteria:

[I] Systematic reviews did not specify in their methods that meta-analyses or their subgroups were performed by sports and musculoskeletal body regions and included only randomized clinical trials.

[II] Meta-analyses combined different sports injuries rather than musculoskeletal injuries (e.g., concussion).

[III] Meta-analyses include studies evaluating army recruits. Although army recruits are sometimes considered to be athletes, we have excluded them since the training environment and objectives of this population are completely different from other athletes (e.g., basketball players).

[IV] Conference abstracts and proceedings.

[V] No full-text access. This criterion was applied if, after requesting the full text from the authors, we did not receive a response, or they did not send us the full text.

[VI] In network meta-analyses, no direct comparisons were reported.

### 2.4. Study Selection

Study selection was independently conducted by two co-authors (JMC and SPE). One co-author (JMC) used Zotero 6.0.36 Citation Management Software to include the references retrieved by e-databases. All references were manually checked, and duplicates were removed. Then, titles and abstracts were read and irrelevant studies regarding the objectives of this overview were excluded. Subsequently, JMC and SPE independently analyzed full texts if abstracts seemed eligible or if abstracts were unavailable. Disagreements between these co-authors were solved by consensus. We consulted with a third co-author (CGM) for the inclusion of two studies [30,31]. The percentage of agreement between JMC and SPE was calculated using the number of studies rated with the same score before pooling the results of their independent assessments. The percentage of agreement was 98.7%.

### 2.5. Methodological Quality Assessment of Systematic Reviews

Two co-authors (SPE and JMS) independently used AMSTAR 2 to analyze the methodological quality of systematic reviews [32]. This tool is composed of sixteen items that can be evaluated as yes, partially yes, or no. Using the overall score is not recommended [32], but the following items are considered critical: items: 2, 4, 7, 9, 11, 13, 15 [32]. We solved disagreements between SPE and JMS by consensus and we calculated the percentage of agreement between them using the number of items rated with the same score before pooling the results of their independent assessments.

### 2.6. The Degree of Overlap Between Reviews

One co-author (JMC) built matrices of evidence to calculate the corrected covered area (CCA) that is needed to know the degree of overlap between systematic reviews [33]. The CCA is defined as the area that is covered once original studies are removed the first time they are counted. The degree of overlap can be classified as slight (CCA 0–5%), moderate (CCA 6–10%), high (CCA 11–15%), or very high (CCA > 15%) [33].

We only calculated the degree of overlap when at least two systematic reviews meta-analyzed the same sport and musculoskeletal body region (e.g., ankle injuries in soccer). As we only included in this overview those meta-analyses that satisfied our inclusion criteria, we only considered the references included in the meta-analyses of interest to calculate the degree of overlap. Finally, one co-author (CGM) built a bar plot to depict the degree of overlap between systematic reviews.

### 2.7. Data Extraction

Two co-authors (JMC and SPE) extracted independently from each review the following information when possible: (1) total sample size, (2) total hours of exposure, (3) the number and type of injury, (4) mechanism of injury (non-contact or contact), (5) type of sport, (6) level of play, (7) age groups, (8) sex, (9) location of original studies, (10) definition of injuries, (11) study design of original research, (12) type of IPPs and main type of physical exercise reviewed, (13) observation period of intervention, (14) the percentage of compliance, (15) type of control group. This information was calculated if it was not directly reported and the authors of systematic reviews included sufficient information to calculate it. In addition, we extracted from meta-analyses of interest the following information: (1) statistical metric (e.g., risk ratio), (2) *p*-value, (3) I-square value, (3) the 95% confidence interval, (4) the number of studies meta-analyzed, (5) total sample meta-analyzed, (6) the certainty of evidence of meta-analysis using the GRADE system. Corresponding authors were not contacted to clarify or report additional information. Disagreements between JMC and SPE were solved by consensus. The percentage of agreement between these co-authors was calculated using the number of items rated with the same score before pooling the results of their independent assessments. The percentage of agreement was 90%.

### 2.8. Data Synthesis

The results are reported in the main text by sports and type of IPP (e.g., FIFA 11). Inside this category, we included all meta-analyses evaluating different musculoskeletal body regions. We also include in this category all subgroups that could be extracted from the reviews included if they satisfy our inclusion criteria. On the other hand, those meta-analyses of interest combining original research that studied different IPPs (e.g., FIFA 11 and a Nordic exercise program) are reported in tables, but they are not shown in the main text. This decision was made because we aimed to show those meta-analyses with the highest clinical replicability, which is essential to translating clinical research into clinical practice.

## 3. Results

**Figure 1:**
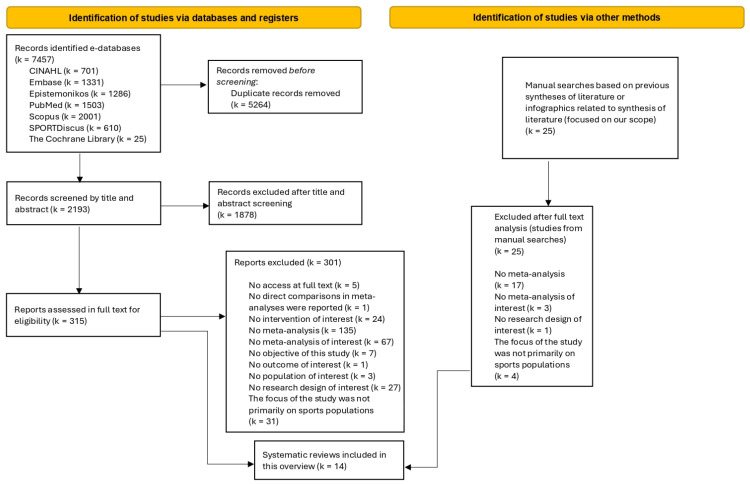
The PRISMA 2020 flow diagram.

A total of 7457 references were retrieved from e-databases. After removing duplicates, 2193 references were read and evaluated by title and abstract. Of them, 315 references were analyzed in full text. Finally, 14 systematic reviews with meta-analyses were included (Figure 1). The list of excluded studies with the reasons for exclusion during the analysis in the full text was reported in Supplementary File S3. Additionally, 25 references were manually found, and all were excluded (Supplementary File S4). Table 1 shows the characteristics of the included reviews [16,17,18,34,35,36,37,38,39,40,41,42,43,44].

### 3.1. Overlap Between the Included Meta-Analyses

**Figure 2:**
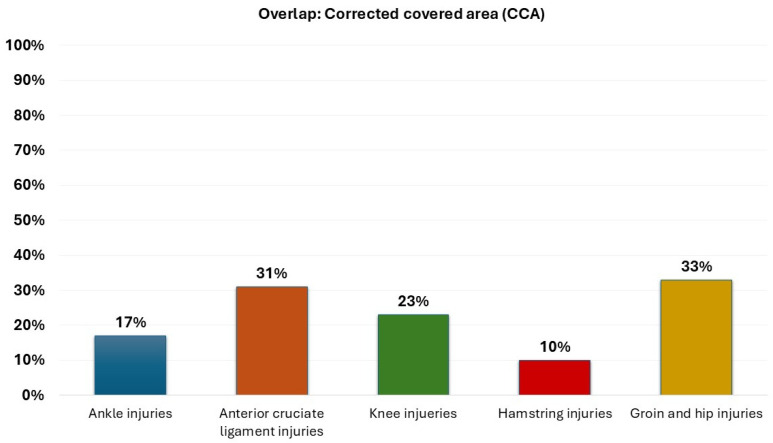
The degree of overlap by type of sport.

A very high overlap was found between meta-analyses evaluating IPPs for soccer players and anterior cruciate ligament injuries (CCA = 31%), knee injuries (CCA = 23%), hip/groin injuries (CCA = 33%), and ankle injuries (17%). On the other hand, the degree of overlap between meta-analyses evaluating IPPS for soccer players and hamstring injuries was moderate (CCA = 10%). The degree of overlap is depicted in Figure 2. Matrices of evidence and CCA calculations were reported in Supplementary Files S5–S9. We could not include a review in the overlap calculations since it did not specify the references that were included in the meta-analyses of interest [42].

### 3.2. Methodological Quality Assessment

The methodological quality of systematic reviews is reported in Table 2. For the most part, no reviews explained the reasons to choose a specific research design (e.g., randomized clinical trials) (item 3). No reviews included a list of excluded studies and justified the reasons for their exclusion (item 7), and no reviews reported the sources of funding for primary studies (item 10). In addition, eleven out of fourteen reviews did not assess the possible impact of the risk of bias in primary studies on the meta-analysis (item 12). The inter-rater agreement between JMS and SPE was 93.3%.

### 3.3. Injury Prevention Programs Focused on Physical Exercise for Sport-Related Musculoskeletal Injuries

Of the 14 systematic reviews included, only 3 reported meta-analyses that focused on a specific IPP. All these programs were developed for soccer players [17,43,44]. These meta-analyses will be reported below, and the remaining meta-analyses are shown in Table 1.

### 3.4. The Effects of FIFA 11+ on Sport-Related Musculoskeletal Injuries

#### 3.4.1. Ankle Injuries

One meta-analysis found that FIFA 11 + may reduce the risk of ankle injuries per 1000 h of exposure in comparison to multiple controls (undefined) (IRR 0.64, 95%CI 0.48–0.84; *p* = 0.002; I^2^ 36%; k = 5; N = UR) [17]. Another meta-analysis supported this result when FIFA 11+ was compared to no intervention or sham intervention (IRR 0.68; 95%CI 0.48–0.97; *p* = UR; I^2^ 27.1%; k = 3; N = UR), although this meta-analysis was not reported by 1000 h of exposure [43]. The GRADE system was not applied to rate the certainty of the evidence in any of these meta-analyses (Table 1).

#### 3.4.2. Knee Injuries

One meta-analysis found FIFA 11+ may reduce the risk of knee injuries in comparison to no intervention or sham intervention (IRR 0.52; 95%CI 0.38–0.72; *p* = UR; I^2^ 0%; k = 4; N = UR) [43]. The GRADE system was not applied to rate the certainty of the evidence in this meta-analysis (Table 1).

#### 3.4.3. Hip/Groin Injuries

One meta-analysis found FIFA 11+ may reduce the risk of hip/groin injuries in comparison to no intervention or sham intervention (IRR 0.59; 95%CI 0.35–0.97; *p* = UR; I^2^ 0%; k = 2; N = UR) [43]. The GRADE system was not applied to rate the certainty of the evidence in this meta-analysis (Table 1).

#### 3.4.4. Hamstring Injuries

One meta-analysis found FIFA 11+ may reduce the risk of hamstring injuries in comparison to no intervention or sham intervention (IRR 0.40; 95%CI 0.19–0.84; *p* = UR; I^2^ 0%; k = 2; N = UR) [43]. The GRADE system was not applied to rate the certainty of the evidence in this meta-analysis (Table 1).

### 3.5. The Effects of FIFA 11+ Kids on Sport-Related Musculoskeletal Injuries

#### 3.5.1. Ankle Injuries

One meta-analysis found that FIFA 11 + kids may reduce the risk of ankle injuries in comparison to standardized warm-up (undefined) (RR 0.56; 95%CI 0.35–0.89; *p* = 0.01; I^2^ 0%; k = 4; N = 3160) [44]. The authors of this review rated the certainty of the evidence of this meta-analysis using the GRADE system as moderate because the evidence was downgraded due to the risk of bias (Table 1).

#### 3.5.2. Knee Injuries

One meta-analysis found that FIFA 11 + kids may reduce the risk of knee injuries in comparison to standardized warm-up (undefined) (RR 0.45; 95%CI 0.29–0.72; *p* = 0.0009; I^2^ 0%; k = 4; N = 3160) [44]. The authors of this review rated the certainty of the evidence of this meta-analysis using the GRADE system as moderate because the evidence was downgraded due to the risk of bias (Table 1).

## 4. Discussion

The present overview of systematic reviews with meta-analyses aimed to summarize the pooled effects of IPPs focused on physical exercise in reducing the risk and incidence of musculoskeletal injuries and reinjuries in different sports. To show the reader those meta-analyses that could have a greater translation into clinical practice, 14 systematic reviews with meta-analyses were included in this study. All these reviews developed meta-analyses by sport and by musculoskeletal body regions. Within these reviews, we tried to go a step further to provide the reader, mainly sports clinicians, with data that could have a greater clinical replication, and we showed in the main text only those meta-analyses that had included randomized clinical trials examining the same IPPs. In this sense, three of the 14 included reviews evaluated the effects of FIFA 11+ or FIFA 11+ kids, showing positive effects in favor of these two programs to reduce the risk of ankle, knee, hamstring, and hip/groin injuries among soccer players.

Despite these results, one of the major findings of this overview is the huge research gap that still exists regarding the development of meta-analyses evaluating the effects of specific IPPs by sport and musculoskeletal body regions. One of the main surprises in our results is the homogeneity of the included reviews considering the sports analyzed. Of the 14 reviews included, 13 reviews were mainly focused on soccer. Furthermore, we did not find systematic reviews with meta-analyses specifically focused on sports such as water polo, swimming, hockey, tennis, volleyball, or futsal, after analyzing more than 300 full-text studies (see Supplementary File S3).

It is also important to underline the lack of specific meta-analyses on certain musculoskeletal body regions, especially those related to the upper limb. We did not find meta-analyses that met our criteria for evaluating the effects of IPPs on the shoulder, elbow, wrist, or hand. In addition, we were able to find meta-analyses evaluating specific clinical conditions such as anterior cruciate ligament injuries. However, the lack of meta-analyses meeting our inclusion criteria for examining other clinical conditions as important and common in sports as ankle sprains, or quadriceps injuries was surprising.

**Figure 3:**
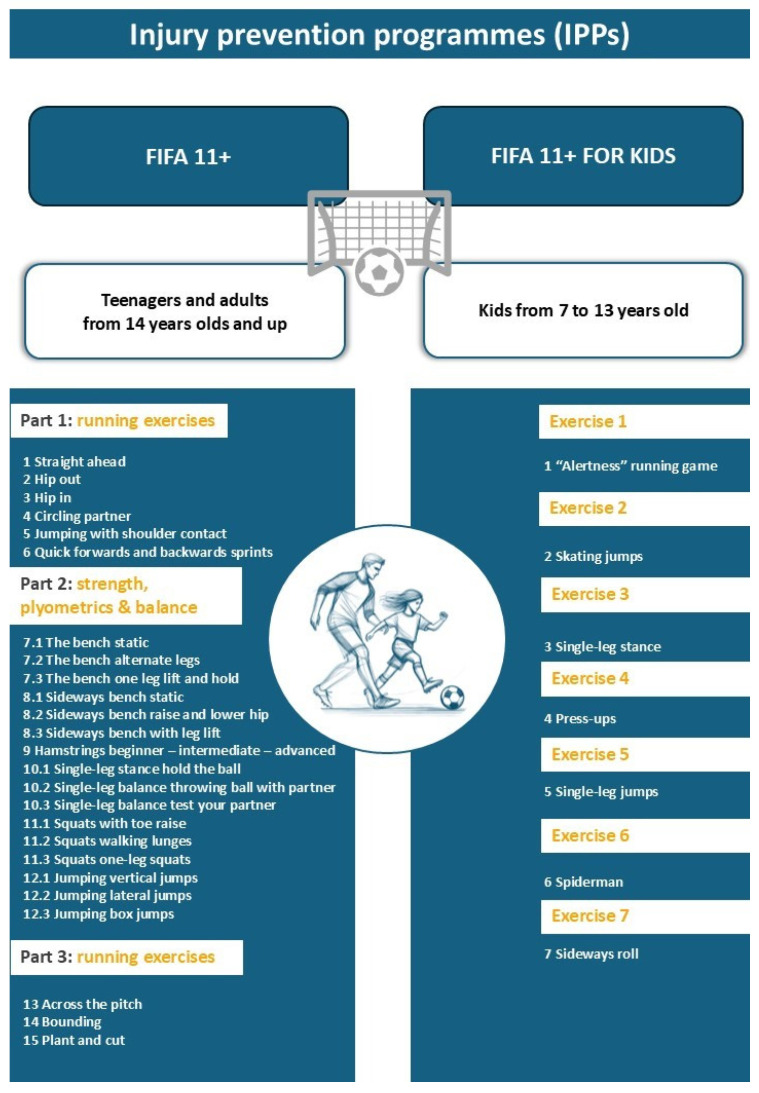
IPPs FIFA11+ and FIFA11 + kids characteristics.

It is very positive to have systematic reviews with meta-analyses whose results can be brought closer to clinical practice. As we have previously mentioned, we have reviews available that have highlighted the effects of FIFA 11+ and FIFA 11+ kids (Figure 3). However, many other IPPs could be applied to soccer players or other athletes. Programs such as the Knäkontroll program, the Prevent Injury, and Enhance Performance program, or the Core Position and Control movement strategy warm-up could produce benefits for improving different sport-related musculoskeletal injuries. A recent network meta-analysis has highlighted the importance of some of these programs to reduce the risk of anterior cruciate ligament injuries in soccer players [30].

There is current information to show that sex and age-based differences exist in the development of sports injuries [45,46]. However, we do not have information to show relevant clinical messages in this regard. Although we found IPPs focused on specific age populations (e.g., FIFA 11+ kids), the analyses were not separated between children and adolescents, despite studies that included soccer players aged 7–14 years [44]. Furthermore, we found some meta-analyses that examined differences by sex, but none of these analyses were carried out by type of IPP (Table 1).

### 4.1. Limitations

We acknowledge that because our inclusion criteria were very strict, many systematic reviews related to our topic were not included or thoroughly analyzed. This does not mean that these reviews are not of good quality, nor that they are methodologically not well conducted. We only aim to show those meta-analyses that have the greatest number of available characteristics (mainly, type of sport, musculoskeletal body region, and type of IPP) that would allow us to provide information with the greatest possible clinical applicability. In this regard, we encourage readers to consult Table 1 and our Supplementary File S3 for more information. In addition, the scarce data of IPPs on other sports rather than soccer preclude us perform clinical messages for many sports. Finally, an important methodological aspect needs to be acknowledged. Overlap analysis was conducted only when at least two systematic reviews analyzed the same sport and musculoskeletal body region. Although this approach allows us to avoid underestimating overlap analysis including mixing related reviews but with different analyses, it is true that this approach could omit overlap between reviews analyzing related but not identical categories.

### 4.2. Future Research Recommendations

The following points may help to improve the quality of research in this field. First, we found some systematic reviews covering some sports that could not be included in this overview because these reviews did not perform meta-analysis (Supplementary File S3). Therefore, we encourage sports researchers to update these systematic reviews, performing, if possible, different quantitative analyses (e.g., meta-analysis) that allow us to have specific information about the effects of IPPs in this field. We also encourage sports researchers to continue developing primary studies and systematic reviews that allow an increase in the body of knowledge considering the effects of IPPs on different musculoskeletal regions, especially in upper limbs. In addition, future systematic reviews with meta-analyses need to develop subgroups by type of IPP to know the real effects of each of these programs. Finally, we detected that the degree of overlap between meta-analyses of interest was very high in most of musculoskeletal regions or clinical conditions evaluated, and most of the meta-analyses examined less than 10 randomized clinical trials (Table 1). Therefore, we wonder if it would be necessary to first develop some high-quality randomized clinical trials in this field that could allow the development of more subgroup analyses that would help us to know results that could have a greater extrapolation to clinical practice. Furthermore, clinical recommendations are usually more associated with clinical practice guidelines than systematic reviews, but clinical practice guidelines often nurture systematic reviews, and many clinicians consume direct systematic reviews with meta-analyses for their decision-making. In this context, it would be very interesting if the authors of future systematic reviews evaluating IPPs focused on physical exercise use the Consensus on Exercise Reporting Template to show whether the reviewed studies have reported their interventions in sufficient detail to be replicated [47].

## 5. Conclusions

This overview of systematic reviews with meta-analyses has shown that the FIFA 11+ and FIFA 11+ kids’ IPPs could be beneficial in reducing the risk of ankle, knee, hip/groin, and hamstring injuries among soccer players. However, this overview has also found huge gaps regarding the pooled effects of specific IPPs on different sports and concrete musculoskeletal body regions, which may call for the sports community to increase the body of knowledge on this topic.

## Figures and Tables

**Table 1:** Characteristics of the included systematic reviews.

Study and Year of Publication	Population (General Review)	Study Design Original Research (General Review)	Intervention Group (General Review)	Control Group (General Review)	Meta-Analyses of Interest and the Certainty of Evidence (GRADE)
Al Attar et al., 2022 [17]	N: 7828 Hours of exposure: 863.7 The number of injuries: 451 knee injuries Mechanism of injury: non-contact injuries Sport: soccer Level of play: collegiate or amateur Age groups: adolescents and adults (ages ranged from 12 to 45 years) Sex: females and males Location: Africa, Europe, Oceania, and the Americas Did the authors provide a specific definition of injuries? Yes	Nine cluster randomized controlled trials	Types of IPP: FIFA 11, FIFA 11+, neuromuscular warm-up, pre-training and post-training FIFA 11+ Observation period: interventions ranged from 6 months to 12 months Compliance: It ranged from 47% to 85%	Usual care (undefined), Pre-training FIFA 11+ program	**Knee injuries** Overall reduction in risk of knee injuries per 1000 h of exposure in favor of the intervention group (IRR 0.446; 95%CI 0.321–0.619; *p* = 0.000; I^2^ 29%; k = 9; N = 813,952) **Anterior cruciate ligament injuries** Overall reduction in risk of anterior cruciate ligament injuries per 1000 h of exposure in favor of the intervention group (IRR 0.401; 95%CI 0.215–0.750; *p* = 0.004; I^2^ 0%; k = 4; N = 557,302) **Subgroup analyses by sex: knee injuries** Reduction in risk of knee injuries per 1000 h of exposure in males (IRR 0.537; 95%CI 0.355–0.813; *p* = 0.003; I^2^ 16.65%; k = 5; N = 354,531) and females (IRR 0.354; 95% CI 0.221–0.565; *p* = 0.001; I^2^ 19.88%; k = 3; N = 411,773) **The certainty of evidence:** the GRADE system was not applied
Al Attar et al., 2022b [16]	N: 9633 Hours of exposure: 775,606 The number of injuries: 529 ankle injuries Mechanism of injury: UR Sport: soccer Level of play: middle and high school, collegiate, amateur, or elite (e.g., Norwegian First, Second, and Third Division) Age groups: children, adolescents, and adults (ages ranged from 7 to 35 years) Sex: females and males Location: Africa, Europe, Oceania, and the Americas Did the authors provide a specific definition of injuries? Yes	Eight cluster randomized controlled trials and one individual randomized controlled trial	Types of IPP: FIFA 11, FIFA 11+, FIFA 11+ kids, pre-training and post-training FIFA 11+, soccer-specific neuromuscular training program, targeted exercise program including balance exercise, balance training program Observation period: interventions ranged from 2.5 months to 12 months Compliance: It ranged from 28% to 95/100%	Pre-training FIFA 11+ program, standardized warm-up, home-based stretching program, neuromuscular training, Nordic hamstring lowers and groin strength training, resisted running using elastic bands, standard conditioning exercises, without any balance training exercises, aerobic warm-up, static and/or dynamic stretches, and soccer skills practice, small-sided games	**Ankle injuries** Overall reduction in risk of ankle injuries per 1000 h of exposure in favor of the intervention group (IRR 0.64, 95%CI 0.54–0.77; *p* = 0.000; I^2^ 0%; k = 9; N = UR) **Subgroup analyses by sex: ankle injuries** Reduction in risk of ankle injuries per 1000 h of exposure in males (IRR 0.58, 95%CI 0.45–0.76; *p* = 0.000; I^2^ 0%; k = 4; N = UR) or trials including males and females (IRR 0.59, 95%CI 0.42–0.83; *p* = 0.002; I^2^ 0%; k = 3; N = UR) No differences between groups were observed in females (IRR 0.85, 95%CI 0.59–1.22; *p* = 0.377; I^2^ 0%; k = 2; N = UR) **Subgroup analyses by type of intervention: ankle injuries** FIFA 11 +: reduction in risk of ankle injuries per 1000 h of exposure (IRR 0.64, 95%CI 0.48–0.84; *p* = 0.002; I^2^ 36%; k = 5; N = UR) Balance training exercises alone: reduction in risk of ankle injuries per 1000 h of exposure (IRR 0.59, 95%CI 0.41–0.84; *p* = 0.004; I^2^ 0%; k = 4; N = UR) **The certainty of evidence:** the GRADE system was not applied
Al Attar et al., 2023 [34]	N: 14,063 Hours of exposure: 417,189 The number of injuries: 332 knee injuries Mechanism of injury: UR Sports: soccer and handball Level of play: recreational, amateur, or elite Age groups: adolescents and adults (ages ranged from 12 to 45 years) Sex: females and males Location: Africa, Europe, Oceania, and the Americas Did the authors provide a specific definition of injuries? Yes	Eight cluster randomized controlled trials and two individual randomized controlled trial	Types of IPP: FIFA 11+, pre-training and post-training FIFA 11+, neuromuscular warm-up program, soccer specific neuromuscular training program, multicomponent exercise program Observation period: interventions ranged from 6 months to 12 months Compliance: It ranged from 60% to 98%	Null-standard warm-up, home-based stretching program, pre-training FIFA 11+ IPP only	**Subgroup analyses by sports: Knee injuries** Overall reduction in risk of knee injuries per 1000 h of exposure in favor of the intervention group (IRR 0.446, 95%CI 0.321–0.619; *p* = 0.001; I^2^ 29.142%; k = 9; N = 813,952) **The certainty of evidence:** the GRADE system was not applied
Al Attar et al., 2023b [35]	N: 4485 Hours of exposure: 379,102 The number of injuries: 171 hamstring injuries Mechanism of injury: UR Sports: soccer Level of play: collegiate, amateur, or elite Age groups: adolescents and adults (ages ranged from 13 to 40 years) Sex: females and males Location: Europe, Oceania, and the Americas Did the authors provide a specific definition of injuries? Yes	Four cluster randomized controlled trials and one individual randomized controlled trial	Types of IPP: FIFA 11+, pre-training and post-training FIFA 11+, targeted exercise program Observation period: interventions ranged from 2.5 to 8 months Compliance: It ranged from 28% to 91%	Pre-training FIFA 11+ IPP only, neuromuscular training, Nordic hamstring lowers, groin strength training, aerobic warm-up, static and/or dynamic stretching, soccer skills practice, standard warm-up (e.g., aerobic training plus stretching exercises)	**Hamstring injuries** Overall reduction in risk of hamstring injuries per 1000 h of exposure in favor of the intervention group (IRR 0.53, 95%CI 0.28–0.98; *p* = 0.04; Tau^2^ 0.300; k = 5; N = 379,102) **The certainty of evidence:** the GRADE system was not applied
Alexander et al., 2022 [36]	N: 11,137 Hours of exposure: UR The number of injuries: UR knee injuries Mechanism of injury: UR Sports: Runners Level of play: novice and recreational runners, mainly Age groups: adults (ages approximately ranged from 28 to 43 years) Sex: females and males Location: Asia, Europe, Oceania, the Americas Did the authors provide a specific definition of injuries? The authors reported as Supplementary Material the definitions used by the included original research This information was associated with studies evaluating prevention.	Eighteen randomized clinical trials, including two pilot randomized clinical trials This information was associated with studies evaluating prevention.	Types of IPP: foot and ankle muscle strengthening program, resistance strength training, gait retraining Only those IPP directly related to physical exercise have been included above. Observation period: interventions ranged from 8 weeks to 12 months of follow-up Compliance: It could reach up to more than 90% in one study, but it decreased as the follow-up progressed	Static stretching, functional strengthening training	**Subgroup analyses by specific type of intervention: knee injuries** No differences statistically significant were observed between groups regarding multicomponent exercise therapy focused on strengthening exercises (RR 0.56, 95%CI 0.05–6.42; *p* = 0.64; I^2^ 65%; k = 2; N = 163) **The certainty of evidence:** very low certainty of evidence using the GRADE system: downgraded evidence because risk of bias, inconsistency, imprecision, and publication bias strongly suspected
Avila-Quintero et al., 2024 [37]	N: 4793 Hours of exposure: UR The number of injuries: UR hamstring injuries Mechanism of injury: UR Sports: Soccer Level of play: elite and non-elite Age groups: adolescents and adults (ages approximately ranged from 15 to 41 years) Sex: females and males Location: Europe, Asia, Oceania, Africa Did the authors provide a specific definition of injuries? No	Ten randomized controlled trials	Types of IPP: Nordic exercises alone or plus different strategies such as usual care or eccentric exercises with bands Observation period: interventions ranged from 10 weeks to 12 months to the full soccer season Compliance: UR	FIFA 11 + program, usual care, previous team data	**Hamstring injuries** Reduction in risk of hamstring injuries in favor of the intervention group (OR 0.376, 95%CI 0.241–0.586; *p* < 0.001; I^2^ 66%; k = 9; N = UR) **Subgroup analyses by sex: hamstring injuries** There was a higher reduction in risk of hamstring injuries in males (OR 0.454, 95%CI 0.341–0.604; *p* < 0.001; I^2^ UR; k = UR; N = UR) than females (OR 0.587, 95%CI 0.460–0.750; *p* = 0.004; I^2^ UR; k = UR; N = UR) **Subgroup analyses by level of play: hamstring injuries** There was a higher reduction in risk of hamstring injuries in elite players (OR 0.321, 95%CI 0.146–0.702; *p* < 0.001; I^2^ UR; k = UR; N = UR) than non-elite players (OR 0.542, 95%CI 0.448–0.656; *p* < 0.001; I^2^ UR; k = UR; N = UR) **The certainty of evidence:** the GRADE system was not applied
Crossley et al., 2020 [38]	N: 11,773 Hours of exposure: 896,002 The number of injuries: 1321. In specific locations: 70 anterior cruciate ligament injuries, 455 knee injuries, 393 ankle injuries, 45 hip/groin injuries, 35 hamstring injuries Mechanism of injury: UR Sports: soccer Level of play: middle and high school, collegiate, or elite Age groups: adolescents and adults (ages approximately ranged from 11 to 23 years) Sex: females Location: Europe and the Americas Did the authors provide a specific definition of injuries? Yes (overall injuries were defined)	Twelve randomized controlled trials	Types of IPP: eccentric hamstring exercises, FIFA 11 + kids, the CORE intervention (exercises focused on the trunk and lower extremity), neuromuscular training plus home-based balance training, the Prevent Injury and Enhance Performance program, Frappier Acceleration training program, home-based balance board training, FIFA 11, FIFA 11+, using a lighter small football, Knäkontroll Observation period: one season/year Compliance: Almost half of the studies reported an adherence higher than 78%	Specific control groups (undefined)	**Anterior cruciate ligament injuries** No differences statistically significant were observed between groups when multicomponent and individual exercise programs were combined in the analysis (IRR 0.62, 95%CI 0.37–1.05; *p* = UR; I^2^ 0%; k = 6; N = UR) However, multicomponent exercise programs alone showed a higher reduction in risk of anterior cruciate ligament injuries compared to control groups (IRR 0.55, 95%CI 0.32–0.92; *p* = UR; I^2^ 0%; k = 5; N = UR) **The certainty of evidence:** low certainty of evidence using the GRADE system in both analyses: downgraded evidence because risk of bias and imprecision **Hip/groin injuries** No differences statistically significant were observed between groups when multicomponent or individual exercise programs were combined in the analysis (IRR 0.75, 95%CI 0.41–1.40; *p* = UR; I^2^ 0%; k = 5; N = UR) No differences statistically significant were observed between groups when multicomponent exercise alone were analyzed (IRR 0.71, 95%CI 0.38–1.33; *p* = UR; I^2^ 0%; k = 4; N = UR) **The certainty of evidence:** low certainty of evidence using the GRADE system in both analyses: downgraded evidence because risk of bias and imprecision **Hamstring injuries** Reduction in risk of hamstring injuries in favor of the intervention group when multicomponent and individual exercise programs were combined in the analysis (IRR 0.40, 95%CI 0.17–0.95; *p* = UR; I^2^ 0%; k = 4; N = UR) No differences statistically significant were observed between groups when multicomponent exercise alone were analyzed (IRR 0.60, 95%CI 0.21–1.71; *p* = UR; I^2^ 0%; k = 2; N = UR) **The certainty of evidence:** low certainty of evidence using the GRADE system in both analyses: downgraded evidence because risk of bias and imprecision **Knee injuries** No differences statistically significant were observed between groups when multicomponent or individual exercise programs were combined in the analysis (IRR 0.85, 95%CI 0.67–1.09; *p* = UR; I^2^ 21.9%; k = 10; N = UR) No differences statistically significant were observed between groups when multicomponent exercise alone were analyzed (IRR 0.83, 95%CI 0.65–1.06; *p* = UR; I^2^ 23.2%; k = 9; N = UR) **The certainty of evidence:** low certainty of evidence using the GRADE system in both analyses: downgraded evidence because risk of bias and imprecision **Ankle injuries** No differences statistically significant were observed between groups when multicomponent or individual exercise programs were combined in the analysis (IRR 0.83, 95%CI 0.65–1.07; *p* = UR; I^2^ 13.1%; k = 8; N = UR) No differences statistically significant were observed between groups when multicomponent exercise alone were analyzed (IRR 0.78, 95%CI 0.58–1.05; *p* = UR; I^2^ 23.2%; k = 7; N = UR) **The certainty of evidence:** low certainty of evidence using the GRADE system in both analyses: downgraded evidence because risk of bias and imprecision
Grimm et al., 2015 [39]	N: 11,562 Hours of exposure: UR The number of injuries: 459 knee injuries and approximately 60 anterior cruciate ligament injuries Mechanism of injury: UR Sports: soccer Level of play: UR Age groups: adolescents and adults (ages approximately ranged from 12 to 37 years) Sex: females and males Location: UR Did the authors provide a specific definition of injuries? Yes	Eight cluster randomized controlled trials and one individual randomized controlled trial	Types of IPP: balance training, multicomponent exercise program Observation period: approximately ranged from 3 months to 12 months Compliance: UR	Specific control groups (undefined)	**Knee injuries** Overall reduction in risk of knee injuries in favor of the intervention group (RR 0.74; 95%CI 0.55–0.98; *p* = 0.041; I^2^ 50.2%; k = 9; N = UR) **Anterior cruciate ligament injuries** No differences statistically significant were observed between groups (RR 0.66; 95%CI 0.33–1.32; *p* = 0.222; I^2^ 31.7%; k = 4; N = UR) **The certainty of evidence:** the GRADE system was not applied
Grimm et al., 2016 [40]	N: 4121 Hours of exposure: UR The number of injuries: 348 ankle injuries Mechanism of injury: UR Sports: soccer Level of play: recreational and elite Age groups: adolescents and adults (ages approximately ranged from 13 to 37 years) Sex: females and males Location: UR Did the authors provide a specific definition of injuries? Yes	Six cluster randomized controlled trials and three individual randomized controlled trials	Types of IPP: balance training, multicomponent exercise program Observation period: approximately ranged from 6 to 12 months Compliance: UR	Specific control groups (undefined)	**Ankle injuries** Overall reduction in risk of ankle injuries in favor of the intervention group (RRs 0.60; 95%CI 0.40–0.92; *p* = 0.002; I^2^ 65.2%; k = 9; N = UR) **The certainty of evidence:** the GRADE system was not applied
Lemes et al., 2021 [18]	N: 13,355 Hours of exposure: 1,062,711 h of exposure The number of injuries: 545 musculoskeletal injuries Mechanism of injury: non-contact injuries Sports: soccer Level of play: high school, collegiate, amateur Age groups: adolescents and adults (ages approximately ranged from 14 to 45 years) Sex: females and males Location: Africa, Asia, Europe, the Americas Did the authors provide a specific definition of injuries? Yes	Ten randomized controlled trials	Types of IPP: the Prevent Injury and Enhance Performance Programme, FIFA 11, FIFA 11+, focused Nordic Hamstring Exercise, focused Bounding Exercise Programme, Knäkontroll These interventions were divided into focused or general programs; focused programs were based on quadriceps or hamstring exercises. General programs were based on agility, balance, mobility, plyometrics, running, and strength exercises for the lower limb. Observation period: approximately ranged from 12 weeks to 9 months Compliance: UR	Instructions to carry out standard warm-up exercises, usual training programs	**Hamstring injuries** No differences statistically significant were observed between groups (IRR 0.65; 95%CI 0.42–1.00; *p* = 0.05; I^2^ 16%; k = 6; N = UR) **Subgroup analyses by type of intervention: hamstring injuries** No differences statistically significant were observed regarding general exercise programs (e.g., agility) (IRR 0.63; 95%CI 0.19–2.12; *p* = 0.45; I^2^ 51%; k = 3; N = 396,549) **The certainty of evidence:** very low certainty of evidence using the GRADE system: downgraded evidence because of risk of bias, inconsistency, indirectness, and imprecision On the other hand, an overall reduction in risk of hamstring injuries was observed in favor of focused exercise programs (IRR 0.65; 95%CI 0.44–0.97; *p* = 0.03; I^2^ 0%; k = 3; N = 182,051) **The certainty of evidence:** low certainty of evidence using the GRADE system: downgraded evidence because of risk of bias and imprecision
Obërtinca et al., 2023 [41]	N: 22,177 Hours of exposure: 1,587,327 h of exposure The number of injuries: 5080 injuries Mechanism of injury: contact and non-contact Sports: soccer Level of play: amateur and elite Age groups: children, adolescents, and adults (ages approximately ranged from 7 to 45 years) Sex: females and males Location: Africa, Asia, Europe, Oceania, and the Americas Did the authors provide a specific definition of injuries? No	Fifteen randomized or cluster randomized controlled trials	Types of IPP: neuromuscular training program, FIFA 11+, FIFA 11+ kids, FIFA 11, Knäkontroll, bounding exercise program, Prevent Injury and Enhance Performance Program Observation period: approximately ranged from 12 weeks to 9 months (39 weeks) Compliance: UR	Standard warm-up exercises and/or training routines	**Ankle injuries** Overall reduction in risk of ankle injuries in favor of the intervention group (RR 0.73; 95%CI 0.55–0.96; *p* = UR; I^2^ 41.4%; k = 7; N = 508) **The certainty of evidence:** moderate certainty of evidence using the GRADE system: downgraded evidence because of risk of bias **Hamstring injuries** No differences statistically significant were observed between groups (RR 0.83; 95%CI 0.50–1.37; *p* = UR; I^2^ 0%; k = 2; N = 70) **The certainty of evidence:** low certainty of evidence using the GRADE system: downgraded evidence because of risk of bias and imprecision **Hip and groin injuries** No differences statistically significant were observed between groups (RR 0.56; 95%CI 0.30–1.05; *p* = UR; I^2^ 21.1%; k = 3; N = 59) **The certainty of evidence:** low certainty of evidence using the GRADE system: downgraded evidence because of risk of bias and imprecision **Knee injuries** Overall reduction in risk of knee injuries in favor of the intervention group (RR 0.69; 95%CI 0.52–0.90; *p* = UR; I^2^ 52.4%; k = 11; N = 725) **The certainty of evidence:** low certainty of evidence using the GRADE system: downgraded evidence because of risk of bias and inconsistency
Ripley et al., 2021 [42]	N: 6906 Hours of exposure: no individual or pooled information was provided The number of injuries: approximately 336 injuries Mechanism of injury: UR Sports: soccer Level of play: high school, collegiate, amateur, and elite Age groups: UR, but children <10 years were not considered Sex: females and males Location: this systematic review did not report on the location for all included original research, but some continents could be extracted: Europe, Oceania, and the Americas Did the authors provide a specific definition of injuries? No	Thirteen randomized controlled trials	Types of IPP: Nordic hamstring exercises, eccentric training, FIFA 11+, bounding exercise program, FIFA 11 warm-up, FIFA 11+ post-training, Observation period: one season was indicated in much included original research Compliance: It ranged from 21.1% to 100%	Specific control groups (undefined)	**Hamstring injuries** Overall reduction in risk of hamstring injuries in favor of the intervention group (log OR −0.61; 95%CI −1.05–0.17; *p* = 0.007; I^2^ 67.66%; k = UR; N = UR) **The certainty of evidence:** the GRADE system was not applied The authors reported at the end of their results that some types of IPP may be more effective than others, but no meta-analysis data was provided, and no Supplementary Materials were reported.
Thorborg et al., 2017 [43]	N: 6574 Hours of exposure: 510,055 The number of injuries: 2454 injuries Mechanism of injury: UR Sports: soccer Level of play: recreational/subelite Age groups: adolescents and adults (ages approximately ranged from 13 to 40 years) Sex: females and males Location: UR Did the authors provide a specific definition of injuries? Yes	Six cluster randomized controlled trials	Types of IPP: FIFA 11 and FIFA 11+ Only information about FIFA 11+ was included in meta-analyses evaluating hamstring, hip/groin, knee, and ankle injuries Observation period: it ranged from 5 months to 9 months Compliance: Although compliance was evaluated in this review, we did not find information concerning compliance for each included trial or a pooled percentage of this compliance (including all trials)	No intervention or sham intervention	**Hamstring injuries** Overall reduction in risk of hamstring injuries in favor of the intervention group (IRR 0.40; 95%CI 0.19–0.84; *p* = UR; I^2^ 0%; k = 2; N = UR) **Hip/groin injuries** Overall reduction in risk of hip/groin injuries in favor of the intervention group (IRR 0.59; 95%CI 0.35–0.97; *p* = UR; I^2^ 0%; k = 2; N = UR) **Knee injuries** Overall reduction in risk of knee injuries in favor of the intervention group (IRR 0.52; 95%CI 0.38–0.72; *p* = UR; I^2^ 0%; k = 4; N = UR) **Ankle injuries** Overall reduction in risk of ankle injuries in favor of the intervention group (IRR 0.68; 95%CI 0.48–0.97; *p* = UR; I^2^ 27.1%; k = 3; N = UR) **The certainty of evidence:** the GRADE system was not applied
Yang et al., 2022 [44]	N: 10,565 Hours of exposure: UR The number of injuries: 442 injuries Mechanism of injury: UR Sports: soccer Level of play: amateur, subelite, or elite Age groups: children and adolescents (ages approximately ranged from 7 to 14 years) Sex: females and males Location: Asia and Europe Did the authors provide a specific definition of injuries? Yes	Five cluster randomized controlled trials and one individual randomized controlled trial	Types of IPP: FIFA 11 + kids Observation period: it ranged from 3 months to 10 months Compliance: UR	Standard warm-up	**Knee injuries** Overall reduction in risk of knee injuries in favor of the intervention group (RR 0.45; 95%CI 0.29–0.72; *p* = 0.0009; I^2^ 0%; k = 4; N = 3160) **The certainty of evidence:** moderate certainty of evidence using the GRADE system: downgraded evidence because of risk of bias **Ankle injuries** Overall reduction in risk of ankle injuries in favor of the intervention group (RR 0.56; 95%CI 0.35–0.89; *p* = 0.01; I^2^ 0%; k = 4; N = 3160) **The certainty of evidence:** moderate certainty of evidence using the GRADE system: downgraded evidence because of risk of bias

Note: 95%CrI: credible interval (confidence interval in Bayesian); 95%CI: confidence interval; FIFA: Fédération Internationale Football Association; GRADE: Grading of Recommendations, Assessment, Development, and Evaluations; IPP: injury prevention program; IRR: injury risk ratio; OR: odds ratio; RR: risk ratio; RRs: relative risk; UR: unreported.

**Table 2:** The methodological quality of reviews (AMSTAR 2).

Author(s)	1	2	3	4	5	6	7	8	9	10	11	12	13	14	15	16
Al Attar et al., 2022a [17]																
Al Attar et al., 2022b [16]																
Al Attar et al., 2023a [34]																
Al Attar et al., 2023b [35]																
Alexander et al., 2022 [36]																
Avila-Quintero et al., 2024 [37]																
Crossley et al., 2020 [38]																
Grimm et al., 2015 [39]																
Grimm et al., 2016 [40]																
Lemes et al., 2021 [18]																
Obërtinca et al., 2023 [41]																
Ripley et al., 2021 [42]																
Thorborg et al., 2017 [43]																
Yang et al., 2022 [44]																

Note: Answers: red color: no; yellow color: partially yes; green color: yes. Items: AMSTAR 1: Did the research questions and inclusion criteria for the review include the components of PICO? AMSTAR 2: Did the report of the review contain an explicit statement that the review methods were established prior to the conduct of the review and did the report justify any significant deviations from the protocol? AMSTAR 3: Did the review authors explain their selection of the study designs for inclusion in the review? AMSTAR 4: Did the review authors use a comprehensive literature search strategy? AMSTAR 5: Did the review authors perform study selection in duplicate? AMSTAR 6: Did the review authors perform data extraction in duplicate? AMSTAR 7: Did the review authors provide a list of excluded studies and justify the exclusions? AMSTAR 8: Did the review authors describe the included studies in adequate detail? AMSTAR 9: Did the review authors use a satisfactory technique for assessing the risk of bias in individual studies that were included in the review? AMSTAR 10: Did the review authors report on the sources of funding for the studies included in the review? AMSTAR 11: If meta-analysis was performed did the review authors use appropriate methods for statistical combination of results? AMSTAR 12: If meta-analysis was performed, did the review authors assess the potential impact of bias in individual studies on the results of the meta-analysis or other evidence synthesis? AMSTAR 13: Did the review authors account for the risk of bias in individual studies when interpreting/ discussing the results of the review? AMSTAR 14: Did the review authors provide a satisfactory explanation for, and discussion of, any heterogeneity observed in the results of the review? AMSTAR 15: If they performed quantitative synthesis did the review authors carry out an adequate investigation of publication bias (small study bias) and discuss its likely impact on the results of the review? AMSTAR 16: Did the review authors report any potential sources of conflict of interest, including any funding they received for conducting the review?

## Data Availability

No new data were created or analyzed in this study. Data sharing is not applicable to this article. All data relevant to the study are included in the article or are available as Supplementary Files.

## Supplementary Materials

The following supporting information can be downloaded at https://www.mdpi.com/article/10.3390/healthcare13131530/s1, Supplementary Files S1–S9.

## Abbreviations

The following abbreviations are used in this manuscript:

AMSTAR	Assessing the Methodological Quality of Systematic Reviews
GRADE	Grading of Recommendations Assessment, Development, and Evaluation
INPLASY	International Platform of Registered Systematic Review and Meta-analysis Protocols
IPPS	Injury prevention programs.
PRIOR	Preferred Reporting Items for Overviews of Reviews.
PRISMA	Preferred Reporting Items for Systematic Reviews and Meta-Analyses

## References

[1] Gimigliano F., Resmini G., Moretti A., Aulicino M., Gargiulo F., Gimigliano A., Liguori S., Paoletta M., Iolascon G. (2021). Epidemiology of Musculoskeletal Injuries in Adult Athletes: A Scoping Review. Med. Kaunas Lith..

[2] Yeich A., Shafeek P., Kumar K., Olympia R.P., Ceasar J.A. (2024). The Impact of Knee and Ankle Injuries on National Basketball Association Player Performance Post-injury. Cureus.

[3] Gouttebarge V., Aoki H., Ekstrand J., Verhagen E.A.L.M., Kerkhoffs G.M.M.J. (2016). Are severe musculoskeletal injuries associated with symptoms of common mental disorders among male European professional footballers?. Knee Surg. Sports Traumatol. Arthrosc. Off J. ESSKA.

[4] Maffulli N., Longo U.G., Gougoulias N., Caine D., Denaro V. (2010). Sport injuries: A review of outcomes. Br. Med. Bull..

[5] Rudisill S.S., Kucharik M.P., Varady N.H., Martin S.D. (2021). Evidence-Based Management and Factors Associated with Return to Play After Acute Hamstring Injury in Athletes: A Systematic Review. Orthop. J. Sports Med..

[6] Valle X., Alentorn-Geli E., Tol J.L., Hamilton B., Garrett W.E., Pruna R., Til L., Gutierrez J.A., Alomar X., Balius R. (2017). Muscle Injuries in Sports: A New Evidence-Informed and Expert Consensus-Based Classification with Clinical Application. Sports Med. Auckl. N. Z..

[7] Turnbull M.R., Gallo T.F., Carter H.E., Drew M., Toohey L.A., Waddington G. (2024). Estimating the cost of sports injuries: A scoping review. J. Sci. Med. Sport.

[8] Walia B., Boudreaux C.J. (2021). The cost of players’ injuries to professional sports leagues and other sports organizations. Manag. Finance.

[9] Stojanović E., Faude O., Nikić M., Scanlan A.T., Radovanović D., Jakovljević V. (2023). The incidence rate of ACL injuries and ankle sprains in basketball players: A systematic review and meta-analysis. Scand J Med Sci Sports.

[10] Maniar N., Carmichael D.S., Hickey J.T., Timmins R.G., Jose A.J.S., Dickson J., Opar D. (2022). Incidence and prevalence of hamstring injuries in field-based team sports: A systematic review and meta-analysis of 5952 injuries from over 7 million exposure hours. Br. J. Sports Med..

[11] Como M., Fatora G., Boden S.A., Reddy R.P., Njoku-Austin C., Nazzal E.M., Lin A. (2024). Shoulder Injury Incidence and Epidemiology in Youth, High School, and Collegiate Fastpitch Softball Players: A Systematic Review and Future Research Perspectives. Sports Health.

[12] Miller A.H., Evans K., Adams R., Waddington G., Witchalls J. (2018). Shoulder injury in water polo: A systematic review of incidence and intrinsic risk factors. J. Sci. Med. Sport.

[13] Lutter C., Jacquet C., Verhagen E., Seil R., Tischer T. (2022). Does prevention pay off? Economic aspects of sports injury prevention: A systematic review. Br. J. Sports Med..

[14] Cento A.S., Leigheb M., Caretti G., Penna F. (2022). Exercise and Exercise Mimetics for the Treatment of Musculoskeletal Disorders. Curr. Osteoporos. Rep..

[15] De la Corte-Rodriguez H., Roman-Belmonte J.M., Resino-Luis C., Madrid-Gonzalez J., Rodriguez-Merchan E.C. (2024). The Role of Physical Exercise in Chronic Musculoskeletal Pain: Best Medicine—A Narrative Review. Health.

[16] Al Attar W.S.A., Ghulam H.S., Al Arifi S., Akkam A.M., Alomar A.I., Sanders R.H. (2022). The effectiveness of injury prevention programs that include core stability exercises in reducing the incidence of knee injury among soccer players: A systematic review and meta-analysis. Isokinet. Exerc. Sci..

[17] A Al Attar W.S., Khaledi E.H., Bakhsh J.M., Faude O., Ghulam H., Sanders R.H. (2022). Injury prevention programs that include balance training exercises reduce ankle injury rates among soccer players: A systematic review. J. Physiother..

[18] Lemes I.R., Pinto R.Z., Lage V.N., Roch B.A.B., Verhagen E., Bolling C., Aquino C.F., Fonseca S.T., Souza T.R. (2021). Do exercise-based prevention programmes reduce non-contact musculoskeletal injuries in football (soccer)? A systematic review and meta-analysis with 13,355 athletes and more than 1 million exposure hours. Br. J. Sports Med..

[19] Lutz D., Berg C.v.D., Räisänen A.M., Shill I.J., Kim J., Vaandering K., Hayden A., Pasanen K., Schneider K.J., Emery A.C. (2024). Best practices for the dissemination and implementation of neuromuscular training injury prevention warm-ups in youth team sport: A systematic review. Br. J. Sports Med..

[20] Stephenson S.D., Kocan J.W., Vinod A.V., Kluczynski M.A., Bisson L.J. (2021). A Comprehensive Summary of Systematic Reviews on Sports Injury Prevention Strategies. Orthop. J. Sports Med..

[21] Andrew N., Gabbe B.J., Cook J., Lloyd D.G., Donnelly C.J., Nash C., Finch C.F. (2013). Could Targeted Exercise Programmes Prevent Lower Limb Injury in Community Australian Football?. Sports Med. Auckl. N. Z..

[22] Charlton P.C., Drew M.K., Mentiplay B.F., Grimaldi A., Clark R.A. (2017). Exercise Interventions for the Prevention and Treatment of Groin Pain and Injury in Athletes: A Critical and Systematic Review. Sports Med..

[23] Chen J., Zhang C., Chen S., Zhao Y. (2021). Effects of functional correction training on injury risk of athletes: A systematic review and meta-analysis. Peer J..

[24] Ding L., Luo J., Smith D.M., Mackey M., Fu H., Davis M., Hu Y. (2022). Effectiveness of Warm-Up Intervention Programs to Prevent Sports Injuries among Children and Adolescents: A Systematic Review and Meta-Analysis. Int. J. Environ. Res. Public Health.

[25] Hartling L., Chisholm A., Thomson D., Dryden D.M., Smalheiser N.R. (2012). A Descriptive Analysis of Overviews of Reviews Published between 2000 and 2011. PLoS ONE.

[26] Martínez-Calderon J. (2023). Overviews of systematic reviews in sports and exercise medicine: What are they and why are they important?. Br. J. Sports Med..

[27] Gates M., Gates A., Pieper D., Fernandes R.M., Tricco A.C., Moher D., E Brennan S., Li T., Pollock M., Lunny C. (2022). Reporting guideline for overviews of reviews of healthcare interventions: Development of the PRIOR statement. BMJ.

[28] Beller E.M., Glasziou P.P., Altman D.G., Hopewell S., Bastian H., Chalmers I., Gøtzsche P.C., Lasserson T., Tovey D. (2013). Prisma for Abstracts: Reporting systematic reviews in journal and conference abstracts. PLoS Med..

[29] Richardson W.S., Wilson M.C., Nishikawa J., Hayward R.S. (1995). The well-built clinical question: A key to evidence-based decisions. ACP J. Club.

[30] Magaña-Ramírez M., Gallardo-Gómez D., Álvarez-Barbosa F., Corral-Pernía J.A. (2024). What exercise programme is the most appropriate to mitigate anterior cruciate ligament injury risk in football (soccer) players? A systematic review and network meta-analysis. J. Sci. Med. Sport.

[31] Zech A., Hübscher M. (2012). Sensorimotor training for prevention of ankle sprain. Dtsch. Z. Sportmed..

[32] Shea B.J., Reeves B.C., Wells G., Thuku M., Hamel C., Moran J., Moher D., Tugwell P., Welch V., Kristjansson E. (2017). AMSTAR 2: A critical appraisal tool for systematic reviews that include randomised or non-randomised studies of healthcare interventions, or both. BMJ Online.

[33] Pieper D., Antoine S.-L., Mathes T., Neugebauer E.A., Eikermann M. (2014). Systematic review finds overlapping reviews were not mentioned in every other overview. J. Clin. Epidemiol..

[34] Al Attar W.S.A., Ghulam H., Al Arifi S., Alomar A.I., Alhosaini S., Alharbi S., Alraddadi Y., Sanders R.H. (2023). Injury prevention programs including balance exercises with compliance and follow-up reduce the incidence of knee injuries in athletes: A systematic review and meta-analysis. Isokinet. Exerc. Sci..

[35] Al Attar W.S.A., Husain M.A. (2023). Effectiveness of Injury Prevention Programs with Core Muscle Strengthening Exercises to Reduce the Incidence of Hamstring Injury Among Soccer Players: A Systematic Review and Meta-Analysis. Sports Health.

[36] Alexander J.L.N., Culvenor A.G., Johnston R.R.T., Ezzat A.M., Barton C.J. (2022). Strategies to prevent and manage running-related knee injuries: A systematic review of randomised controlled trials. Br. J. Sports Med..

[37] Avila-Quintero S.E., Suescún-Carrero S.H., González-Cetina N.F., Zapata-Gil S., Afanador D.F. (2024). Dose-response of eccentric training to prevent hamstring injuries in soccer players: A systematic review with meta-analysis. Retos.

[38] Crossley K.M., E Patterson B., Culvenor A.G., Bruder A.M., Mosler A.B., Mentiplay B.F. (2020). Making football safer for women: A systematic review and meta-analysis of injury prevention programmes in 11,773 female football (soccer) players. Br. J. Sports Med..

[39] Grimm N.L., Jacobs J.C., Kim J., Denney B.S., Shea K.G. (2015). Anterior Cruciate Ligament and Knee Injury Prevention Programs for Soccer Players: A Systematic Review and Meta-analysis. Am. J. Sports Med..

[40] Grimm N.L., Jacobs J.C., Kim J., Amendola A., Shea K.G. (2016). Ankle Injury Prevention Programs for Soccer Athletes Are Protective: A Level-I Meta-Analysis. J. Bone Jt. Surg. Am..

[41] Obërtinca R., Hoxha I., Meha R., Lama A., Bimbashi A., Kuqi D., Shabani B., Meyer T., der Fünten K.A. (2023). Efficacy of Multi-Component Exercise-Based Injury Prevention Programs on Injury Risk Among Footballers of All Age Groups: A Systematic Review and Meta-analysis. Sports Med..

[42] Ripley N.J., Cuthbert M., Ross S., Comfort P., McMahon J.J. (2021). The Effect of Exercise Compliance on Risk Reduction for Hamstring Strain Injury: A Systematic Review and Meta-Analyses. Int. J. Environ. Res. Public Health.

[43] Thorborg K., Krommes K.K., Esteve E., Clausen M.B., Bartels E.M., Rathleff M.S. (2017). Effect of specific exercise-based football injury prevention programmes on the overall injury rate in football: A systematic review and meta-analysis of the FIFA 11 and 11+ programmes. Br. J. Sports Med..

[44] Yang J., Wang Y., Chen J., Yang J., Li N., Wang C., Liao Y. (2022). Effects of the “FIFA11+ Kids” Program on Injury Prevention in Children: A Systematic Review and Meta-Analysis. Int. J. Environ. Res. Public Health.

[45] Carter C.W., Ireland M.L., Johnson A.E., Levine W.N., Martin S., Bedi A., Matzkin E.G. (2018). Sex-based Differences in Common Sports Injuries. J. Am. Acad. Orthop. Surg..

[46] Costa E Silva L., Teles J., Fragoso I. (2022). Sports injuries patterns in children and adolescents according to their sports participation level, age and maturation. BMC Sports Sci. Med. Rehabil..

[47] Slade S.C., Dionne C.E., Underwood M., Buchbinder R. (2016). Consensus on Exercise Reporting Template (CERT): Modified Delphi Study. Phys. Ther..

