# Supplementary material for: Protecting Athletes: The Clinical Relevance of Meta-Analyses on Injury Prevention Programs for Sports and Musculoskeletal Body Regions: An Overview of Systematic Reviews with Meta-Analyses of Randomized Clinical Trials
Source: Healthcare (Basel). 2025 Jun 27;13(13):1530. doi: 10.3390/healthcare13131530 (PMC12250077; doi:10.3390/healthcare13131530)
Supplement: Supplementary file 1 [file healthcare-13-01530-s001.zip › Suppl File S1 Deviations from the protocol.pdf]

Supplementary file S1. Deviations from the protocol.

- E-databases: We systematically searched seven out of eight e-databases that were stated in the protocol. Finally, PsycINFO was not consulted because the focus of this e-database was not related to the objective of this overview.
- Manual searches: We manually screened other syntheses of evidence rather than overviews of reviews and scoping reviews.
- Eligibility criteria (type of injury): Musculoskeletal injuries and reinjuries were eventually considered. Other sport-related injuries or reinjuries such as sport-related concussions were not considered. We truly believe that focusing the article only on musculoskeletal injuries and reinjuries has allowed us to draw more clinical and comparable conclusions.
- Eligibility criteria (outcome): We also considered the outcome incidence along with the risk of developing sport-related musculoskeletal injuries and reinjuries. We considered both allowed us to reach more firm conclusions about our objectives.
- Eligibility criteria (study design): We only included meta-analyses evaluating randomized controlled trials (including different forms of randomized clinical trials (cluster, pilot, etc.). We made this decision because randomized trials are considered the highest form of evidence.
- The degree of overlap between systematic reviews: We only considered the type of sport and musculoskeletal body region affected by injury as criteria to calculate the degree of overlap. We did not consider the type of intervention because most meta-analyses combined different protocols of intervention.
- Data extraction: some changes were made in data extraction. We only extracted specific information related to the population, study design, interventions, controls, meta-analyses of interest, and GRADE assessments. With this, we achieved simply the table.
- Results section: Subgroups by sex, age groups, or level of play were also shown in the results section. This allowed us to draw more clinical and comparable conclusions.
