# Supplementary material for: Protecting Athletes: The Clinical Relevance of Meta-Analyses on Injury Prevention Programs for Sports and Musculoskeletal Body Regions: An Overview of Systematic Reviews with Meta-Analyses of Randomized Clinical Trials
Source: Healthcare (Basel). 2025 Jun 27;13(13):1530. doi: 10.3390/healthcare13131530 (PMC12250077; doi:10.3390/healthcare13131530)
Supplement: Supplementary file 1 [file healthcare-13-01530-s001.zip › Suppl File S2 Search strategies.pdf]

Supplementary file S2. Search Strategies**.**

CINAHL (date 07/10/2024)

AB (athlete* OR player* OR sport*) AND TI (tear* OR rupture OR injury OR injuries OR reinjur* OR re-injur* OR recurr* OR contusion* OR laceration* OR sprain* OR tendo* OR tendin* OR strain* OR luxation* OR subluxation* OR sub-luxation* OR fracture* OR spasm* OR cramp* OR dislocation OR lesion* OR entrapment* OR derangement* OR trauma* OR avulsion OR damage) AND AB (rate* OR risk OR odds OR incidence OR ratio) AND AB (systematic OR meta-analysis OR metaanalysis OR meta-analyses OR metaanalyses OR meta-review OR meta-analytic-review OR overview-of-systematic OR overview-of-reviews OR umbrella-review OR scoping-review)

Search modes - Proximity.

Search filters

Publication type: no restrictions were imposed.

Language of publication: no restrictions were imposed.

Studies retrieved = 701

Embase (date 07/10/2024)

(athlete*:ab,ti OR player*:ab,ti OR sport*:ab,ti) AND (tear*:ti OR rupture:ti OR injury:ti OR injuries:ti OR reinjur*:ti OR re-injur*:ti OR recurr*:ti OR contusion*:ti OR laceration*:ti OR sprain*:ti OR tendo*:ti OR tendin*:ti OR strain*:ti OR luxation*:ti OR subluxation*:ti OR sub-luxation*:ti OR fracture*:ti OR spasm*:ti OR cramp*:ti OR dislocation:ti OR lesion*:ti OR entrapment*:ti OR derangement*:ti OR trauma*:ti OR avulsion:ti OR damage:ti) AND (rate*:ab,ti OR risk:ab,ti OR odds:ab,ti OR incidence:ab,ti OR ratio:ab,ti) AND (systematic:ab,ti OR meta-analysis:ab,ti OR metaanalysis:ab,ti OR meta-analyses:ab,ti OR metaanalyses:ab,ti OR meta-review:ab,ti OR meta-analytic-

review:ab,ti OR overview-of-systematic:ab,ti OR overview-of-reviews:ab,ti OR umbrella-review:ab,ti OR scoping-review:ab,ti)

Search filters

Publication type: conference abstract were not considered in search strategies.

Language of publication: no restrictions were imposed.

Studies retrieved = 1331

Epistemonikos (date 07/10/2024)

(advanced_title_en:((athlete* OR player* OR sport*)) OR advanced_abstract_en:((athlete* OR player* OR sport*))) AND advanced_title_en:((tear* OR rupture OR injury OR injuries OR reinjur* OR re-injur* OR recurr* OR contusion* OR laceration* OR sprain* OR tendo* OR tendin* OR strain* OR luxation* OR subluxation* OR sub-luxation* OR fracture* OR spasm* OR cramp* OR dislocation OR lesion* OR entrapment* OR derangement* OR trauma* OR avulsion OR damage)) AND (advanced_title_en:((rate* OR risk OR odds OR incidence OR ratio)) OR advanced_abstract_en:((rate* OR risk OR odds OR incidence OR ratio))) AND (advanced_title_en:((systematic OR meta-analysis OR metaanalysis OR meta-analyses OR metaanalyses OR meta-review OR meta-analytic-review OR overview-of-systematic OR overview-of-reviews OR umbrella-review OR scoping-review)) OR advanced_abstract_en:((systematic OR meta-analysis OR metaanalysis OR meta-analyses OR metaanalyses OR meta-review OR meta-analytic-review OR overview-of-systematic OR overview-of-reviews OR umbrella-review OR scoping-review)))

Search filters

Publication type: no restrictions were imposed.

Language of publication: no restrictions were imposed.

Studies retrieved = 1286

PubMed (date 07/10/2024)

(athlete* [tiab] OR player* [tiab] OR sport*[tiab]) AND (tear* [title] OR rupture [title] OR injury [title] OR injuries [title] OR reinjur* [title] OR re-injur* [title] OR recurr* [title] OR contusion* [title] OR laceration* [title] OR sprain* [title] OR tendo* [title] OR tendin* [title] OR strain* [title] OR luxation* [title] OR subluxation* [title] OR sub-luxation* [title] OR fracture* [title] OR spasm* [title] OR cramp* [title] OR dislocation [title] OR lesion* [title] OR entrapment* [title] OR derangement* [title] OR trauma* [title] OR avulsion [title] OR damage [title]) AND (rate* [tiab] OR risk [tiab] OR odds [tiab] OR incidence [tiab] OR ratio [tiab]) AND (systematic [tiab] OR meta-analysis [tiab] OR metaanalysis [tiab] OR meta-analyses [tiab] OR metaanalyses [tiab] OR meta-review [tiab] OR meta-analytic-review [tiab] OR overview-of-systematic [tiab] OR overview-of-reviews [tiab] OR umbrella-review [tiab] OR scoping-review [tiab])

Search filters

Publication type: no restrictions were imposed.

Language of publication: no restrictions were imposed.

Studies retrieved = 1503

Scopus (date 07/10/2024)

TITLE-ABS-KEY (athlete* OR player* OR sport*) AND TITLE (tear* OR rupture OR injury OR injuries OR reinjur* OR re-injur* OR recurr* OR contusion* OR laceration* OR sprain* OR tendo* OR tendin* OR strain* OR luxation* OR subluxation* OR sub-luxation* OR fracture* OR spasm* OR cramp* OR dislocation OR lesion* OR

entrapment* OR derangement* OR trauma* OR avulsion OR damage) AND TITLE-ABS-KEY (rate* OR risk OR odds OR incidence OR ratio) AND TITLE-ABS-KEY (systematic OR meta-analysis OR metaanalysis OR meta-analyses OR metaanalyses OR meta-review OR meta-analytic-review OR overview-of-systematic OR overview-of-reviews OR umbrella-review OR scoping-review)

Search filters

Publication type: no restrictions were imposed.

Language of publication: no restrictions were imposed.

Studies retrieved = 2,001

SPORTDiscus (date 07/10/2024)

AB (athlete* OR player* OR sport*) AND TI (tear* OR rupture OR injury OR injuries OR reinjur* OR re-injur* OR recurr* OR contusion* OR laceration* OR sprain* OR tendo* OR tendin* OR strain* OR luxation* OR subluxation* OR sub-luxation* OR fracture* OR spasm* OR cramp* OR dislocation OR lesion* OR entrapment* OR derangement* OR trauma* OR avulsion OR damage) AND AB (rate* OR risk OR odds OR incidence OR ratio) AND AB (systematic OR meta-analysis OR metaanalysis OR meta-analyses OR metaanalyses OR meta-review OR meta-analytic-review OR overview-of-systematic OR overview-of-reviews OR umbrella-review OR scoping-review)

Search modes - Proximity.

Search filters

Publication type: no restrictions were imposed.

Language of publication: no restrictions were imposed.

Studies retrieved = 610

The Cochrane Library (date 07/10/2024)

ID	Search	Hits
#1	(athlete*):ti,ab,kw	9917
#2	(player*):ti,ab,kw	5736
#3	(sport*):ti,ab,kw	15135
#4	(tear*):ti	2213
#5	(rupture):ti	1443
#6	(injury):ti	15972
#7	(injuries):ti	2707
#8	(reinjur*):ti	31
#9	(re-injur*):ti	24
#10	(recurr*):ti	19073
#11	(contusion*):ti	104
#12	(laceration*):ti	346
#13	(sprain*):ti	885
#14	(tendo*):ti	2153
#15	(tendin*):ti	1187
#16	(strain*):ti	2099

#17	(luxation*):ti	18
#18	(subluxation*):ti	148
#19	(sub-luxation*):ti	1
#20	(fracture*):ti	15123
#21	(spasm*):ti	955
#22	(cramp*):ti	408
#23	(dislocation):ti	601
#24	(lesion*):ti	8238
#25	(entrapment*):ti	69
#26	(derangement*):ti	127
#27	(trauma*):ti	11232
#28	(avulsion):ti	112
#29	(damage):ti	3499
#30	(rate*):ti,ab,kw	518206
#31	(risk):ti,ab,kw	313103
#32	(odds):ti,ab,kw	37585
#33	(incidence):ti,ab,kw	159884
#34	(ratio):ti,ab,kw	163990

#35 (#1 OR #2 OR #3) AND (#4 OR #5 OR #6 OR #7 OR #8 OR #9 OR #10 OR #11 OR #12 OR #13 OR #14 OR #15 OR #16 OR #17 OR #18 OR #19 OR #20 OR #21 OR #22 OR #23 OR #24 OR #25 OR #26 OR #27 OR #28 OR #29) AND (#30 OR #31 OR #32 OR #33 OR #34) 1210

Search filters

Type of document: Cochrane reviews and Cochrane protocols.

Language of publication: no restrictions were imposed.

Studies retrieved = 25 (25 Cochrane reviews and 0 Cochrane Protocols).
