# Supplementary material for: Protecting Athletes: The Clinical Relevance of Meta-Analyses on Injury Prevention Programs for Sports and Musculoskeletal Body Regions: An Overview of Systematic Reviews with Meta-Analyses of Randomized Clinical Trials
Source: Healthcare (Basel). 2025 Jun 27;13(13):1530. doi: 10.3390/healthcare13131530 (PMC12250077; doi:10.3390/healthcare13131530)
Supplement: Supplementary file 1 [file healthcare-13-01530-s001.zip › Suppl File S3 List excluded studies.pdf]

Supplementary file S3. Full text analysis: excluded studies and reasons for exclusion. Included (k=15)

No access at full text (k=5)

No direct comparison in meta-analyses were reported (k=1)

No intervention of interest (k=24)

No meta-analysis (k=135)

No meta-analysis of interest (k=67)

No objective of this study (k=7)

No outcome of interest (k=1)

No population of interest (k=3)

No research design of interest (k=27)

The focus of the study was not primarily on sports populations (k=31)

Study		Reason
1.	Aaltonen S, Karjalainen H, Heinonen A, Parkkari J, Kujala UM. Prevention of sports injuries: systematic review of randomized controlled trials. Arch Intern Med. 2007 Aug 13-27;167(15):1585-92. doi: 10.1001/archinte.167.15.1585.	No meta-analysis.
2.	Aarts D, Barendrecht M, Kemler E, Gouttebarge V. The prevention of injuries among youth basketballers according to the "Sequence of Prevention": a systematic review. SA J. Sports Med. 2021 [cited 2024 Oct 24] ; 33(1): 1-12.	No meta-analysis.
3.	Abdur-Rahman LO, van As AB, Rode H. Pediatric trauma care in Africa: the evolution and challenges. Semin Pediatr Surg. 2012 May;21(2):111-5. doi: 10.1053/j.sempedsurg.2012.01.003.	No meta-analysis.
4.	Afonso J, Olivares-Jabalera J, Fernandes RJ, Clemente FM, Rocha-Rodrigues S, Claudino JG, Ramirez-Campillo R, Valente C, Andrade R, Espregueira-Mendes J. Effectiveness of Conservative Interventions After Acute Hamstrings Injuries in Athletes: A Living Systematic Review. Sports Med. 2023 Mar;53(3):615-635. doi: 10.1007/s40279-022-01783-z.	No meta-analysis.
5.	Al Attar WS, Soomro N, Pappas E, Sinclair PJ, Sanders RH. How Effective are F-MARC Injury Prevention Programs for Soccer Players? A Systematic Review and Meta-Analysis. Sports Med. 2016 Feb;46(2):205-17. doi: 10.1007/s40279-015-0404-x.	No meta-analysis of interest.<br><br>Note: This review included non-randomized studies and subgroups by research design were not performed.
6.	Al Attar WSA, Soomro N, Sinclair PJ, Pappas E, Sanders RH. Effect of Injury Prevention Programs that Include the Nordic Hamstring Exercise on Hamstring Injury Rates in Soccer Players: A Systematic Review and Meta-Analysis. Sports Med. 2017 May;47(5):907-916. doi: 10.1007/s40279-016-0638-2.	No meta-analysis of interest.

	Note: This review included non-randomized studies and subgroups by research design were not performed.
7. Al Attar WSA, Alshehri MA. A meta-analysis of meta-analyses of the effectiveness of FIFA injury prevention programs in soccer. Scand J Med Sci Sports. 2019 Dec;29(12):1846-1855. doi: 10.1111/sms.13535.	No research design of interest.
8. Al Attar WSA, Khaledi EH, Bakhsh JM, Faude O, Ghulam H, Sanders RH. Injury prevention programs that include balance training exercises reduce ankle injury rates among soccer players: a systematic review. J Physiother. 2022 Jul;68(3):165-173. doi: 10.1016/j.jphys.2022.05.019.	Included.
9. Al Attar WSA, Bakhsh JM, Khaledi EH, Ghulam H, Sanders RH. Injury prevention programs that include plyometric exercises reduce the incidence of anterior cruciate ligament injury: a systematic review of cluster randomised trials. J Physiother. 2022 Oct;68(4):255-261. doi: 10.1016/j.jphys.2022.09.001.	No meta-analysis of interest.<br><br>Note: Meta-analyses were not performed by type of sport.
10. Al Attar et al. The Effectiveness of Injury Prevention Programs That Include Core Stability Exercises in Reducing the Incidence of Knee Injury Among Soccer Players: A Systematic Review and Meta-analysis. Isokinetics and Exercise Science. 2022;30(4): 281-291.	Included.
11. Al Attar WA, Ghulam H, Al Arifi S, Alomar AI, Alhosaini S, Alharbi S, Alraddadi Y, Sanders RH. Injury prevention programs including balance exercises with compliance and follow-up reduce the incidence of knee injuries in athletes: A systematic review and meta-analysis. Isokinetics and Exercise Science. 2023;31(3):157-169.	Included.
12. Al Attar WSA, Husain MA. Effectiveness of Injury Prevention Programs With Core Muscle Strengthening Exercises to Reduce the Incidence of Hamstring Injury Among Soccer Players: A Systematic Review and Meta-Analysis. Sports Health. 2023 Nov-Dec;15(6):805-813. doi: 10.1177/19417381231170815.	Included.
13. Alcelik I, Saeed ZM, Haughton BA, Shahid R, Alcelik JC, Brogden C, Budgen A. Achillon versus open surgery in acute Achilles tendon repair. Foot Ankle Surg. 2018 Oct;24(5):427-434. doi: 10.1016/j.fas.2017.04.016.	No intervention of interest.
14. Alentorn-Geli E, Mendiguchía J, Samuelsson K, Musahl V, Karlsson J, Cugat R, Myer GD. Prevention of non-contact anterior cruciate ligament injuries in sports. Part II: systematic review of the effectiveness of prevention programmes in male athletes. Knee Surg Sports Traumatol Arthrosc. 2014 Jan;22(1):16-25. doi: 10.1007/s00167-013-2739-x.	No meta-analysis.
15. Alexander JLN, Culvenor AG, Johnston RRT, Ezzat AM, Barton CJ. Strategies to prevent and manage running-related knee injuries: a systematic review of randomised controlled trials. Br J Sports Med. 2022 Nov;56(22):1307-1319. doi: 10.1136/bjsports-2022-105553.	Included.
16. Alkhatib N, Abdullah ASA, AlNouri M, Ahmad Alzobi OZ, Alkaramany E, Ishibashi Y. Short- and long-term outcomes in Bankart repair vs. conservative treatment for first-time anterior shoulder dislocation: a systematic review and meta-analysis of randomized controlled trials. J Shoulder Elbow Surg. 2022 Aug;31(8):1751-1762. doi: 10.1016/j.jse.2022.02.032.	The focus of the study was not primarily on sports populations.
17. Althomali OW, Ibrahim AA, Algharbi AF, Alshammari SS, Alajlan SN, Albaqawi JA, Alshammari AF, Sheeha BB, Hussein HM. The FIFA 11+ injury prevention program	No access at full text.

	reduces the incidence of lower extremity injuries in football players: a systematic review and meta-analysis. J Sports Med Phys Fitness. 2024 Oct 3. doi: 10.23736/S0022-4707.24.15910-5.	Note: This study was request to the original authors, but an answer was not received.
18.	Andrew N, Gabbe BJ, Cook J, Lloyd DG, Donnelly CJ, Nash C, Finch CF. Could targeted exercise programmes prevent lower limb injury in community Australian football? Sports Med. 2013 Aug;43(8):751-63. doi: 10.1007/s40279-013-0056-7.	No meta-analysis.
19.	Antoranz Y, Sáez de Villarreal E, Del Campo Vecino J, Jiménez-Saiz SL. Sure Steps: Key Strategies for Protecting Basketball Players from Injuries-A Systematic Review. J Clin Med. 2024 Aug 20;13(16):4912. doi: 10.3390/jcm13164912.	No meta-analysis.
20.	Arnold A, Thigpen CA, Beattie PF, Kissenberth MJ, Shanley E. Overuse Physeal Injuries in Youth Athletes. Sports Health. 2017 Mar/Apr;9(2):139-147. doi: 10.1177/1941738117690847.	No meta-analysis.
21.	Asker M, Brooke HL, Waldén M, Tranaeus U, Johansson F, Skillgate E, Holm LW. Risk factors for, and prevention of, shoulder injuries in overhead sports: a systematic review with best-evidence synthesis. Br J Sports Med. 2018 Oct;52(20):1312-1319. doi: 10.1136/bjsports-2017-098254.	No meta-analysis.
22.	Atanda A Jr, Reddy D, Rice JA, Terry MA. Injuries and chronic conditions of the knee in young athletes. Pediatr Rev. 2009 Nov;30(11):419-28; quiz 429-30. doi: 10.1542/pir.30-11-419.	No objective of this study.
23.	Avila-Quintero SE, Suescún-Carrero SH, González-Cetina NF, Zapata-Gil S, Afanador DF. Dosis-respuesta del entrenamiento excéntrico para prevenir lesiones en isquiotibiales en futbolistas una revisión sistemática con metaanálisis. Retos. 2024;57:8-17.	Included.
24.	Ayala F, Robles-Palazón FJ, Blázquez-Rincón D, López-Valenciano A, López-López JA, De Ste Croix M. A systematic review and network meta-analysis on the effectiveness of exercise-based interventions for reducing the injury incidence in youth team-sport players. Part 2: an analysis by movement patterns. Ann Med. 2024 Dec;56(1):2337724. doi: 10.1080/07853890.2024.2337724.	No meta-analysis of interest.<br><br>Note: No overall meta-analyses or specific subgroups including all our inclusion criteria were found.
25.	Azevedo KP, Bastos JAI, de Sousa Neto IV, Pastre CM, Durigan JLQ. Different Cryotherapy Modalities Demonstrate Similar Effects on Muscle Performance, Soreness, and Damage in Healthy Individuals and Athletes: A Systematic Review with Metanalysis. J Clin Med. 2022 Jul 30;11(15):4441. doi: 10.3390/jcm11154441.	No intervention of interest.
26.	Azhar NI, Othaman NN, Zainuddin SZ, Justine M, Munajat M, Kamarulzaman MF, Bukry SA. FIFA 11+ Prevention Programme in Preventing Anterior Cruciate Ligament Injury among Soccer Players: A Scoping Review. Mal J Med Health Sci. 2022;18(SUPP8):374-385.	No research design of interest.
27.	Azócar-Gallardo J, Azócar-Gallardo Y, Ojeda-Aravena A, Cárdenas-Mansill R, Montecinos-Zuñiga J. Efectividad del entrenamiento de la musculatura del Core en la prevención de lesiones de hombro en deportes con lanzamientos sobre la cabeza: una revisión sistemática. Rev Andal Med Deporte. 2021;14(2):120-124.	No research design of interest.
28.	Barelds I, van den Broek AG, Huisstede BMA. Ankle Bracing is Effective for Primary and Secondary Prevention of Acute Ankle Injuries in Athletes: A Systematic Review and Meta-Analyses. Sports Med. 2018 Dec;48(12):2775-2784. doi: 10.1007/s40279-018-0993-2.	No intervention of interest.
29.	Barengo NC, Meneses-Echávez JF, Ramírez-Vélez R, Cohen DD, Tovar G, Bautista JE. The impact of the FIFA 11+ training program on injury prevention in football	No meta-analysis.

	players: a systematic review. Int J Environ Res Public Health. 2014 Nov 19;11(11):11986-2000. doi: 10.3390/ijerph111111986.	
30.	Barrera J, Figueiredo AJ, Clemente FM, Field A, Valenzuela L, Sarmento H. Injury Prevention Programmes in Male Soccer Players: An Umbrella Review of Systematic Reviews. Journal of Men's Health. 2022;18(10):1-17.	No research design of interest.
31.	Bellows R, Wong CK. The effect of bracing and balance training on ankle sprain incidence among athletes: a systematic review with meta-analysis. Int J Sports Phys Ther. 2018 Jun;13(3):379-388.	No meta-analysis of interest.<br><br>Note: Meta-analyses were not performed by type of sport.
32.	Berkey R, Sunesara A, Allen L, Pontiff R, DeVries A, Fisher SR. Ankle Injury Prevention Programs for Youth Sports: A Systematic Review and Meta-analysis. Sports Health. 2024 Feb 26:19417381241231588. doi: 10.1177/19417381241231588.	No meta-analysis of interest.<br><br>Note: Meta-analyses were not performed by type of sport.
33.	Bieuzen F, Bleakley CM, Costello JT. Contrast water therapy and exercise induced muscle damage: a systematic review and meta-analysis. PLoS One. 2013 Apr 23;8(4):e62356. doi: 10.1371/journal.pone.0062356.	No intervention of interest.
34.	Bigdon SF, Hecht V, Fairhurst PG, Deml MC, Exadaktylos AK, Albers CE. Injuries in alpine summer sports - types, frequency and prevention: a systematic review. BMC Sports Sci Med Rehabil. 2022 May 1;14(1):79. doi: 10.1186/s13102-022-00468-4.	No meta-analysis.
35.	Biz C, Nicoletti P, Baldin G, Bragazzi NL, Crimì A, Ruggieri P. Hamstring Strain Injury (HSI) Prevention in Professional and Semi-Professional Football Teams: A Systematic Review and Meta-Analysis. Int J Environ Res Public Health. 2021 Aug 4;18(16):8272. doi: 10.3390/ijerph18168272.	No meta-analysis of interest.<br><br>Note: This review included non-randomized studies and subgroups by research design were not performed.
36.	Blasimann A, Eberle S, Scuderi MM. Effekt eines Rumpfkräftigungsprogramms (inklusive Unterarm- und seitlichem Unterarmstütz) auf die Verletzungsrate von erwachsenen Fußballspielern: eine systematische Literaturübersicht [Effect of Core Muscle Strengthening Exercises (Including Plank and Side Plank) on Injury Rate in Male Adult Soccer Players: A Systematic Review]. Sportverletz Sportschaden. 2018 Mar;32(1):35-46. German. doi: 10.1055/a-0575-2324.	No meta-analysis.
37.	Bodendorfer BM, McCormick BP, Wang DX, Looney AM, Conroy CM, Fryar CM, Kotler JA, Ferris WJ, Postma WF, Chang ES. Treatment of Pectoralis Major Tendon Tears: A Systematic Review and Meta-analysis of Operative and Nonoperative Treatment. Orthop J Sports Med. 2020 Feb 6;8(2):2325967119900813. doi: 10.1177/2325967119900813.	The focus of the study was not primarily on sports populations.
38.	Bonilla DA, Cardozo LA, Vélez-Gutiérrez JM, Arévalo-Rodríguez A, Vargas-Molina S, Stout JR, Kreider RB, Petro JL. Exercise Selection and Common Injuries in Fitness Centers: A Systematic Integrative Review and Practical Recommendations. Int J Environ Res Public Health. 2022 Oct 5;19(19):12710. doi: 10.3390/ijerph191912710.	No meta-analysis.

39. Bogunovic L, Tarabichi M, Harris D, Wright R. Treatment of tibial eminence fractures: a systematic review. J Knee Surg. 2015 Jun;28(3):255-62. doi: 10.1055/s-0034-1388657.	The focus of the study was not primarily on sports populations.
40. Bogwasi L, Holtzhausen L, Janse van Rensburg DC, Jansen van Rensburg A, Botha T. Management of proximal rectus femoris injuries - do we know what we're doing?: A systematic review. Biol Sport. 2023 Apr;40(2):497-512. doi: 10.5114/biolsport.2023.116454.	No meta-analysis.
41. Bonell Monsonís O, Spörri J, Warsen M, Bolling C, Gouttebarge V, Verhagen E. We know a lot about little and little about a lot: A contextualized scoping review on injury prevention in alpine ski racing. Scand J Med Sci Sports. 2024 Jan;34(1):e14533. doi: 10.1111/sms.14533.	No research design of interest.
42. Box MW, Wilson F, Pasque CB, Smith CD. Characteristics of Rodeo Injuries and Suggestions for Injury Prevention: A Systematic Review. Orthop J Sports Med. 2024 Apr 15;12(4):23259671241227217. doi: 10.1177/23259671241227217.	No meta-analysis.
43. Braun C, McRobert CJ. Conservative management following closed reduction of traumatic anterior dislocation of the shoulder. Cochrane Database Syst Rev. 2019 May 10;5(5):CD004962. doi: 10.1002/14651858.CD004962.pub4.	The focus of the study was not primarily on sports populations.
44. Briggs MS, Givens DL, Best TM, Chaudhari AM. Lumbopelvic neuromuscular training and injury rehabilitation: a systematic review. Clin J Sport Med. 2013 May;23(3):160-71. doi: 10.1097/JSM.0b013e318280aabb.	No meta-analysis.
45. Brumann M, Baumbach SF, Mutschler W, Polzer H. Accelerated rehabilitation following Achilles tendon repair after acute rupture - Development of an evidence-based treatment protocol. Injury. 2014 Nov;45(11):1782-90. doi: 10.1016/j.injury.2014.06.022.	No meta-analysis.
46. Brunner R, Friesenbichler B, Casartelli NC, Bizzini M, Maffiuletti NA, Niedermann K. Effectiveness of multicomponent lower extremity injury prevention programmes in team-sport athletes: an umbrella review. Br J Sports Med. 2019 Mar;53(5):282-288. doi: 10.1136/bjsports-2017-098944.	No research design of interest.
47. Burger M, Dreyer D, Fisher RL, Foot D, O'Connor DH, Galante M, Zalgaonkir S. The effectiveness of proprioceptive and neuromuscular training compared to bracing in reducing the recurrence rate of ankle sprains in athletes: A systematic review and meta-analysis. J Back Musculoskelet Rehabil. 2018;31(2):221-229. doi: 10.3233/BMR-170804.	No meta-analysis of interest.<br><br>Note: Meta-analyses were not performed by type of sport.
48. Burton I. Interventions for prevention and in-season management of patellar tendinopathy in athletes: A scoping review. Phys Ther Sport. 2022 May;55:80-89. doi: 10.1016/j.ptsp.2022.03.002.	No research design of interest.
49. Cadens M, Planas A, Matas S, Peirau X. Preventive Training of Anterior Cruciate Ligament Injuries in Female Handball Players: a Systematic Review. Apunts Educación Física y Deportes. 2021;146:68-77. https://doi.org/10.5672/apunts.2014-0983.es.(2021/4).146.08	No meta-analysis.
50. Caldemeyer LE, Brown SM, Mulcahey MK. Neuromuscular training for the prevention of ankle sprains in female athletes: a systematic review. Phys Sportsmed. 2020 Nov;48(4):363-369. doi: 10.1080/00913847.2020.1732246.	No meta-analysis.
51. Charlton PC, Drew MK, Mentiplay BF, Grimaldi A, Clark RA. Exercise Interventions for the Prevention and Treatment of Groin Pain and Injury in Athletes: A Critical and Systematic Review. Sports Med. 2017 Oct;47(10):2011-2026. doi: 10.1007/s40279-017-0742-y.	No meta-analysis.

52.	Chavarro-Nieto C, Beaven M, Gill N, Hébert-Losier K. Hamstrings injury incidence, risk factors, and prevention in Rugby Union players: a systematic review. Phys Sportsmed. 2023 Feb;51(1):1-19. doi: 10.1080/00913847.2021.1992601.	No meta-analysis.
53.	Chen J, Zhang C, Chen S, Zhao Y. Effects of functional correction training on injury risk of athletes: a systematic review and meta-analysis. PeerJ. 2021 Mar 25;9:e11089. doi: 10.7717/peerj.11089.	No meta-analysis of interest.<br><br>Note: Meta-analyses were not performed by type of sport.
54.	Chiddarwar V, de Zoete RMJ, Dickson C, Lathlean T. Effectiveness of combined surgical and exercise-based interventions following primary traumatic anterior shoulder dislocation: a systematic review and meta-analysis. Br J Sports Med. 2023 Dec;57(23):1498-1508. doi: 10.1136/bjsports-2022-106422.	The focus of the study was not primarily on sports populations. Note: This review included athletic populations, but no specific analyses for athletes were conducted.
55.	Chona DV, Minetos PD, LaPrade CM, Cinque ME, Abrams GD, Sherman SL, Safran MR. Hip Dislocation and Subluxation in Athletes: A Systematic Review. Am J Sports Med. 2022 Aug;50(10):2834-2841. doi: 10.1177/03635465211036104.	No intervention of interest.
56.	Ciccotti MC, Secrist E, Tjoumakaris F, Ciccotti MG, Freedman KB. Anatomic Anterior Cruciate Ligament Reconstruction via Independent Tunnel Drilling: A Systematic Review of Randomized Controlled Trials Comparing Patellar Tendon and Hamstring Autografts. Arthroscopy. 2017 May;33(5):1062-1071.e5. doi: 10.1016/j.arthro.2017.01.033.	No intervention of interest.
57.	Coopmans L, Amaya Aliaga J, Metsemakers WJ, Sermon A, Misselyn D, Nijs S, Hoekstra H. Accelerated Rehabilitation in Non-operative Management of Acute Achilles Tendon Ruptures: A Systematic Review and Meta-analysis. J Foot Ankle Surg. 2022 Jan-Feb;61(1):157-162. doi: 10.1053/j.jfas.2021.07.007.	The focus of the study was not primarily on sports populations.
58.	Cornelissen M, Kemler E, Verhagen E, Gouttebarge V. A systematic review of injuries in recreational field hockey: From injury problem to prevention. J Sports Sci. 2020 Sep;38(17):1953-1974. doi: 10.1080/02640414.2020.1764898.	No meta-analysis.
59.	Coves-García A, Lozano-Quijada C, Poveda-Pagán EJ. Estrategias para la prevención de lesiones de ligamento cruzado anterior en mujeres deportistas con valgo dinámico de rodilla. Revisión sistemática. Fisioterapia. 2023;45(5):273-289.	No meta-analysis.
60.	Crawford AE, Picken LK, Gabriel FD, Quade J, Gould S. CNS and Thorax Injury and Associated Risks Factors in Equestrian Sports. Sports Health. 2024 Aug 29:19417381241275655. doi: 10.1177/19417381241275655.	No intervention of interest.
61.	Crossley KM, Patterson BE, Culvenor AG, Bruder AM, Mosler AB, Mentiplay BF. Making football safer for women: a systematic review and meta-analysis of injury prevention programmes in 11 773 female football (soccer) players. Br J Sports Med. 2020 Sep;54(18):1089-1098. doi: 10.1136/bjsports-2019-101587.	Included.
62.	Cruz-Ferreira A, Marujo A, Folgao H, Gutierres Filho P, Fernandes J. Programas de exercício na prevenção de lesões em jogadores de futebol: uma revisão sistemática. Rev Bras Med Esporte. 2015;21(3):236-241.	No meta-analysis.

63. Cusimano MD, Nastis S, Zuccaro L. Effectiveness of interventions to reduce aggression and injuries among ice hockey players: a systematic review. CMAJ. 2013 Jan 8;185(1):E57-69. doi: 10.1503/cmaj.112017.	No meta-analysis.
64. D'Ailly PN, Sluiter JK, Kuijer PP. Rib stress fractures among rowers: a systematic review on return to sports, risk factors and prevention. J Sports Med Phys Fitness. 2016 Jun;56(6):744-53.	No meta-analysis.
65. Dahduli OS, AlHossan AM, Al Rushud MA, Alneghaimshi MM, Alotaibi SF, AlNour MK, Al Otaibi AH, AlAseeri A, AlBatati S. Early Surgical Reconstruction Versus Rehabilitation for Patients With Anterior Cruciate Ligament Rupture: A Systematic Review and Meta-Analysis. Cureus. 2023 Aug 12;15(8):e43370. doi: 10.7759/cureus.43370.	The focus of the study was not primarily on sports populations.
66. Dai W, Leng X, Wang J, Hu X, Ao Y. Rehabilitation regimen for non-surgical treatment of Achilles tendon rupture: A systematic review and meta-analysis of randomised controlled trials. J Sci Med Sport. 2021 Jun;24(6):536-543. doi: 10.1016/j.jsams.2020.12.005.	The focus of the study was not primarily on sports populations.
67. Daly E, Pearce AJ, Ryan L. A Systematic Review of Strength and Conditioning Protocols for Improving Neck Strength and Reducing Concussion Incidence and Impact Injury Risk in Collision Sports; Is There Evidence? J Funct Morphol Kinesiol. 2021 Jan 12;6(1):8. doi: 10.3390/jfmk6010008.	No meta-analysis.
68. Dang Y, Chen R, Koutedakis Y, Wyon MA. The Efficacy of Physical Fitness Training on Dance Injury: A Systematic Review. Sportverletz Sportschaden. 2024 Aug;38(3):129-139. doi: 10.1055/a-2305-5759.	No meta-analysis.
69. Dargo L, Robinson KJ, Games KE. Prevention of Knee and Anterior Cruciate Ligament Injuries Through the Use of Neuromuscular and Proprioceptive Training: An Evidence-Based Review. J Athl Train. 2017 Dec;52(12):1171-1172. doi: 10.4085/1062-6050-52.12.21.	No research design of interest.
70. Davis AC, Emptage NP, Pounds D, Woo D, Sallis R, Romero MG, Sharp AL. The Effectiveness of Neuromuscular Warmups for Lower Extremity Injury Prevention in Basketball: A Systematic Review. Sports Med Open. 2021 Sep 16;7(1):67. doi: 10.1186/s40798-021-00355-1.	No meta-analysis.
71. de Queiros VS, Dos Santos ÍK, Almeida-Neto PF, Dantas M, de França IM, Vieira WHB, Neto GR, Dantas PMS, Cabral BGAT. Effect of resistance training with blood flow restriction on muscle damage markers in adults: A systematic review. PLoS One. 2021 Jun 18;16(6):e0253521. doi: 10.1371/journal.pone.0253521.	No meta-analysis.
72. de Souto Barreto P, Rolland Y, Vellas B, Maltais M. Association of Long-term Exercise Training With Risk of Falls, Fractures, Hospitalizations, and Mortality in Older Adults: A Systematic Review and Meta-analysis. JAMA Intern Med. 2019 Mar 1;179(3):394-405. doi: 10.1001/jamainternmed.2018.5406.	No population of interest.
73. de Souto Barreto P, Maltais M, Rosendahl E, Vellas B, Bourdel-Marchasson I, Lamb SE, Pitkala K, Rolland Y. Exercise Effects on Falls, Fractures, Hospitalizations, and Mortality in Older Adults With Dementia: An Individual-Level Patient Data Meta-analysis. J Gerontol A Biol Sci Med Sci. 2021 Aug 13;76(9):e203-e212. doi: 10.1093/gerona/glaa307.	The focus of the study was not primarily on sports populations.
74. de Vasconcelos GS, Cini A, Sbruzzi G, Lima CS. Effects of proprioceptive training on the incidence of ankle sprain in athletes: systematic review and meta-analysis. Clin Rehabil. 2018 Dec;32(12):1581-1590. doi: 10.1177/0269215518788683.	No meta-analysis of interest.<br><br>Note: Meta-analyses were not performed by type of sport.

75. DeJong AF, Hertel J. Gait-training devices in the treatment of lower extremity injuries in sports medicine: current status and future prospects. Expert Rev Med Devices. 2018 Dec;15(12):891-909. doi: 10.1080/17434440.2018.1551130.	No outcome of interest.
76. Delgado PJ. Roturas del tendón distal del biceps braquial. Trauma. 2008;19(4):242-250.	No meta-analysis.
77. Delincé P, Ghafil D. Anterior cruciate ligament tears: conservative or surgical treatment? A critical review of the literature. Knee Surg Sports Traumatol Arthrosc. 2012 Jan;20(1):48-61. doi: 10.1007/s00167-011-1614-x.	No meta-analysis.
78. Deng S, Sun Z, Zhang C, Chen G, Li J. Surgical Treatment Versus Conservative Management for Acute Achilles Tendon Rupture: A Systematic Review and Meta-Analysis of Randomized Controlled Trials. J Foot Ankle Surg. 2017 Nov-Dec;56(6):1236-1243. doi: 10.1053/j.jfas.2017.05.036.	The focus of the study was not primarily on sports populations.
79. Ding L, Luo J, Smith DM, Mackey M, Fu H, Davis M, Hu Y. Effectiveness of Warm-Up Intervention Programs to Prevent Sports Injuries among Children and Adolescents: A Systematic Review and Meta-Analysis. Int J Environ Res Public Health. 2022 May 23;19(10):6336. doi: 10.3390/ijerph19106336.	No meta-analysis of interest.<br><br>Note: Meta-analyses were not performed by type of sport.
80. Dizon JM, Reyes JJ. A systematic review on the effectiveness of external ankle supports in the prevention of inversion ankle sprains among elite and recreational players. J Sci Med Sport. 2010 May;13(3):309-17. doi: 10.1016/j.jsams.2009.05.002.	No intervention of interest.
81. Donnelly RR, Ugbolue UC, Gao Y, Gu Y, Dutheil F, Baker JS. A Systematic Review and Meta-Analysis Investigating Head Trauma in Boxing. Clin J Sport Med. 2023 Nov 1;33(6):658-674. doi: 10.1097/JSM.0000000000001195.	No intervention of interest.
82. Dos Santos FS, de Oliveira Aguilar EC, Simoes da Costa BL. Prevenção de lesão em LCA em atletas profissionais no futebol: uma revisão sistemática. Revista Intercontinental de Gestão Desportiva. 2023;13(3):1-12.	No meta-analysis.
83. Doyle E, Doyle TLA, Bonacci J, Fuller JT. The Effectiveness of Gait Retraining on Running Kinematics, Kinetics, Performance, Pain, and Injury in Distance Runners: A Systematic Review With Meta-analysis. J Orthop Sports Phys Ther. 2022 Apr;52(4):192-A5. doi: 10.2519/jospt.2022.10585.	No meta-analysis of interest.<br>Note: The outcome injury was not meta-analyzed.
84. Ehlert A, Wilson PB. A Systematic Review of Golf Warm-ups: Behaviors, Injury, and Performance. J Strength Cond Res. 2019 Dec;33(12):3444-3462. doi: 10.1519/JSC.0000000000003329.	No meta-analysis.
85. El-Akkawi AI, Joanroy R, Barfod KW, Kallemose T, Kristensen SS, Viberg B. Effect of Early Versus Late Weightbearing in Conservatively Treated Acute Achilles Tendon Rupture: A Meta-Analysis. J Foot Ankle Surg. 2018 Mar-Apr;57(2):346-352. doi: 10.1053/j.jfas.2017.06.006.	The focus of the study was not primarily on sports populations.
86. Elliott J, Heron N, Versteegh T, Gilchrist IA, Webb M, Archbold P, Hart ND, Peek K. Injury Reduction Programs for Reducing the Incidence of Sport-Related Head and Neck Injuries Including Concussion: A Systematic Review. Sports Med. 2021 Nov;51(11):2373-2388. doi: 10.1007/s40279-021-01501-1.	No meta-analysis.
87. Emery CA. Injury prevention and future research. Med Sport Sci. 2005;49:170-191. doi: 10.1159/000085396.	No meta-analysis.
88. Emery CA, Roy TO, Whittaker JL, Nettel-Aguirre A, van Mechelen W. Neuromuscular training injury prevention strategies in youth sport: a systematic review and meta-analysis. Br J Sports Med. 2015 Jul;49(13):865-70. doi: 10.1136/bjsports-2015-094639.	No meta-analysis of interest.<br><br>Note: Meta-analyses were

		not performed by type of sport.
89.	Ernst E, Posadzki P. Chiropractic for the prevention and/or treatment of sports injuries: a systematic review of controlled clinical trials. Focus on Alternative and Complementary Therapies. 2011;17(1):9-14. https://doi.org/10.1111/j.2042-7166.2011.01111.x	No meta-analysis.
90.	Esteve E, Rathleff MS, Bagur-Calafat C, Urrútia G, Thorborg K. Prevention of groin injuries in sports: a systematic review with meta-analysis of randomised controlled trials. Br J Sports Med. 2015 Jun;49(12):785-91. doi: 10.1136/bjsports-2014-094162.	No meta-analysis of interest.<br><br>Note: No overall meta-analyses or specific subgroups including all our inclusion criteria were found.
91.	Eunbin L. Comparative study of sports-related injury prevention strategies across different disciplines.Revista Internacional de Medicina y Ciencias de la Actividad Física y el Deporte. 2024;24(96):103-119. https://doi.org/10.15366/rimcafd2024.96.007	No meta-analysis.
92.	Fagher K, Lexell J. Sports-related injuries in athletes with disabilities. Scand J Med Sci Sports. 2014 Oct;24(5):e320-31. doi: 10.1111/sms.12175.	No meta-analysis.
93.	Faizullin I, Faizullina E. Effects of balance training on post-sprained ankle joint instability. Int J Risk Saf Med. 2015;27 Suppl 1:S99-S101. doi: 10.3233/JRS-150707.	No meta-analysis.
94.	Fan Z, Min L, He W, Yang Y, Ma W, Yao J. Efficacy of multicomponent interventions on injury risk among ice and snow sports participants-a systematic review and meta-analysis. BMC Sports Sci Med Rehabil. 2024 Jun 18;16(1):135. doi: 10.1186/s13102-024-00921-6.	No intervention of interest.
95.	Fanchini M, Steendahl IB, Impellizzeri FM, Pruna R, Dupont G, Coutts AJ, Meyer T, McCall A. Exercise-Based Strategies to Prevent Muscle Injury in Elite Footballers: A Systematic Review and Best Evidence Synthesis. Sports Med. 2020 Sep;50(9):1653-1666. doi: 10.1007/s40279-020-01282-z.	No meta-analysis.
96.	Farrell SG, Hatem M, Bharam S. Acute Adductor Muscle Injury: A Systematic Review on Diagnostic Imaging, Treatment, and Prevention. Am J Sports Med. 2023 Nov;51(13):3591-3603. doi: 10.1177/03635465221140923.	No meta-analysis.
97.	Fernandes AA, da Silva CD, da Costa IT, Marins JCB. The "FIFA 11+" warm-up programme for preventing injuries in soccer players: a systematic review. Fisioter Mov Curitiba. 2015;28(2):397-405.	No meta-analysis.
98.	Fradkin AJ, Gabbe BJ, Cameron PA. Does warming up prevent injury in sport? The evidence from randomised controlled trials? J Sci Med Sport. 2006 Jun;9(3):214-20. doi: 10.1016/j.jsams.2006.03.026.	No meta-analysis.
99.	Frisch A, Croisier JL, Urhausen A, Seil R, Theisen D. Injuries, risk factors and prevention initiatives in youth sport. Br Med Bull. 2009;92:95-121. doi: 10.1093/bmb/ldp034.	No meta-analysis.
100.	Gagnier JJ, Morgenstern H, Chess L. Interventions designed to prevent anterior cruciate ligament injuries in adolescents and adults: a systematic review and meta-analysis. Am J Sports Med. 2013 Aug;41(8):1952-62. doi: 10.1177/0363546512458227. Epub 2012 Sep 12. Erratum in: Am J Sports Med. 2014 Jan;42(1):NP14.	No meta-analysis of interest.<br><br>Note: This review included non-randomized studies. Subgroup analysis by research design

	was conducted, but this subgroup included different types of sports.
101. Gaunt T, Maffulli N. Soothing suffering swimmers: a systematic review of the epidemiology, diagnosis, treatment and rehabilitation of musculoskeletal injuries in competitive swimmers. Br Med Bull. 2012 Sep;103(1):45-88. doi: 10.1093/bmb/ldr039.	No meta-analysis.
102. Geldenhuys AG, Burgess T, Roche S, Hendricks S. Return to play protocols for musculoskeletal upper and lower limb injuries in tackle-collision team sports: A systematic review. Eur J Sport Sci. 2022 Nov;22(11):1743-1756. doi: 10.1080/17461391.2021.1960623.	No meta-analysis.
103. Gene-Morales J, Saez-Berlanga A, Bermudez M, Flández J, Fritz N, Colado JC. Incidence and prevalence of injuries in futsal: A systematic review of the literature. Journal of Human Sport & Exercise. 2021;16:S1467-S1480.	No meta-analysis.
104. George ERM, Sheerin KR, Reid D. Criteria and Guidelines for Returning to Running Following a Tibial Bone Stress Injury: A Scoping Review. Sports Med. 2024 Sep;54(9):2247-2265. doi: 10.1007/s40279-024-02051-y.	No research design of interest.
105. Ghaddaf AA, Alomari MS, Alsharef JF, Alakkas E, Alshehri MS. Early versus late weightbearing in conservative management of acute achilles tendon rupture: A systematic review and meta-analysis of randomized controlled trials. Injury. 2022 Apr;53(4):1543-1551. doi: 10.1016/j.injury.2022.01.028.	The focus of the study was not primarily on sports populations.
106. Gibson ES, Cairo A, Räisänen AM, Kuntze C, Emery CA, Pasanen K. The Epidemiology of Youth Sport-Related Shoulder Injuries: A Systematic Review. Transl Sports Med. 2022 Aug 23;2022:8791398. doi: 10.1155/2022/8791398.	No meta-analysis.
107. Goldman EF, Jones DE. Interventions for preventing hamstring injuries. Cochrane Database Syst Rev. 2010 Jan 20;(1):CD006782. doi: 10.1002/14651858.CD006782.pub2.	No meta-analysis of interest.<br><br>Note: Meta-analyses were not performed by type of sport.
108. Gomes Neto M, Conceição CS, de Lima Brasileiro AJA, de Sousa CS, Carvalho VO, de Jesus FLA. Effects of the FIFA 11 training program on injury prevention and performance in football players: a systematic review and meta-analysis. Clin Rehabil. 2017 May;31(5):651-659. doi: 10.1177/0269215516675906.	No meta-analysis of interest.<br><br>Note: No overall meta-analyses or specific subgroups including all our inclusion criteria were found.
109. Goode AP, Reiman MP, Harris L, DeLisa L, Kauffman A, Beltramo D, Poole C, Ledbetter L, Taylor AB. Eccentric training for prevention of hamstring injuries may depend on intervention compliance: a systematic review and meta-analysis. Br J Sports Med. 2015 Mar;49(6):349-56. doi: 10.1136/bjsports-2014-093466.	No meta-analysis of interest.<br><br>Note: Meta-analyses were not performed by type of sport.

110. Grimm NL, Shea KG, Leaver RW, Aoki SK, Carey JL. Efficacy and degree of bias in knee injury prevention studies: a systematic review of RCTs. Clin Orthop Relat Res. 2013 Jan;471(1):308-16. doi: 10.1007/s11999-012-2565-3.	No meta-analysis.
111. Grimm NL, Jacobs JC Jr, Kim J, Denney BS, Shea KG. Anterior Cruciate Ligament and Knee Injury Prevention Programs for Soccer Players: A Systematic Review and Meta-analysis. Am J Sports Med. 2015 Aug;43(8):2049-56. doi: 10.1177/0363546514556737.	Included.
112. Grimm NL, Jacobs JC Jr, Kim J, Amendola A, Shea KG. Ankle Injury Prevention Programs for Soccer Athletes Are Protective: A Level-I Meta-Analysis. J Bone Joint Surg Am. 2016 Sep 7;98(17):1436-43. doi: 10.2106/JBJS.15.00933.	Included.
113. Gulati V, Jaggard M, Al-Nammari SS, Uzoigwe C, Gulati P, Ismail N, Gibbons C, Gupte C. Management of achilles tendon injury: A current concepts systematic review. World J Orthop. 2015 May 18;6(4):380-6. doi: 10.5312/wjo.v6.i4.380.	No meta-analysis.
114. Gustavsson J, Nilson F, Bonander C. Compliant sports floors and fall-related injuries: evidence from a residential care setting and updated meta-analysis for all patient care settings. Inj Prev. 2023 Aug;29(4):283-289. doi: 10.1136/ip-2022-044713.	No research design of interest.<br>Note: The authors updated previous meta-analyses, but the design of this study is not a systematic review.
115. Halappa NG. Integration of yoga within exercise and sports science as a preventive and management strategy for musculoskeletal injuries/disorders and mental disorders - A review of the literature. J Bodyw Mov Ther. 2023 Apr;34:34-40. doi: 10.1016/j.jbmt.2023.04.055.	No meta-analysis.
116. Halvorsen KC, Marx RG, Wolfe I, Taber C, Jivanelli B, Pearle AD, Ling DI. Higher Adherence to Anterior Cruciate Ligament Injury Prevention Programs Is Associated With Lower Injury Rates: A Meta-Analysis and Meta-Regression. HSS J. 2023 May;19(2):154-162. doi: 10.1177/15563316221140860.	No meta-analysis of interest.<br><br>Note: Meta-analyses were not performed by type of sport.
117. Hameed I, Farooq N, Haq A, Aimen I, Shanley J. Role of strengthening exercises in management and prevention of overuse sports injuries of lower extremity: a systematic review. J Sports Med Phys Fitness. 2024 Aug;64(8):807-815. doi: 10.23736/S0022-4707.23.15470-3.	No access at full text.<br><br>Note: This study was request to the original authors, but we did not receive the full text eventually.
118. Handoll HH, Almaiyah MA, Rangan A. Surgical versus non-surgical treatment for acute anterior shoulder dislocation. Cochrane Database Syst Rev. 2004;2004(1):CD004325. doi: 10.1002/14651858.CD004325.pub2.	The focus of the study was not primarily on sports populations.
119. Harrington TL, Breedlove GJ, Sharpe JJ. Systematic Review of Nonoperative Functional Protocols for Acute Achilles Ruptures Utilizing a Formal Rehabilitation Protocol Showing Lowest Rerupture Rates. Foot Ankle Spec. 2020 Dec;13(6):508-515. doi: 10.1177/1938640020916954.	No meta-analysis.

120. Harris JD, Griesser MJ, Best TM, Ellis TJ. Treatment of proximal hamstring ruptures - a systematic review. Int J Sports Med. 2011 Jul;32(7):490-5. doi: 10.1055/s-0031-1273753.	No meta-analysis.
121. Hart L. Effect of stretching on sport injury risk: a review. Clin J Sport Med. 2005 Mar;15(2):113. doi: 10.1097/01.jsm.0000151869.98555.67.	No objective of this study.
122. Herbert RD, Gabriel M. Effects of stretching before and after exercising on muscle soreness and risk of injury: systematic review. BMJ. 2002 Aug 31;325(7362):468. doi: 10.1136/bmj.325.7362.468.	No population of interest. Note: Two studies evaluated the risk of injuries, which were focused on military recruits.
123. Herman K, Barton C, Malliaras P, Morrissey D. The effectiveness of neuromuscular warm-up strategies, that require no additional equipment, for preventing lower limb injuries during sports participation: a systematic review. BMC Med. 2012 Jul 19;10:75. doi: 10.1186/1741-7015-10-75.	No meta-analysis.
124. Hewett TE, Myer GD, Ford KR. Reducing knee and anterior cruciate ligament injuries among female athletes: a systematic review of neuromuscular training interventions. J Knee Surg. 2005 Jan;18(1):82-8. doi: 10.1055/s-0030-1248163.	No meta-analysis.
125. Hewett TE, Ford KR, Myer GD. Anterior cruciate ligament injuries in female athletes: Part 2, a meta-analysis of neuromuscular interventions aimed at injury prevention. Am J Sports Med. 2006 Mar;34(3):490-8. doi: 10.1177/0363546505282619.	No meta-analysis of interest.<br><br>Note: Meta-analyses were not performed by type of sport.
126. Hewett TE, Myer GD, Ford KR, Paterno MV, Quatman CE. The 2012 ABJS Nicolas Andry Award: The sequence of prevention: a systematic approach to prevent anterior cruciate ligament injury. Clin Orthop Relat Res. 2012 Oct;470(10):2930-40. doi: 10.1007/s11999-012-2440-2.	No meta-analysis.
127. Hibbert O, Cheong K, Grant A, Beers A, Moizumi T. A systematic review of the effectiveness of eccentric strength training in the prevention of hamstring muscle strains in otherwise healthy individuals. N Am J Sports Phys Ther. 2008 May;3(2):67-81.	No meta-analysis.
128. Hickey JT, Timmins RG, Maniar N, Williams MD, Opar DA. Criteria for Progressing Rehabilitation and Determining Return-to-Play Clearance Following Hamstring Strain Injury: A Systematic Review. Sports Med. 2017 Jul;47(7):1375-1387. doi: 10.1007/s40279-016-0667-x.	No meta-analysis.
129. Holm PM, Juhl CB, Culvenor AG, Whittaker JL, Crossley KM, Roos EM, Patterson BE, Larsson S, Struglics A, Bricca A. The Effects of Different Management Strategies or Rehabilitation Approaches on Knee Joint Structural and Molecular Biomarkers Following Traumatic Knee Injury: A Systematic Review of Randomized Controlled Trials for the OPTIKNEE Consensus. J Orthop Sports Phys Ther. 2023 Apr;53(4):172-193. doi: 10.2519/jospt.2023.11576.	No meta-analysis.
130. Hollabaugh WL, Sin A, Walden RL, Weaver JS, Porras LP, LeClere LE, Karpinos AR, Coronado RA, Gregory AJ, Sullivan JP. Outcomes of Activity-Related Lower Extremity Muscle Tears After Application of the British Athletics Muscle Injury Classification: A Systematic Review. Sports Health. 2024 Sep-Oct;16(5):783-796. doi: 10.1177/19417381231195529.	No meta-analysis.
131. Hoppe MW, Brochhagen J, Tischer T, Beitzel K, Seil R, Grim C. Risk factors and prevention strategies for shoulder injuries in overhead sports: an updated systematic review. J Exp Orthop. 2022 Aug 16;9(1):78. doi: 10.1186/s40634-022-00493-9.	No meta-analysis.

132. Hu C, Du Z, Tao M, Song Y. Effects of Different Hamstring Eccentric Exercise Programs on Preventing Lower Extremity Injuries: A Systematic Review and Meta-Analysis. Int J Environ Res Public Health. 2023 Jan 23;20(3):2057. doi: 10.3390/ijerph20032057.	No meta-analysis of interest.<br><br>Note: Meta-analyses were not performed by type of sport.
133. Huang YL, Jung J, Mulligan CMS, Oh J, Norcross MF. A Majority of Anterior Cruciate Ligament Injuries Can Be Prevented by Injury Prevention Programs: A Systematic Review of Randomized Controlled Trials and Cluster-Randomized Controlled Trials With Meta-analysis. Am J Sports Med. 2020 May;48(6):1505-1515. doi: 10.1177/0363546519870175.	No meta-analysis of interest.<br><br>Note: Meta-analyses were not performed by type of sport.
134. Hübscher M, Zech A, Pfeifer K, Hänsel F, Vogt L, Banzer W. Neuromuscular training for sports injury prevention: a systematic review. Med Sci Sports Exerc. 2010 Mar;42(3):413-21. doi: 10.1249/MSS.0b013e3181b88d37.	No meta-analysis of interest.<br><br>Note: Meta-analyses were not performed by type of sport.
135. Hume PA, Lorimer AV, Griffiths PC, Carlson I, Lamont M. Recreational Snow-Sports Injury Risk Factors and Countermeasures: A Meta-Analysis Review and Haddon Matrix Evaluation. Sports Med. 2015 Aug;45(8):1175-90. doi: 10.1007/s40279-015-0334-7.	No intervention of interest.
136. Hutton MJ, McGuire RA, Dunn R, Williams R, Robertson P, Twaddle B, Kiely P, Clarke A, Mazda K, Davies P, Pagarigan KT, Dettori JR. Catastrophic Cervical Spine Injuries in Contact Sports. Global Spine J. 2016 Nov;6(7):721-734. doi: 10.1055/s-0036-1586744.	No intervention of interest.
137. Impellizzeri FM, McCall A, van Smeden M. Why methods matter in a meta-analysis: a reappraisal showed inconclusive injury preventive effect of Nordic hamstring exercise. J Clin Epidemiol. 2021 Dec;140:111-124. doi: 10.1016/j.jclinepi.2021.09.007.	No meta-analysis of interest.<br><br>Note: Meta-analyses were not performed by type of sport.
138. Ishøi L, Krommes K, Husted RS, Juhl CB, Virgile A, Thorborg K. Infographic. Diagnosis, prevention and treatment of common lower extremity muscle injuries in sport-grading the evidence: a statement paper commissioned by the Danish Society of Sports Physical Therapy (DSSF). Br J Sports Med. 2020 Sep;54(18):1116-1117. doi: 10.1136/bjsports-2020-102119.	No research design of interest.
139. Jamjoom BA. The Influence of Early Weightearing, Controlled Motion, and Timing of Orthosis Removal on the Nonoperative Management of Achilles Tendon Rupture: A Systematic Review. J Foot Ankle Surg. 2021 Jul-Aug;60(4):777-786. doi: 10.1053/j.jfas.2020.04.024.	The focus of the study was not primarily on sports populations.
140. Jankaew A, Chen JC, Chamnongkich S, Lin CF. Therapeutic Exercises and Modalities in Athletes With Acute Hamstring Injuries: A Systematic Review and Meta-analysis. Sports Health. 2023 Jul-Aug;15(4):497-511. doi: 10.1177/19417381221118085.	No meta-analysis of interest.<br><br>Note: Meta-analyses were

	not performed by type of sport.
141. Jiang N, Wang B, Chen A, Dong F, Yu B. Operative versus nonoperative treatment for acute Achilles tendon rupture: a meta-analysis based on current evidence. Int Orthop. 2012 Apr;36(4):765-73. doi: 10.1007/s00264-011-1431-3.	The focus of the study was not primarily on sports populations.
142. Jimenez-Olmedo JM, Penichet-Tomas A. Injuries and pathologies in beach volleyball players: A systematic review. Journal of Human Sport & Exercise. 2015;10(4):936-948.	No meta-analysis.
143. Jokela A, Stenroos A, Kosola J, Valle X, Lempainen L. A systematic review of surgical intervention in the treatment of hamstring tendon ruptures: current evidence on the impact on patient outcomes. Ann Med. 2022 Dec;54(1):978-988. doi: 10.1080/07853890.2022.2059560.	No intervention of interest.
144. Jones BH, Thacker SB, Gilchrist J, Kimsey CD Jr, Sosin DM. Prevention of lower extremity stress fractures in athletes and soldiers: a systematic review. Epidemiol Rev. 2002;24(2):228-47. doi: 10.1093/epirev/mxf011.	No meta-analysis.
145. Jones MH, Amendola AS. Acute treatment of inversion ankle sprains: immobilization versus functional treatment. Clin Orthop Relat Res. 2007 Feb;455:169-72. doi: 10.1097/BLO.0b013e31802f5468.	The focus of the study was not primarily on sports population.
146. Jones G, Schöffl V, Johnson MI. Incidence, Diagnosis, and Management of Injury in Sport Climbing and Bouldering: A Critical Review. Curr Sports Med Rep. 2018 Nov;17(11):396-401. doi: 10.1249/JSR.0000000000000534.	No meta-analysis.
147. Jordan RW, Saithna A, Old J, MacDonald P. Does external rotation bracing for anterior shoulder dislocation actually result in reduction of the labrum? A systematic review. Am J Sports Med. 2015 Sep;43(9):2328-33. doi: 10.1177/0363546514555661.	No meta-analysis.
148. Kerkhoffs GM, Handoll HH, de Bie R, Rowe BH, Struijs PA. Surgical versus conservative treatment for acute injuries of the lateral ligament complex of the ankle in adults. Cochrane Database Syst Rev. 2007 Apr 18;(2):CD000380. doi: 10.1002/14651858.CD000380.pub2.	The focus of the study was not primarily on sports populations.
149. Kilic O, Maas M, Verhagen E, Zwerver J, Gouttebarge V. Incidence, aetiology and prevention of musculoskeletal injuries in volleyball: A systematic review of the literature. Eur J Sport Sci. 2017 Jul;17(6):765-793. doi: 10.1080/17461391.2017.1306114.	No meta-analysis.
150. Kilic Ö, Van Os V, Kemler E, Barendrecht M, Gouttebarge V. The 'Sequence of Prevention' for musculoskeletal injuries among recreational basketballers: a systematic review of the scientific literature. Phys Sportsmed. 2018 May;46(2):197-212. doi: 10.1080/00913847.2018.1424496.	No meta-analysis.
151. Kilic O, Kemler E, Gouttebarge V. The "sequence of prevention" for musculoskeletal injuries among adult recreational footballers: A systematic review of the scientific literature. Phys Ther Sport. 2018 Jul;32:308-322. doi: 10.1016/j.ptsp.2018.01.007.	No meta-analysis.
152. Kirkendall DT, Junge A, Dvorak J. Prevention of football injuries. Asian J Sports Med. 2010 Jun;1(2):81-92. doi: 10.5812/asjsm.34869.	No meta-analysis.
153. Knapik DM, Gilmore A, Liu RW. Conservative Management of Minimally Displaced (≤2 mm) Fractures of the Lateral Humeral Condyle in Pediatric Patients: A Systematic Review. J Pediatr Orthop. 2017 Mar;37(2):e83-e87. doi: 10.1097/BPO.0000000000000722.	No meta-analysis.
154. Knapik DM, Voos JE. Anterior Cruciate Ligament Injuries in Skeletally Immature Patients: A Meta-analysis Comparing Repair Versus Reconstruction Techniques. J Pediatr Orthop. 2020 Oct;40(9):492-502. doi: 10.1097/BPO.0000000000001569.	No intervention of interest.

155. Kodikara D, Twomey DM, Plumb MS. A Systematic Review of Head, Neck and-Facial Injuries in Cricket. Int J Sports Med. 2022 Jun;43(6):496-504. doi: 10.1055/a-1684-9033.	No intervention of interest.
156. Koerber S, Wager SG, Zynda AJ, Santa Barbara MT. A Scoping Review: Reducing Musculoskeletal Injury Risk Factors for Adaptive Sport Athletes through Prevention Programs. Am J Phys Med Rehabil. 2024 Mar 25. doi: 10.1097/PHM.0000000000002490.	No research design of interest.
157. Kraeutler MJ, Belk JW, Carver TJ, McCarty EC, Khodaee M. Traumatic Primary Anterior Glenohumeral Joint Dislocation in Sports: A Systematic Review of Operative versus Nonoperative Management. Curr Sports Med Rep. 2020 Nov;19(11):468-478. doi: 10.1249/JSR.0000000000000772.	No meta-analysis.
158. Kresal F, Bracun S, Tonig L, Amon M. Physical therapy recommendations for injury prevention in alpine skiing. Annales Kinesiologiae. 2021;12(1):43-55.	No meta-analysis.
159. Krumbach B, Meretsky C, Schiuma AT, Ajebli M. A Comparative Analysis of Quadriceps Tendon, Patellar Tendon Bone Allograft, and Cadaver Graft in Anterior Cruciate Ligament (ACL) Repair and Reconstructive Surgery. Cureus. 2024 May 7;16(5):e59836. doi: 10.7759/cureus.59836.	No meta-analysis.
160. Lachance CC, Jurkowski MP, Dymarz AC, Mackey DC. Compliant flooring to prevent fall-related injuries: a scoping review protocol. BMJ Open. 2016 Aug 16;6(8):e011757. doi: 10.1136/bmjopen-2016-011757.	No research design of interest.
161. Lavigne A, Chicoine D, Esculier JF, Desmeules F, Frémont P, Dubois B. The Role of Footwear, Foot Orthosis, and Training-Related Strategies in the Prevention of Bone Stress Injuries: A Systematic Review and Meta-Analysis. Int J Exerc Sci. 2023 Jun 1;16(3):721-743.	No population of interest.
162. Lemes IR, Pinto RZ, Lage VN, Roch BAB, Verhagen E, Bolling C, Aquino CF, Fonseca ST, Souza TR. Do exercise-based prevention programmes reduce non-contact musculoskeletal injuries in football (soccer)? A systematic review and meta-analysis with 13 355 athletes and more than 1 million exposure hours. Br J Sports Med. 2021 Oct;55(20):1170-1178. doi: 10.1136/bjsports-2020-103683.	Included.
163. Leppänen M, Aaltonen S, Parkkari J, Heinonen A, Kujala UM. Interventions to prevent sports related injuries: a systematic review and meta-analysis of randomised controlled trials. Sports Med. 2014 Apr;44(4):473-86. doi: 10.1007/s40279-013-0136-8.	No meta-analysis of interest.<br><br>Note: No subgroups including specific sports and musculoskeletal body regions (e.g., ankle injuries in basketball) were conducted.
164. Lategan L, Gouveia CP. Prevention of hamstring injuries in sport: A systematic review. South African Journal for Research in Sport, Physical Education and Recreation. 2018;40(1):55-69.	No meta-analysis.
165. Lauersen JB, Andersen TE, Andersen LB. Strength training as superior, dose-dependent and safe prevention of acute and overuse sports injuries: a systematic review, qualitative analysis and meta-analysis. Br J Sports Med. 2018 Dec;52(24):1557-1563. doi: 10.1136/bjsports-2018-099078.	No meta-analysis of interest.<br><br>Note: Meta-analyses were not performed by type of sport.

166. Lewis J. A systematic literature review of the relationship between stretching and athletic injury prevention. Orthop Nurs. 2014 Nov-Dec;33(6):312-20; quiz 321-2. doi: 10.1097/NOR.0000000000000097.	No meta-analysis.
167. Liddle N, Taylor JM, Chesterton P, Atkinson G. The Effects of Exercise-Based Injury Prevention Programmes on Injury Risk in Adult Recreational Athletes: A Systematic Review and Meta-Analysis. Sports Med. 2024 Mar;54(3):645-658. doi: 10.1007/s40279-023-01950-w.	No meta-analysis of interest.<br><br>Note: Meta-analyses were not performed by type of sport.
168. Liu X, Dai TJ, Li BL, Li C, Zheng ZY, Liu Y. Early functional rehabilitation compared with traditional immobilization for acute Achilles tendon ruptures : a meta-analysis. Bone Joint J. 2021 Jun;103-B(6):1021-1030. doi: 10.1302/0301-620X.103B6.BJJ-2020-1890.R1.	No access at full text.<br><br>Note: This study was request to the original authors, but an answer was not received.
169. Longo UG, Loppini M, Rizzello G, Ciuffreda M, Maffulli N, Denaro V. Management of primary acute anterior shoulder dislocation: systematic review and quantitative synthesis of the literature. Arthroscopy. 2014 Apr;30(4):506-22. doi: 10.1016/j.arthro.2014.01.003.	The focus of the study was not primarily on sports populations.
170. Longo UG, van der Linde JA, Loppini M, Coco V, Poolman RW, Denaro V. Surgical Versus Nonoperative Treatment in Patients Up to 18 Years Old With Traumatic Shoulder Instability: A Systematic Review and Quantitative Synthesis of the Literature. Arthroscopy. 2016 May;32(5):944-52. doi: 10.1016/j.arthro.2015.10.020.	The focus of the study was not primarily on sports populations.
171. Lopes JSS, Machado AF, Cavina AP, Michelletti JK, de Almeida AC, Pastre CM. Specific interventions for prevention of muscle injury in lower limbs: systematic review and meta-analysis. Fisioter Mov. 2019;32:1-12.	No meta-analysis of interest.<br><br>Note: Meta-analyses were not performed by type of sport.
172. Luijten SCM, Nauta J, Janssen T, Holla J, Jenniskens SCN, Verhagen E. Systematic development of an injury and illness prevention programme for athletes with a physical impairment: the TIPAS study. BMJ Open Sport Exerc Med. 2024 Aug 7;10(3):e001945. doi: 10.1136/bmjsem-2024-001945.	No meta-analysis.
173. Lutz D, van den Berg C, Räisänen AM, Shill IJ, Kim J, Vaandering K, Hayden A, Pasanen K, Schneider KJ, Emery CA, Owoeye OBA. Best practices for the dissemination and implementation of neuromuscular training injury prevention warm-ups in youth team sport: a systematic review. Br J Sports Med. 2024 May 28;58(11):615-625. doi: 10.1136/bjsports-2023-106906.	No meta-analysis.
174. Magaña-Ramírez M, Gallardo-Gómez D, Álvarez-Barbosa F, Corral-Pernía JA. What exercise programme is the most appropriate to mitigate anterior cruciate ligament injury risk in football (soccer) players? A systematic review and network meta-analysis. J Sci Med Sport. 2024 Apr;27(4):234-242. doi: 10.1016/j.jsams.2024.02.001.	No direct comparison in meta-analyses were reported.
175. Massen FK, Shoap SC, Vosseller T, Fan W, Usseglio J, Boecker WD, Baumbach SF, Polzer H. Rehabilitation following operative treatment of acute Achilles tendon ruptures: a systematic review and meta-analysis. EFORT Open Reviews. 2022;7:680-691.	The focus of the study was not primarily on sports populations.

176. Mark-Christensen T, Troelsen A, Kallemose T, Barfod KW. Functional rehabilitation of patients with acute Achilles tendon rupture: a meta-analysis of current evidence. Knee Surg Sports Traumatol Arthrosc. 2016 Jun;24(6):1852-9. doi: 10.1007/s00167-014-3180-5.	The focus of the study was not primarily on sports populations.
177. Marois B, Tan XW, Pauyo T, Dodin P, Ballaz L, Nault ML. Can a Knee Brace Prevent ACL Reinjury: A Systematic Review. Int J Environ Res Public Health. 2021 Jul 17;18(14):7611. doi: 10.3390/ijerph18147611.	No intervention of interest.
178. Mason DL, Dickens VA, Vail A. Rehabilitation for hamstring injuries. Cochrane Database Syst Rev. 2012 Dec 12;12:CD004575. doi: 10.1002/14651858.CD004575.pub3.	No meta-analysis of interest.
179. Mattu AT, Ghali B, Linton V, Zheng A, Pike I. Prevention of Non-Contact Anterior Cruciate Ligament Injuries among Youth Female Athletes: An Umbrella Review. Int J Environ Res Public Health. 2022 Apr 12;19(8):4648. doi: 10.3390/ijerph19084648.	No research design of interest.
180. Mazarelo JFD, Winter SL, Fong DTP. A Systematic Review on the Effectiveness of Eyewear in Reducing the Incidence and Severity of Eye Injuries in Racket Sports. Phys Sportsmed. 2024 Apr;52(2):115-124. doi: 10.1080/00913847.2023.2196934.	No intervention of interest.
181. McCall A, Carling C, Davison M, Nedelec M, Le Gall F, Berthoin S, Dupont G. Injury risk factors, screening tests and preventative strategies: a systematic review of the evidence that underpins the perceptions and practices of 44 football (soccer) teams from various premier leagues. Br J Sports Med. 2015 May;49(9):583-9. doi: 10.1136/bjsports-2014-094104.	No meta-analysis.
182. McCormack RG. Surgery or conservative treatment for acute achilles tendon ruptures: a meta-analysis. Clin J Sport Med. 2003 May;13(3):194. doi: 10.1097/00042752-200305000-00016.	No research design of interest.
183. McCrary JM, Ackermann BJ, Halaki M. A systematic review of the effects of upper body warm-up on performance and injury. Br J Sports Med. 2015 Jul;49(14):935-42. doi: 10.1136/bjsports-2014-094228.	No meta-analysis of interest.
184. McMahon SE, Smith TO, Hing CB. A meta-analysis of randomised controlled trials comparing conventional to minimally invasive approaches for repair of an Achilles tendon rupture. Foot Ankle Surg. 2011 Dec;17(4):211-7. doi: 10.1016/j.fas.2010.11.001.	No intervention of interest.
185. Meulenkamp B, Stacey D, Fergusson D, Hutton B, Mlis RS, Graham ID. Protocol for treatment of Achilles tendon ruptures; a systematic review with network meta-analysis. Syst Rev. 2018 Dec 23;7(1):247. doi: 10.1186/s13643-018-0912-5.	No research design of interest.
186. Michaelidis M, Koumantakis GA. Effects of knee injury primary prevention programs on anterior cruciate ligament injury rates in female athletes in different sports: a systematic review. Phys Ther Sport. 2014 Aug;15(3):200-10. doi: 10.1016/j.ptsp.2013.12.002.	No meta-analysis.
187. Moiz M, Smith N, Smith TO, Chawla A, Thompson P, Metcalfe A. Clinical Outcomes After the Nonoperative Management of Lateral Patellar Dislocations: A Systematic Review. Orthop J Sports Med. 2018 Jun 11;6(6):2325967118766275. doi: 10.1177/2325967118766275.	No meta-analysis.
188. Mosca M, Fuiano M, Massimi S, Censoni D, Catanese G, Grassi A, Caravelli S, Zaffagnini S. Ruptures of the Plantar Fascia: A Systematic Review of the Literature. Foot Ankle Spec. 2022 Jun;15(3):272-282. doi: 10.1177/1938640020974889.	No meta-analysis.
189. Mugele H, Plummer A, Steffen K, Stoll J, Mayer F, Müller J. General versus sports-specific injury prevention programs in athletes: A systematic review on the effect on injury rates. PLoS One. 2018 Oct 19;13(10):e0205635. doi: 10.1371/journal.pone.0205635.	No meta-analysis.
190. Müller I. Profylaxe poskození pohybového aparátu intenzívním sportem [Prevention of locomotor apparatus damage through intensive sports (author's transl)]. Acta Chir Orthop Traumatol Cech. 1979 Apr;46(2):176-8. Czech.	No access at full text.
191. Mulvaney CA, Smith S, Watson MC, Parkin J, Coupland C, Miller P, Kendrick D, McClintock H. Cycling infrastructure for reducing cycling injuries in cyclists.	No intervention of interest.

Cochrane Database Syst Rev. 2015 Dec 10;2015(12):CD010415. doi: 10.1002/14651858.CD010415.pub2.	
192. Munro J, Coleman P, Nicholl J, Harper R, Kent G, Wild D. Can we prevent accidental injury to adolescents? A systematic review of the evidence. Inj Prev. 1995 Dec;1(4):249-55. doi: 10.1136/ip.1.4.249.	No meta-analysis.
193. Myer GD, Sugimoto D, Thomas S, Hewett TE. The influence of age on the effectiveness of neuromuscular training to reduce anterior cruciate ligament injury in female athletes: a meta-analysis. Am J Sports Med. 2013 Jan;41(1):203-15. doi: 10.1177/0363546512460637.	No meta-analysis of interest.<br><br>Note: Meta-analyses were not performed by type of sport.
194. Naderi A, Shaabani F, Keikha M, Degens H. Is an Exercise-Based Injury-Prevention Program Effective in Team Handball Players? A Systematic Review and Meta-Analysis. J Athl Train. 2024 Aug 1;59(8):845-856. doi: 10.4085/1062-6050-0680.22.	No meta-analysis of interest.<br><br>Note: This review included randomized and non-randomized trials. However, no subgroups by research design were conducted.
195. Nauta J, van Mechelen W, Otten RH, Verhagen EA. A systematic review on the effectiveness of school and community-based injury prevention programmes on risk behaviour and injury risk in 8-12 year old children. J Sci Med Sport. 2014 Mar;17(2):165-72. doi: 10.1016/j.jsams.2013.07.011.	No meta-analysis.
196. Noyes FR, Barber Westin SD. Anterior cruciate ligament injury prevention training in female athletes: a systematic review of injury reduction and results of athletic performance tests. Sports Health. 2012 Jan;4(1):36-46. doi: 10.1177/1941738111430203.	No meta-analysis.
197. Noyes FR, Barber-Westin SD. Neuromuscular retraining intervention programs: do they reduce noncontact anterior cruciate ligament injury rates in adolescent female athletes? Arthroscopy. 2014 Feb;30(2):245-55. doi: 10.1016/j.arthro.2013.10.009.	No meta-analysis.
198. Nugent EP. muscle injuries in female athletes: a scoping review. Journal of Australian Strength & Conditioning. 2023;31(5):53-59.	No research design of interest.<br><br>Note: We have not access to the full text. However, the abstract section did not specify that our objective could be reached. For example, the authors describe possible interventions to reduce the risk of quadriceps

	strains. However, they also state that no preventive studies were found for quadriceps strains.
199. Nunes H, Fernandes LG, Martins PN, Ferreira RM. The Effects of Nordic Hamstring Exercise on Performance and Injury in the Lower Extremities: An Umbrella Review. Healthcare (Basel). 2024 Jul 23;12(15):1462. doi: 10.3390/healthcare12151462.	No research design of interest.
200. Nwachukwu BU, So C, Schairer WW, Green DW, Dodwell ER. Surgical versus conservative management of acute patellar dislocation in children and adolescents: a systematic review. Knee Surg Sports Traumatol Arthrosc. 2016 Mar;24(3):760-7. doi: 10.1007/s00167-015-3948-2.	The focus of the study was not primarily on sports populations.
201. Obërtinca R, Hoxha I, Meha R, Lama A, Bimbashi A, Kuqi D, Shabani B, Meyer T, der Fünten KA. Efficacy of Multi-Component Exercise-Based Injury Prevention Programs on Injury Risk Among Footballers of All Age Groups: A Systematic Review and Meta-analysis. Sports Med. 2023 Apr;53(4):837-848. doi: 10.1007/s40279-022-01797-7.	Included.
202. Okobi OE, Evbayekha EO, Ilechie E, Iroro J, Nwafor JN, Gandu Z, Shittu HO. A Meta-Analysis of Randomized Controlled Trials on the Effectiveness of Exercise Intervention in Preventing Sports Injuries. Cureus. 2022 Jun 20;14(6):e26123. doi: 10.7759/cureus.26123.	No meta-analysis of interest.<br><br>Note: No overall meta-analyses or specific subgroups including all our inclusion criteria were found.
203. Olivares-Jabalera J, Fílter-Ruger A, Dos'Santos T, Afonso J, Della Villa F, Morente-Sánchez J, Soto-Hermoso VM, Requena B. Exercise-Based Training Strategies to Reduce the Incidence or Mitigate the Risk Factors of Anterior Cruciate Ligament Injury in Adult Football (Soccer) Players: A Systematic Review. Int J Environ Res Public Health. 2021 Dec 18;18(24):13351. doi: 10.3390/ijerph182413351.	No meta-analysis.
204. Oliveira JP, Sampaio T, Marinho DA, Barbosa TM, Morais JE. Exploring Injury Prevention Strategies for Futsal Players: A Systematic Review. Healthcare (Basel). 2024 Jul 11;12(14):1387. doi: 10.3390/healthcare12141387.	No meta-analysis.
205. Owoeye OBA, VanderWey MJ, Pike I. Reducing Injuries in Soccer (Football): an Umbrella Review of Best Evidence Across the Epidemiological Framework for Prevention. Sports Med Open. 2020 Sep 21;6(1):46. doi: 10.1186/s40798-020-00274-7.	No research design of interest.
206. Papaleontiou A, Poupard AM, Mahajan UD, Tsantanis P. Conservative vs Surgical Treatment of Anterior Cruciate Ligament Rupture: A Systematic Review. Cureus. 2024 Mar 20;16(3):e56532. doi: 10.7759/cureus.56532.	No meta-analysis.
207. Papalia R, Tecame A, Torre G, Narbona P, Maffulli N, Denaro V. Rugby and Shoulder Trauma: A Systematic Review. Transl Med UniSa. 2014 Sep 1;12:5-13.	No meta-analysis.
208. Parkkari J, Kujala UM, Kannus P. Is it possible to prevent sports injuries? Review of controlled clinical trials and recommendations for future work. Sports Med. 2001;31(14):985-95. doi: 10.2165/00007256-200131140-00003.	No meta-analysis.
209. Pas HI, Reurink G, Tol JL, Weir A, Winters M, Moen MH. Efficacy of rehabilitation (lengthening) exercises, platelet-rich plasma injections, and other conservative interventions in acute hamstring injuries: an updated systematic review and meta-analysis. Br J Sports Med. 2015 Sep;49(18):1197-205. doi: 10.1136/bjsports-2015-094879.	No meta-analysis of interest.

	Note: The meta-analysis associated with hamstring exercises included different types of sports (e.g., soccer and track and field athletes)
**210.** Paul J, Brown SM, Mulcahey MK. Injury Prevention Programs for Throwing Injuries in Softball Players: A Systematic Review. Sports Health. 2021 Jul-Aug;13(4):390-395. doi: 10.1177/1941738120978161.	No meta-analysis.
211. Paulino Pereira NR, van der Linde JA, Alkaduhimi H, Longo UG, van den Bekerom MPJ. Are collision athletes at a higher risk of re-dislocation after an open Bristow-Latarjet procedure? A systematic review and meta-analysis. Shoulder Elbow. 2018 Apr;10(2):75-86. doi: 10.1177/1758573217728290.	No intervention of interest.
212. Peden AE, Cullen P, Bhandari B, Testa L, Wang A, Ma T, Möller H, Peden M, Sawyer SM, Ivers R. A systematic review of the evidence for effectiveness of interventions to address transport and other unintentional injuries among adolescents. J Safety Res. 2023 Jun;85:321-338. doi: 10.1016/j.jsr.2023.03.005.	No meta-analysis.
213. Pérez-Gómez J, Adsuar JC, Alcaraz PE, Carlos-Vivas J. Physical exercises for preventing injuries among adult male football players: A systematic review. J Sport Health Sci. 2022 Jan;11(1):115-122. doi: 10.1016/j.jshs.2020.11.003.	No meta-analysis.
214. Perrott MA, Pizzari T, Cook J. Lumbopelvic exercise reduces lower limb muscle strain injury in recreational athletes. Physical Therapy Reviews. 2013;18(1):24-33.	No meta-analysis of interest.<br>Note: Military recruits were considered, and no specific subgroups for athletes were performed.
215. Peters JA, Zwerver J, Diercks RL, Elferink-Gemser MT, van den Akker-Scheek I. Preventive interventions for tendinopathy: A systematic review. J Sci Med Sport. 2016 Mar;19(3):205-211. doi: 10.1016/j.jsams.2015.03.008.	No meta-analysis.
216. Petersen W, Rembitzki IV, Koppenburg AG, Ellermann A, Liebau C, Brüggemann GP, Best R. Treatment of acute ankle ligament injuries: a systematic review. Arch Orthop Trauma Surg. 2013 Aug;133(8):1129-41. doi: 10.1007/s00402-013-1742-5.	No meta-analysis.
217. Petushek EJ, Sugimoto D, Stoolmiller M, Smith G, Myer GD. Evidence-Based Best-Practice Guidelines for Preventing Anterior Cruciate Ligament Injuries in Young Female Athletes: A Systematic Review and Meta-analysis. Am J Sports Med. 2019 Jun;47(7):1744-1753. doi: 10.1177/0363546518782460.	No meta-analysis of interest.<br><br>Note: No overall meta-analyses or specific subgroups including all our inclusion criteria were found.

218. Pfile KR, Curioz B. Coach-led prevention programs are effective in reducing anterior cruciate ligament injury risk in female athletes: A number-needed-to-treat analysis. Scand J Med Sci Sports. 2017 Dec;27(12):1950-1958. doi: 10.1111/sms.12828.	No meta-analysis of interest.<br><br>Note: Meta-analyses were not performed by type of sport.
219. Pietrosimone BG, Grindstaff TL, Linens SW, Uczekaj E, Hertel J. A systematic review of prophylactic braces in the prevention of knee ligament injuries in collegiate football players. J Athl Train. 2008 Jul-Aug;43(4):409-15. doi: 10.4085/1062-6050-43.4.409.	No intervention of interest.
220. Porter T, Rushton A. The efficacy of exercise in preventing injury in adult male football: a systematic review of randomised controlled trials. Sports Med Open. 2015 Dec;1(1):4. doi: 10.1186/s40798-014-0004-6.	No meta-analysis.
221. Poursalehian M, Lotfi M, Zafarmandi S, Arabzadeh Bahri R, Halabchi F. Hamstring Injury Treatments and Management in Athletes: A Systematic Review of the Current Literature. JBJS Rev. 2023 Nov 20;11(11). doi: 10.2106/JBJS.RVW.23.00161.	No meta-analysis.
222. Preiss A, Brodhun T, Stietencron I, Frosch KH. Die vordere Kreuzbandruptur im Wachstumsalter - operativ oder konservativ? Ein systematisches Review [Rupture of the anterior cruciate ligament in growing children: surgical or conservative treatment? A systematic review]. Unfallchirurg. 2012 Sep;115(9):848-54. German. doi: 10.1007/s00113-012-2215-y.	No meta-analysis.
223. Prior M, Guerin M, Grimmer K. An evidence-based approach to hamstring strain injury: a systematic review of the literature. Sports Health. 2009 Mar;1(2):154-64. doi: 10.1177/1941738108324962.	No meta-analysis.
224. Prodromos CC, Han Y, Rogowski J, Joyce B, Shi K. A meta-analysis of the incidence of anterior cruciate ligament tears as a function of gender, sport, and a knee injury-reduction regimen. Arthroscopy. 2007 Dec;23(12):1320-1325.e6. doi: 10.1016/j.arthro.2007.07.003.	No meta-analysis of interest.<br><br>Note: Although this study reported supplementary material (we have no access), the authors did not specify in the whole manuscript the type of design of the intervention programs.
225. Quarmby A, Zhang M, Geisler M, Javorsky T, Mugele H, Cassel M, Lawley J. Risk factors and injury prevention strategies for overuse injuries in adult climbers: a systematic review. Front Sports Act Living. 2023 Dec 12;5:1269870. doi: 10.3389/fspor.2023.1269870.	No meta-analysis.
226. Ramirez RN, Baldwin K, Franklin CCD. Prevention of Anterior Cruciate Ligament Rupture in Female Athletes: A Systematic Review. JBJS Rev. 2014 Sep 16;2(9):e3. doi: 10.2106/JBJS.RVW.M.00129.	No meta-analysis of interest.<br><br>Note: No overall meta-analyses or specific subgroups including all our inclusion

	criteria were found.
227. Ramos AP, de Mesquita RS, Migliorini F, Maffulli N, Okubo R. FIFA 11+ KIDS in the prevention of soccer injuries in children: a systematic review. J Orthop Surg Res. 2024 Jul 18;19(1):413. doi: 10.1186/s13018-024-04876-9.	No meta-analysis.
228. Rauch S, Wallner B, Ströhle M, Dal Cappello T, Brodmann Maeder M. Climbing Accidents-Prospective Data Analysis from the International Alpine Trauma Registry and Systematic Review of the Literature. Int J Environ Res Public Health. 2019 Dec 27;17(1):203. doi: 10.3390/ijerph17010203.	No meta-analysis.
229. Raya-Gonzalez J, Castillo D, Clemente FM. Injury prevention of hamstring injuries through exercise interventions. J Sports Med Phys Fitness. 2021 Sep;61(9):1242-1251. doi: 10.23736/S0022-4707.21.11670-6.	No research design of interest.
230. Reda Y, Farouk A, Abdelmonem I, El Shazly OA. Surgical versus non-surgical treatment for acute Achilles' tendon rupture. A systematic review of literature and meta-analysis. Foot Ankle Surg. 2020 Apr;26(3):280-288. doi: 10.1016/j.fas.2019.03.010.	The focus of the study was not primarily on sports populations.
231. Ripley NJ, Cuthbert M, Ross S, Comfort P, McMahon JJ. The Effect of Exercise Compliance on Risk Reduction for Hamstring Strain Injury: A Systematic Review and Meta-Analyses. Int J Environ Res Public Health. 2021 Oct 27;18(21):11260. doi: 10.3390/ijerph182111260.	Included.
232. Robles-Palazón FJ, Cejudo A, Ayala F, Sainz de Baranda P. Características de las estrategias de prevención de lesiones en niños y adolescentes deportistas. Revisión sistemática. Journal of Sport and Health Research. 2019;11(1):1-16.	No meta-analysis.
233. Robles-Palazón FJ, Blázquez-Rincón D, López-Valenciano A, Comfort P, López-López JA, Ayala F. A systematic review and network meta-analysis on the effectiveness of exercise-based interventions for reducing the injury incidence in youth team-sport players. Part 1: an analysis by classical training components. Ann Med. 2024 Dec;56(1):2408457. doi: 10.1080/07853890.2024.2408457.	No meta-analysis of interest.<br><br>Note: Meta-analyses were not performed by type of sport.
234. Rogan S, Wüst D, Schwitter T, Schmidtbleicher D. Static stretching of the hamstring muscle for injury prevention in football codes: a systematic review. Asian J Sports Med. 2013 Mar;4(1):1-9.	No meta-analysis.
235. Rome K, Handoll HH, Ashford R. Interventions for preventing and treating stress fractures and stress reactions of bone of the lower limbs in young adults. Cochrane Database Syst Rev. 2005 Apr 18;2005(2):CD000450. doi: 10.1002/14651858.CD000450.pub2.	The focus of the study was not primarily on sports populations.
236. Rosado-Portillo A, Chamorro-Moriana G, Gonzalez-Medina G, Perez-Cabezas V. Acute Hamstring Injury Prevention Programs in Eleven-a-Side Football Players Based on Physical Exercises: Systematic Review. J Clin Med. 2021 May 9;10(9):2029. doi: 10.3390/jcm10092029.	No meta-analysis.
237. Rössler R, Donath L, Verhagen E, Junge A, Schweizer T, Faude O. Exercise-based injury prevention in child and adolescent sport: a systematic review and meta-analysis. Sports Med. 2014 Dec;44(12):1733-48. doi: 10.1007/s40279-014-0234-2.	No meta-analysis of interest.<br><br>Note: No overall meta-analyses or specific subgroups including all our inclusion criteria were found.

238. Rowe PL, Bryant AL, Paterson KL. Current ankle sprain prevention and management strategies of netball athletes: a scoping review of the literature and comparison with best-practice recommendations. BMC Sports Sci Med Rehabil. 2021 Sep 18;13(1):113. doi: 10.1186/s13102-021-00342-9.	No research design of interest.
239. Rudisill SS, Kucharik MP, Varady NH, Martin SD. Evidence-Based Management and Factors Associated With Return to Play After Acute Hamstring Injury in Athletes: A Systematic Review. Orthop J Sports Med. 2021 Nov 29;9(11):23259671211053833. doi: 10.1177/23259671211053833.	No meta-analysis.
240. Rudisill SS, Varady NH, Kucharik MP, Eberlin CT, Martin SD. Evidence-Based Hamstring Injury Prevention and Risk Factor Management: A Systematic Review and Meta-analysis of Randomized Controlled Trials. Am J Sports Med. 2023 Jun;51(7):1927-1942. doi: 10.1177/03635465221083998.	No meta-analysis of interest.<br><br>Note: Meta-analyses were not performed by type of sport.
241. Ryan L, Doody O. The treatment, outcomes and management of hand, wrist, finger, and thumb injuries in the professional/amateur contact sport athletes: A scoping review. Int J Orthop Trauma Nurs. 2024 Aug;54:101108. doi: 10.1016/j.ijotn.2024.101108.	No research design of interest.
242. Sadigursky D, Braid JA, De Lira DNL, Machado BAB, Carneiro RJF, Colavolpe PO. The FIFA 11+ injury prevention program for soccer players: a systematic review. BMC Sports Sci Med Rehabil. 2017 Nov 28;9:18. doi: 10.1186/s13102-017-0083-z.	No meta-analysis of interest.<br><br>Note: No overall meta-analyses or specific subgroups including all our inclusion criteria were found.
243. Sadoghi P, von Keudell A, Vavken P. Effectiveness of anterior cruciate ligament injury prevention training programs. J Bone Joint Surg Am. 2012 May 2;94(9):769-76. doi: 10.2106/JBJS.K.00467.	No meta-analysis of interest.<br><br>Note: Meta-analyses were not performed by type of sport.
244. Salam RA, Arshad A, Das JK, Khan MN, Mahmood W, Freedman SB, Bhutta ZA. Interventions to Prevent Unintentional Injuries Among Adolescents: A Systematic Review and Meta-Analysis. J Adolesc Health. 2016 Oct;59(4S):S76-S87. doi: 10.1016/j.jadohealth.2016.07.024.	No meta-analysis of interest.<br>Note: Other types of injuries rather than musculoskeletal injuries were analyzed (e.g., concussion)
245. Salameh M, Hantouly AT, Rayyan A, Dabbas J, Toubasi AA, Hartnett DA, Blankenhorn B. Return to Play After Isolated Syndesmotic Ligamentous Injury in Athletes: A Systematic Review and Meta-analysis. Foot Ankle Orthop. 2022 May 16;7(2):24730114221096482. doi: 10.1177/24730114221096482.	No meta-analysis of interest.
246. San Martin J, Picabea JM. Propuestas de trabajo en la reducción de lesiones del tren inferior en baloncesto. revisión sistemática. Revista Iberoamericana de Ciencias de la Actividad Física y el Deporte. 2022;11(1):141-153.	No meta-analysis.

247. Sañudo B, Sánchez-Hernández J, Bernardo-Filho M, Abdi E, Taiar R, Núñez J. Integrative Neuromuscular Training in Young Athletes, Injury Prevention, and Performance Optimization: A Systematic Review. Applied Sciences. 2019; 9(18):3839. https://doi.org/10.3390/app9183839	No meta-analysis.
248. Schiftan GS, Ross LA, Hahne AJ. The effectiveness of proprioceptive training in preventing ankle sprains in sporting populations: a systematic review and meta-analysis. J Sci Med Sport. 2015 May;18(3):238-44. doi: 10.1016/j.jsams.2014.04.005.	No meta-analysis of interest.<br><br>Note: Meta-analyses were not performed by type of sport.
249. Schuermans J, Van Hootegem A, Van den Bossche M, Van Gendt M, Witvrouw E, Wezenbeek E. Extended reality in musculoskeletal rehabilitation and injury prevention - A systematic review. Phys Ther Sport. 2022 May;55:229-240. doi: 10.1016/j.ptsp.2022.04.011.	No meta-analysis.
250. Sedaghati P, Alizadeh MH, Shirzad E, Ardjmand A. Review of sport-induced groin injuries. Trauma Mon. 2013 Dec;18(3):107-12. doi: 10.5812/traumamon.12666.	No meta-analysis.
251. Sewry N, Verhagen E, Lambert M, van Mechelen W, Viljoen W, Readhead C, Brown J. Exercise-Based Interventions for Injury Prevention in Tackle Collision Ball Sports: A Systematic Review. Sports Med. 2017 Sep;47(9):1847-1857. doi: 10.1007/s40279-017-0704-4.	No meta-analysis.
252. Shadle IB, Cacolice PA. Eccentric Exercises Reduce Hamstring Strains in Elite Adult Male Soccer Players: A Critically Appraised Topic. J Sport Rehabil. 2017 Nov;26(6):573-577. doi: 10.1123/jsr.2015-0196.	No meta-analysis.
253. Shakked RJ. Lisfranc Injury in the Athlete. JBJS Rev. 2017 Sep;5(9):e4. doi: 10.2106/JBJS.RVW.17.00025.	No objective of this study.
254. Sheth U, Dwyer T, Smith I, Wasserstein D, Theodoropoulos J, Takhar S, Chahal J. Does Platelet-Rich Plasma Lead to Earlier Return to Sport When Compared With Conservative Treatment in Acute Muscle Injuries? A Systematic Review and Meta-analysis. Arthroscopy. 2018 Jan;34(1):281-288.e1. doi: 10.1016/j.arthro.2017.06.039.	No intervention of interest.
255. Shi F, Wu S, Cai W, Zhao Y. Multiple comparisons of the efficacy and safety for six treatments in Acute Achilles Tendon Rupture patients: A systematic review and network meta-analysis. Foot Ankle Surg. 2021 Jul;27(5):468-479. doi: 10.1016/j.fas.2020.07.004.	The focus of the study was not primarily on sports populations.
256. Shreya S, Baliga SD, Baliga SS. Sports-related facial trauma in the Indian population - A systematic review. J Indian Soc Pedod Prev Dent. 2022 Jan-Mar;40(1):3-8. doi: 10.4103/jisppd.jisppd_505_20.	No meta-analysis.
257. Small K, Mc Naughton L, Matthews M. A systematic review into the efficacy of static stretching as part of a warm-up for the prevention of exercise-related injury. Res Sports Med. 2008;16(3):213-31. doi: 10.1080/15438620802310784.	No meta-analysis.
258. Smith TO, Gaukroger A, Metcalfe A, Hing CB. Surgical versus non-surgical interventions for treating patellar dislocation. Cochrane Database Syst Rev. 2023 Jan 24;1(1):CD008106. doi: 10.1002/14651858.CD008106.pub4.	The focus of the study was not primarily on sports populations.
259. Smyth EA, Newman P, Waddington G, Weissensteiner JR, Drew MK. Injury prevention strategies specific to pre-elite athletes competing in Olympic and professional sports - A systematic review. J Sci Med Sport. 2019 Aug;22(8):887-901. doi: 10.1016/j.jsams.2019.03.002.	No meta-analysis.
260. Snyder RA, DeAngelis JP, Koester MC, Spindler KP, Dunn WR. Does shoe insole modification prevent stress fractures? A systematic review. HSS J. 2009 Sep;5(2):92-8. doi: 10.1007/s11420-009-9114-y.	No intervention of interest.
261. Soltanabadi S, Minoonejad H, Bayattork M, Seyedahmadi M. Effect of Virtual Reality and Augmented Reality Training for Injury Prevention and Accelerating	No research design of interest.

Rehabilitation of Anterior Cruciate Ligament Injury in Athletes: A Scoping Review. Asian Journal of Sports Medicine. 2023;14(4):1-19	
262. Soomro N, Sanders R, Hackett D, Hubka T, Ebrahimi S, Freeston J, Cobley S. The Efficacy of Injury Prevention Programs in Adolescent Team Sports: A Meta-analysis. Am J Sports Med. 2016 Sep;44(9):2415-24. doi: 10.1177/0363546515618372.	No meta-analysis of interest.<br><br>Note: No overall meta-analyses or specific subgroups including all our inclusion criteria were found.
263. Startzman AN, Fowler O, Carreira D. Proximal Hamstring Tendinosis and Partial Ruptures. Orthopedics. 2017 Jul 1;40(4):e574-e582. doi: 10.3928/01477447-20170208-05.	No meta-analysis.
264. Stefancin JJ, Parker RD. First-time traumatic patellar dislocation: a systematic review. Clin Orthop Relat Res. 2007 Feb;455:93-101. doi: 10.1097/BLO.0b013e31802eb40a.	No meta-analysis.
265. Steib S, Rahlf AL, Pfeifer K, Zech A. Dose-Response Relationship of Neuromuscular Training for Injury Prevention in Youth Athletes: A Meta-Analysis. Front Physiol. 2017 Nov 14;8:920. doi: 10.3389/fphys.2017.00920.	No meta-analysis of interest.<br><br>Note: Meta-analyses were not performed by type of sport.
266. Stephenson SD, Kocan JW, Vinod AV, Kluczynski MA, Bisson LJ. A Comprehensive Summary of Systematic Reviews on Sports Injury Prevention Strategies. Orthop J Sports Med. 2021 Oct 28;9(10):23259671211035776. doi: 10.1177/23259671211035776.	No research design of interest.
267. Stevenson JH, Beattie CS, Schwartz JB, Busconi BD. Assessing the effectiveness of neuromuscular training programs in reducing the incidence of anterior cruciate ligament injuries in female athletes: a systematic review. Am J Sports Med. 2015 Feb;43(2):482-90. doi: 10.1177/0363546514523388.	No meta-analysis.
268. Stojanovic MD, Ostojic SM. Preventing ACL injuries in team-sport athletes: a systematic review of training interventions. Res Sports Med. 2012 Jul;20(3-4):223-38. doi: 10.1080/15438627.2012.680988.	No meta-analysis.
269. Stoneback JW, Trizno AA, Albright JC. Pediatric and adolescent injury in rodeo. Res Sports Med. 2018;26(sup1):114-128. doi: 10.1080/15438627.2018.1439034.	No meta-analysis.
270. Stovitz SD, Shrier I. Injury rates in team sport events: tackling challenges in assessing exposure time. Br J Sports Med. 2012 Nov;46(14):960-3. doi: 10.1136/bjsports-2011-090693.	No objective of this study.
271. Sugimoto D, Myer GD, Bush HM, Klugman MF, Medina McKeon JM, Hewett TE. Compliance with neuromuscular training and anterior cruciate ligament injury risk reduction in female athletes: a meta-analysis. J Athl Train. 2012 Nov-Dec;47(6):714-23. doi: 10.4085/1062-6050-47.6.10.	No objective of this study.
272. Sugimoto D, Myer GD, Foss KD, Hewett TE. Dosage effects of neuromuscular training intervention to reduce anterior cruciate ligament injuries in female athletes: meta- and sub-group analyses. Sports Med. 2014 Apr;44(4):551-62. doi: 10.1007/s40279-013-0135-9.	No meta-analysis of interest.<br><br>Note: Meta-analyses were not performed by type of sport.

273. Sugimoto D, Myer GD, Foss KD, Hewett TE. Specific exercise effects of preventive neuromuscular training intervention on anterior cruciate ligament injury risk reduction in young females: meta-analysis and subgroup analysis. Br J Sports Med. 2015 Mar;49(5):282-9. doi: 10.1136/bjsports-2014-093461.	No meta-analysis of interest.<br><br>Note: Meta-analyses were not performed by type of sport.
274. Sugimoto D, Myer GD, Barber Foss KD, Pepin MJ, Micheli LJ, Hewett TE. Critical components of neuromuscular training to reduce ACL injury risk in female athletes: meta-regression analysis. Br J Sports Med. 2016 Oct;50(20):1259-1266. doi: 10.1136/bjsports-2015-095596.	No meta-analysis of interest.<br><br>Note: Meta-analyses were not performed by type of sport.
275. Szukics PF, Otlans PT, Arevalo A, Meade M, DeLuca P, Salvo JP. A Scoping Review of Injuries in Amateur and Professional Men's Ice Hockey. Orthop J Sports Med. 2022 Apr 18;10(4):23259671221085968. doi: 10.1177/23259671221085968.	No research design of interest.
276. Taylor JB, Waxman JP, Richter SJ, Shultz SJ. Evaluation of the effectiveness of anterior cruciate ligament injury prevention programme training components: a systematic review and meta-analysis. Br J Sports Med. 2015 Jan;49(2):79-87. doi: 10.1136/bjsports-2013-092358.	No meta-analysis of interest.<br><br>Note: Meta-analyses were not performed by type of sport.
277. Taylor JB, Ford KR, Nguyen AD, Terry LN, Hegedus EJ. Prevention of Lower Extremity Injuries in Basketball: A Systematic Review and Meta-Analysis. Sports Health. 2015 Sep-Oct;7(5):392-8. doi: 10.1177/1941738115593441.	No meta-analysis of interest.<br><br>Note: No overall meta-analyses or specific subgroups including all our inclusion criteria were found.
278. Tedeschi R, Platano D, Giorgi F, Donati D. To Operate or Not? Evaluating the Best Approach for First-Time Patellar Dislocations: A Review. J Clin Med. 2024 Sep 13;13(18):5434. doi: 10.3390/jcm13185434.	No meta-analysis.
279. Thacker SB, Stroup DF, Branche CM, Gilchrist J, Goodman RA, Porter Kelling E. Prevention of knee injuries in sports. A systematic review of the literature. J Sports Med Phys Fitness. 2003 Jun;43(2):165-79.	No meta-analysis.
280. Thacker SB, Gilchrist J, Stroup DF, Kimsey CD Jr. The impact of stretching on sports injury risk: a systematic review of the literature. Med Sci Sports Exerc. 2004 Mar;36(3):371-8. doi: 10.1249/01.mss.0000117134.83018.f7.	No meta-analysis of interest.<br><br>Note: Meta-analyses were not performed by type of sport.
281. Thorborg K, Krommes KK, Esteve E, Clausen MB, Bartels EM, Rathleff MS. Effect of specific exercise-based football injury prevention programmes on the overall injury rate in football: a systematic review and meta-analysis of the FIFA 11 and 11+	Included.

programmes. Br J Sports Med. 2017 Apr;51(7):562-571. doi: 10.1136/bjsports-2016-097066.	
282. Trivedi V, Mishra P, Verma D. Pediatric ACL Injuries: A Review of Current Concepts. Open Orthop J. 2017 Apr 28;11:378-388. doi: 10.2174/1874325001711010378.	No meta-analysis.
283. Tucker R, Raftery M, Verhagen E. Injury risk and a tackle ban in youth Rugby Union: reviewing the evidence and searching for targeted, effective interventions. A critical review. Br J Sports Med. 2016 Aug;50(15):921-5. doi: 10.1136/bjsports-2016-096322.	No objective of this study.
284. Tumiñá-Ospina DM, Rivas-Campo Y, García-Garro PA, Gómez-Rodas A, Afanador-Restrepo DF. Efectividad de los ejercicios nórdicos sobre la incidencia de lesiones de isquiotibiales en futbolistas profesionales y amateur masculinos entre los 15 y 41 años. revisión sistemática. Revista Iberoamericana de Ciencias de la Actividad Física y el Deporte. 2022;11(3):47-65.	No meta-analysis.
285. Valentin S, Linton L, Sculthorpe NF. Effect of supervision and athlete age and sex on exercise-based injury prevention programme effectiveness in sport: A meta-analysis of 44 studies. Res Sports Med. 2024 Sep-Oct;32(5):705-724. doi: 10.1080/15438627.2023.2220059.	No meta-analysis of interest.<br><br>Note: Meta-analyses were not performed by type of sport.
286. van Beijsterveldt AM, van der Horst N, van de Port IG, Backx FJ. How effective are exercise-based injury prevention programmes for soccer players? : A systematic review. Sports Med. 2013 Apr;43(4):257-65. doi: 10.1007/s40279-013-0026-0.	No meta-analysis.
287. van Dyk N, Behan FP, Whiteley R. Including the Nordic hamstring exercise in injury prevention programmes halves the rate of hamstring injuries: a systematic review and meta-analysis of 8459 athletes. Br J Sports Med. 2019 Nov;53(21):1362-1370. doi: 10.1136/bjsports-2018-100045.	No meta-analysis of interest.<br><br>Note: Meta-analyses were not performed by type of sport.
288. van Spanning SH, Verweij LPE, Priester-Vink S, van Deurzen DFP, van den Bekerom MPJ. Operative Versus Nonoperative Treatment Following First-Time Anterior Shoulder Dislocation: A Systematic Review and Meta-Analysis. JBJS Rev. 2021 Sep 23;9(9). doi: 10.2106/JBJS.RVW.20.00232.	The focus of the study was not primarily on sports populations.
289. Vasileiadis I. Injury Prevention Strategies in Football: A Systematic Review. SportMont. 2020;13:109-113.	No meta-analysis.
290. Vatovec R, Kozinc Ž, Šarabon N. Exercise interventions to prevent hamstring injuries in athletes: A systematic review and meta-analysis. Eur J Sport Sci. 2020 Aug;20(7):992-1004. doi: 10.1080/17461391.2019.1689300.	No meta-analysis of interest.<br><br>Note: No overall meta-analyses or specific subgroups including all our inclusion criteria were found.
291. Villaquiran Hurtado AF, Jerez Mayorga DA. Effects of strength training on ankle injuries in soccer players: a systematic review. Retos. 2023;49:657-665.	No meta-analysis.
292. Wagemans J, Bleakley C, Taeymans J, Schurz AP, Kuppens K, Baur H, Vissers D. Exercise-based rehabilitation reduces reinjury following acute lateral ankle sprain: A	No meta-analysis of interest.

systematic review update with meta-analysis. PLoS One. 2022 Feb 8;17(2):e0262023. doi: 10.1371/journal.pone.0262023.	Note: No overall meta-analyses or specific subgroups including all our inclusion criteria were found.
293. Wang S, Lyu B. Are Current Prophylactic Programs Effective in Preventing Patellar Tendinopathy in Athletes and Recruits? A Meta-Analysis and Trial Sequential Analysis. Sports Health. 2023 May;15(3):382-385. doi: 10.1177/19417381221121808.	No meta-analysis of interest.<br><br>Note: Meta-analyses were not performed by type of sport.
294. Warsh JM, Constantin SA, Howard A, Macpherson A. A systematic review of the association between body checking and injury in youth ice hockey. Clin J Sport Med. 2009 Mar;19(2):134-44. doi: 10.1097/JSM.0b013e3181987783.	No intervention of interest.
295. Webster KE, Hewett TE. Meta-analysis of meta-analyses of anterior cruciate ligament injury reduction training programs. J Orthop Res. 2018 Oct;36(10):2696-2708. doi: 10.1002/jor.24043.	No research design of interest.
296. Weldon SM, Hill RH. The efficacy of stretching for prevention of exercise-related injury: a systematic review of the literature. Man Ther. 2003 Aug;8(3):141-50. doi: 10.1016/s1356-689x(03)00010-9.	No meta-analysis.
297. West SW, Shill IJ, Bailey S, Syrydiuk RA, Hayden KA, Palmer D, Black AM, Hagel BE, Stokes KA, Emery CA. Injury Rates, Mechanisms, Risk Factors and Prevention Strategies in Youth Rugby Union: What's All the Ruck-Us About? A Systematic Review and Meta-analysis. Sports Med. 2023 Jul;53(7):1375-1393. doi: 10.1007/s40279-023-01826-z.	No meta-analysis of interest.
298. Windmoller CG. O treinamento proprioceptivo e a prevenção de lesões no esporte. Revista Brasileira de Prescrição e Fisiologia do Exercício. 2013;7(38):131-138.	No meta-analysis.
299. Wright AA, Ness BM, Donaldson M, Hegedus EJ, Salamh P, Cleland JA. Effectiveness of shoulder injury prevention programs in an overhead athletic population: A systematic review. Phys Ther Sport. 2021 Nov;52:189-193. doi: 10.1016/j.ptsp.2021.09.004.	No meta-analysis.
300. Wu H, Brooke-Wavell K, Fong DTP, Paquette MR, Blagrove RC. Do Exercise-Based Prevention Programs Reduce Injury in Endurance Runners? A Systematic Review and Meta-Analysis. Sports Med. 2024 May;54(5):1249-1267. doi: 10.1007/s40279-024-01993-7.	No meta-analysis of interest.<br><br>Note: No overall meta-analyses or specific subgroups including all our inclusion criteria were found.
301. Yang J, Wang Y, Chen J, Yang J, Li N, Wang C, Liao Y. Effects of the "FIFA11+ Kids" Program on Injury Prevention in Children: A Systematic Review and Meta-Analysis. Int J Environ Res Public Health. 2022 Sep 23;19(19):12044. doi: 10.3390/ijerph191912044.	Included.
302. Yang J, Ma F, Wang Q, Cui Y, Zheng J. Effect of blood flow restriction with low-load exercise on muscle damage in healthy adults: A systematic review of randomized	No meta-analysis.

controlled trials. Clin Physiol Funct Imaging. 2024 Jan;44(1):1-13. doi: 10.1111/cpf.12852.	
303. Yeung EW, Yeung SS. A systematic review of interventions to prevent lower limb soft tissue running injuries. Br J Sports Med. 2001 Dec;35(6):383-9. doi: 10.1136/bjsm.35.6.383.	No meta-analysis of interest.
304. Yeung SS, Yeung EW, Gillespie LD. Interventions for preventing lower limb soft-tissue running injuries. Cochrane Database Syst Rev. 2011 Jul 6;(7):CD001256. doi: 10.1002/14651858.CD001256.pub2.	The focus of the study was not primarily on sports populations.
305. Yoo JH, Lim BO, Ha M, Lee SW, Oh SJ, Lee YS, Kim JG. A meta-analysis of the effect of neuromuscular training on the prevention of the anterior cruciate ligament injury in female athletes. Knee Surg Sports Traumatol Arthrosc. 2010 Jun;18(6):824-30. doi: 10.1007/s00167-009-0901-2.	No meta-analysis of interest.<br><br>Note: No overall meta-analyses or specific subgroups including all our inclusion criteria were found.
306. Yu H, Randhawa K, Côté P, Optima Collaboration. The Effectiveness of Physical Agents for Lower-Limb Soft Tissue Injuries: A Systematic Review. J Orthop Sports Phys Ther. 2016 Jul;46(7):523-54. doi: 10.2519/jospt.2016.6521.	No objective of this study.
307. Yuan Haoxiang, Xu Jing, Zeng Jinshu, Chen Hao, Yan Yelei, Chen Jiahao, Liu Qingshan, Xu Fei. Sequence of prevention for anterior cruciate ligament injury: screening, intervention and assessment[J]. Chinese Journal of Tissue Engineering Research, 2022, 26(17): 2775-2781.	No access at full text.
308. Zech A, Hübscher M, Vogt L, Banzer W, Hänsel F, Pfeifer K. Neuromuscular training for rehabilitation of sports injuries: a systematic review. Med Sci Sports Exerc. 2009 Oct;41(10):1831-41. doi: 10.1249/MSS.0b013e3181a3cf0d.	No meta-analysis.
309. Zech A, Hübscher M. Sensomotorisches Training zur Prävention von Sprunggelenksverletzungen. Deutsche Zeitschrift für Sportmedizin. 2012;63(1):5-8.	No research design of interest.<br>Note: This study has not the structure of a systematic review.
310. Zhai H, Li C, Xia J, Wei H, Qin S. Integrative neuromuscular training for injury prevention of lower extremity in athletes: a meta-analysis. Chinese Journal of Tissue Engineering Research. 2022;26 (15):2454-2460.doi: 10.12307/2022.606.	No meta-analysis of interest.<br><br>Note: We did not have access to the full text, but the abstract specified the subgroup analyses, and no meta-analyses by sports were performed.
311. Zhang YJ, Long X, Du JY, Wang Q, Lin XJ. Is Early Controlled Motion and Weightbearing Recommended for Nonoperatively Treated Acute Achilles Tendon	The focus of the study was not primarily on

Rupture? A Systematic Review and Meta-analysis. Orthop J Sports Med. 2021 Sep 21;9(9):23259671211024605. doi: 10.1177/23259671211024605.	sports populations.
312. Zhang H, Jiang Q, Li A. The impact of resistance-based training programs on throwing performance and throwing-related injuries in baseball players: A systematic review. Heliyon. 2023 Nov 29;9(12):e22797. doi: 10.1016/j.heliyon.2023.e22797.	No meta-analysis.
313. Zhang ZX, Lai J, Shen L, Krishna L. Effectiveness of exercise-based sports injury prevention programmes in reducing injury rates in adolescents and their implementation in the community: a mixed-methods systematic review. Br J Sports Med. 2024 May 31;58(12):674-684. doi: 10.1136/bjsports-2023-107717.	No meta-analysis of interest.<br><br>Note: Meta-analyses were not performed by type of sport.
314. Zhao HM, Yu GR, Yang YF, Zhou JQ, Aubeeluck A. Outcomes and complications of operative versus non-operative treatment of acute Achilles tendon rupture: a meta-analysis. Chin Med J (Engl). 2011 Dec;124(23):4050-5.	The focus of the study was not primarily on sports populations.
315. Zhou K, Song L, Zhang P, Wang C, Wang W. Surgical Versus Non-Surgical Methods for Acute Achilles Tendon Rupture: A Meta-Analysis of Randomized Controlled Trials. J Foot Ankle Surg. 2018 Nov-Dec;57(6):1191-1199. doi: 10.1053/j.jfas.2018.05.007.	The focus of the study was not primarily on sports populations.
