# Supplementary material for: Protecting Athletes: The Clinical Relevance of Meta-Analyses on Injury Prevention Programs for Sports and Musculoskeletal Body Regions: An Overview of Systematic Reviews with Meta-Analyses of Randomized Clinical Trials
Source: Healthcare (Basel). 2025 Jun 27;13(13):1530. doi: 10.3390/healthcare13131530 (PMC12250077; doi:10.3390/healthcare13131530)
Supplement: Supplementary file 1 [file healthcare-13-01530-s001.zip › Suppl File S4 Analysis manual searches.pdf]

Supplementary file S4. Articles retrieved from manual searches.

No meta-analysis (k=17)

No meta-analysis of interest (k=3)

No research design of interest (k=1)

The focus of the study was not primarily on sports populations (k=4)

	Excluded reviews	Reason	Article retrieved from
1.	Silfies SP, Ebaugh D, Pontillo M, Butowicz CM. Critical review of the impact of core stability on upper extremity athletic injury and performance. Braz J Phys Ther. 2015 Sep-Oct;19(5):360-8. doi: 10.1590/bjpt-rbf.2014.0108.	No meta-analysis.	Azócar-Gallardo J, Azócar-Gallardo Y, Ojeda-Aravena A, Cárdenas-Mansill R, Montecinos-Zuñiga J. Efectividad del entrenamiento de la musculatura del Core en la prevención de lesiones de hombro en deportes con lanzamientos sobre la cabeza: una revisión sistemática. Rev Andal Med Deporte. 2021;14(2):120-124.
2.	Olsen L, Scanlan A, MacKay M, Babul S, Reid D, Clark M, Raina P. Strategies for prevention of soccer related injuries: a systematic review. Br J Sports Med. 2004 Feb;38(1):89-94. doi: 10.1136/bjsm.2002.003079.	No meta-analysis.	Barrera J, Figueiredo AJ, Clemente FM, Field A, Valenzuela L, Sarmento H. Injury Prevention Programmes in Male Soccer Players: An Umbrella Review of Systematic Reviews. Journal of Men's Health. 2022;18(10):1-17.
3.	Lauersen JB, Bertelsen DM, Andersen LB. The effectiveness of exercise interventions to prevent sports injuries: a systematic review and meta-analysis of randomised controlled trials. Br J Sports Med.	No meta-analysis of interest.<br>Note: Meta-analyses were not performed by type of sport.	Brunner R, Friesenbichler B, Casartelli NC, Bizzini M, Maffiuletti NA, Niedermann K. Effectiveness of multicomponent lower

	2014 Jun;48(11):871-7. doi: 10.1136/bjsports-2013-092538.		extremity injury prevention programmes in team-sport athletes: an umbrella review. Br J Sports Med. 2019 Mar;53(5):282-288. doi: 10.1136/bjsports-2017-098944.
4.	Grindstaff TL, Hammill RR, Tuzson AE, Hertel J. Neuromuscular control training programs and noncontact anterior cruciate ligament injury rates in female athletes: a numbers-needed-to-treat analysis. J Athl Train. 2006 Oct-Dec;41(4):450-6.	No meta-analysis of interest.<br>Note: Meta-analyses were not performed by type of sport.	Brunner R, Friesenbichler B, Casartelli NC, Bizzini M, Maffiuletti NA, Niedermann K. Effectiveness of multicomponent lower extremity injury prevention programmes in team-sport athletes: an umbrella review. Br J Sports Med. 2019 Mar;53(5):282-288. doi: 10.1136/bjsports-2017-098944.
5.	Sugimoto D, Myer GD, McKeon JM, Hewett TE. Evaluation of the effectiveness of neuromuscular training to reduce anterior cruciate ligament injury in female athletes: a critical review of relative risk reduction and numbers-needed-to-treat analyses. Br J Sports Med. 2012 Nov;46(14):979-88. doi: 10.1136/bjsports-2011-090895.	No meta-analysis.	Brunner R, Friesenbichler B, Casartelli NC, Bizzini M, Maffiuletti NA, Niedermann K. Effectiveness of multicomponent lower extremity injury prevention programmes in team-sport athletes: an umbrella review. Br J Sports Med. 2019 Mar;53(5):282-288. doi: 10.1136/bjsports-2017-098944.

6.	Donnell-Fink LA, Klara K, Collins JE, Yang HY, Goczalk MG, Katz JN, Losina E. Effectiveness of Knee Injury and Anterior Cruciate Ligament Tear Prevention Programs: A Meta-Analysis. PLoS One. 2015 Dec 4;10(12):e0144063. doi: 10.1371/journal.pone.0144063.	The focus of the study was not primarily on sports populations.<br><br>Note: One included study was based on a military population, and no subgroup analysis focused on sports populations were detected.	Dargo L, Robinson KJ, Games KE. Prevention of Knee and Anterior Cruciate Ligament Injuries Through the Use of Neuromuscular and Proprioceptive Training: An Evidence-Based Review. J Athl Train. 2017 Dec;52(12):1171-1172. doi: 10.4085/1062-6050-52.12.21.
7.	Ishøi L, Krommes K, Husted RS, Juhl CB, Thorborg K. Diagnosis, prevention and treatment of common lower extremity muscle injuries in sport - grading the evidence: a statement paper commissioned by the Danish Society of Sports Physical Therapy (DSSF). Br J Sports Med. 2020 May;54(9):528-537. doi: 10.1136/bjsports-2019-101228.	No meta-analysis of interest.<br><br>Note: No overall meta-analyses or specific subgroups including all our inclusion criteria were found.<br><br>The authors did not specify in their analyses that subgroups by type of sport were performed.	Ishøi L, Krommes K, Husted RS, Juhl CB, Virgile A, Thorborg K. Infographic. Diagnosis, prevention and treatment of common lower extremity muscle injuries in sport-grading the evidence: a statement paper commissioned by the Danish Society of Sports Physical Therapy (DSSF). Br J Sports Med. 2020 Sep;54(18):1116-1117. doi: 10.1136/bjsports-2020-102119.
8.	Padua, D.A.; Marshall, S.W. Evidence Supporting ACL-Injury-Prevention Exercise Programs: A Review of the Literature. Int. J. Athl. Ther. Train. 2006, 11, 11–23.	No meta-analysis.	Mattu AT, Ghali B, Linton V, Zheng A, Pike I. Prevention of Non-Contact Anterior Cruciate Ligament Injuries among Youth Female Athletes: An Umbrella Review. Int J Environ Res Public Health. 2022 Apr 12;19(8):4648. doi: 10.3390/ijerph19084648.

9.	Bhandari M, Guyatt GH, Siddiqui F, Morrow F, Busse J, Leighton RK, Sprague S, Schemitsch EH. Treatment of acute Achilles tendon ruptures: a systematic overview and metaanalysis. Clin Orthop Relat Res. 2002 Jul;(400):190-200. doi: 10.1097/00003086-200207000-00024.	The focus of the study was not primarily on sports populations.<br><br>Note: This study include sports populations, but the review was not focused on that.	McCormack RG. Surgery or conservative treatment for acute achilles tendon ruptures: a meta-analysis. Clin J Sport Med. 2003 May;13(3):194. doi: 10.1097/00042752-200305000-00016.
10.	Meulenkamp B, Woolnough T, Cheng W, Shorr R, Stacey D, Richards M, Gupta A, Fergusson D, Graham ID. What Is the Best Evidence to Guide Management of Acute Achilles Tendon Ruptures? A Systematic Review and Network Meta-Analysis of Randomized Controlled Trials. Clin Orthop Relat Res. 2021 Oct 1;479(10):2119-2131. doi: 10.1097/CORR.0000000000001861.	The focus of the study was not primarily on sports populations.	Meulenkamp B, Stacey D, Fergusson D, Hutton B, Mlis RS, Graham ID. Protocol for treatment of Achilles tendon ruptures; a systematic review with network meta-analysis. Syst Rev. 2018 Dec 23;7(1):247. doi: 10.1186/s13643-018-0912-5.
11.	Behm DG, Blazevich AJ, Kay AD, McHugh M. Acute effects of muscle stretching on physical performance, range of motion, and injury incidence in healthy active individuals: a systematic review. Appl Physiol Nutr Metab. 2016 Jan;41(1):1-11. doi: 10.1139/apnm-2015-0235.	No meta-analysis.	Stephenson SD, Kocan JW, Vinod AV, Kluczynski MA, Bisson LJ. A Comprehensive Summary of Systematic Reviews on Sports Injury Prevention Strategies. Orthop J Sports Med. 2021 Oct 28;9(10):23259671211035776. doi: 10.1177/23259671211035776.
12.	Brachman A, Kamieniarz A, Michalska J, Pawłowski M, Słomka KJ, Juras G. Balance Training Programs in Athletes - a Systematic Review. J Hum Kinet. 2017 Aug 1;58:45-64. doi: 10.1515/hukin-2017-0088.	No meta-analysis.	Stephenson SD, Kocan JW, Vinod AV, Kluczynski MA, Bisson LJ. A Comprehensive Summary of Systematic Reviews on Sports Injury Prevention Strategies. Orthop J Sports Med. 2021 Oct

			28;9(10):23259671211035776. doi: 10.1177/23259671211035776.
13.	Hincapié CA, Morton EJ, Cassidy JD. Musculoskeletal injuries and pain in dancers: a systematic review. Arch Phys Med Rehabil. 2008 Sep;89(9):1819-29. doi: 10.1016/j.apmr.2008.02.020.	No meta-analysis.	Stephenson SD, Kocan JW, Vinod AV, Kluczynski MA, Bisson LJ. A Comprehensive Summary of Systematic Reviews on Sports Injury Prevention Strategies. Orthop J Sports Med. 2021 Oct 28;9(10):23259671211035776. doi: 10.1177/23259671211035776.
14.	Woollings KY, McKay CD, Emery CA. Risk factors for injury in sport climbing and bouldering: a systematic review of the literature. Br J Sports Med. 2015 Sep;49(17):1094-9. doi: 10.1136/bjsports-2014-094372.	No meta-analysis.	Stephenson SD, Kocan JW, Vinod AV, Kluczynski MA, Bisson LJ. A Comprehensive Summary of Systematic Reviews on Sports Injury Prevention Strategies. Orthop J Sports Med. 2021 Oct 28;9(10):23259671211035776. doi: 10.1177/23259671211035776.
15.	Abernethy L, Bleakley C. Strategies to prevent injury in adolescent sport: a systematic review. Br J Sports Med. 2007 Oct;41(10):627-38. doi: 10.1136/bjsm.2007.035691.	No meta-analysis.	Stephenson SD, Kocan JW, Vinod AV, Kluczynski MA, Bisson LJ. A Comprehensive Summary of Systematic Reviews on Sports Injury Prevention Strategies. Orthop J Sports Med. 2021 Oct 28;9(10):23259671211035776. doi:

			10.1177/23259671211035776.
16.	Paszkewicz J, Webb T, Waters B, Welch McCarty C, Van Lunen B. The effectiveness of injury-prevention programs in reducing the incidence of anterior cruciate ligament sprains in adolescent athletes. J Sport Rehabil. 2012 Nov;21(4):371-7. doi: 10.1123/jsr.21.4.371.	No meta-analysis.	Stephenson SD, Kocan JW, Vinod AV, Kluczynski MA, Bisson LJ. A Comprehensive Summary of Systematic Reviews on Sports Injury Prevention Strategies. Orthop J Sports Med. 2021 Oct 28;9(10):23259671211035776. doi: 10.1177/23259671211035776.
17.	Chang, W.-D., & Lai, P.-T. (2014). Neuromuscular Training for Prevention of Anterior Cruciate Ligament Injury in Female Athletes. International Journal of Athletic Therapy and Training, 19(6), 17–21. doi:10.1123/ijatt.2014-0042	No meta-analysis.	Stephenson SD, Kocan JW, Vinod AV, Kluczynski MA, Bisson LJ. A Comprehensive Summary of Systematic Reviews on Sports Injury Prevention Strategies. Orthop J Sports Med. 2021 Oct 28;9(10):23259671211035776. doi: 10.1177/23259671211035776.
18.	Thacker SB, Gilchrist J, Stroup DF, Kimsey CD. The prevention of shin splints in sports: a systematic review of literature. Med Sci Sports Exerc. 2002 Jan;34(1):32-40. doi: 10.1097/00005768-200201000-00006.	No meta-analysis.	Stephenson SD, Kocan JW, Vinod AV, Kluczynski MA, Bisson LJ. A Comprehensive Summary of Systematic Reviews on Sports Injury Prevention Strategies. Orthop J Sports Med. 2021 Oct 28;9(10):23259671211035776. doi: 10.1177/23259671211035776.

19.	Michalis AH, Stergioulas A. Hamstring strains in football: prevention and rehabilitation rules. Systematic review. Biol Exerc. 2016;12(1): 121-148	No meta-analysis.	Stephenson SD, Kocan JW, Vinod AV, Kluczynski MA, Bisson LJ. A Comprehensive Summary of Systematic Reviews on Sports Injury Prevention Strategies. Orthop J Sports Med. 2021 Oct 28;9(10):23259671211035776. doi: 10.1177/23259671211035776.
20.	McKeon PO, Hertel J. Systematic review of postural control and lateral ankle instability, part II: is balance training clinically effective? J Athl Train. 2008 May-Jun;43(3):305-15. doi: 10.4085/1062-6050-43.3.305.	No meta-analysis.	Stephenson SD, Kocan JW, Vinod AV, Kluczynski MA, Bisson LJ. A Comprehensive Summary of Systematic Reviews on Sports Injury Prevention Strategies. Orthop J Sports Med. 2021 Oct 28;9(10):23259671211035776. doi: 10.1177/23259671211035776.
21.	Doherty C, Bleakley C, Delahunt E, Holden S. Treatment and prevention of acute and recurrent ankle sprain: an overview of systematic reviews with meta-analysis. Br J Sports Med. 2017 Jan;51(2):113-125. doi: 10.1136/bjsports-2016-096178.	No research design of interest.	Stephenson SD, Kocan JW, Vinod AV, Kluczynski MA, Bisson LJ. A Comprehensive Summary of Systematic Reviews on Sports Injury Prevention Strategies. Orthop J Sports Med. 2021 Oct 28;9(10):23259671211035776. doi: 10.1177/23259671211035776.
22.	Bleakley CM, McDonough SM, MacAuley DC. Some conservative strategies are effective when added to	No meta-analysis.	Doherty C, Bleakley C, Delahunt E, Holden S. Treatment and prevention

	controlled mobilisation with external support after acute ankle sprain: a systematic review. Aust J Physiother. 2008;54(1):7-20. doi: 10.1016/s0004-9514(08)70061-8.		of acute and recurrent ankle sprain: an overview of systematic reviews with meta-analysis. Br J Sports Med. 2017 Jan;51(2):113-125. doi: 10.1136/bjsports-2016-096178.
23.	Loudon JK, Santos MJ, Franks L, Liu W. The effectiveness of active exercise as an intervention for functional ankle instability: a systematic review. Sports Med. 2008;38(7):553-63. doi: 10.2165/00007256-200838070-00003.	No meta-analysis.	Doherty C, Bleakley C, Delahunt E, Holden S. Treatment and prevention of acute and recurrent ankle sprain: an overview of systematic reviews with meta-analysis. Br J Sports Med. 2017 Jan;51(2):113-125. doi: 10.1136/bjsports-2016-096178.
24.	van der Wees PJ, Lenssen AF, Hendriks EJ, Stomp DJ, Dekker J, de Bie RA. Effectiveness of exercise therapy and manual mobilisation in ankle sprain and functional instability: a systematic review. Aust J Physiother. 2006;52(1):27-37. doi: 10.1016/s0004-9514(06)70059-9.	The focus of the study was not primarily on sports populations.	Doherty C, Bleakley C, Delahunt E, Holden S. Treatment and prevention of acute and recurrent ankle sprain: an overview of systematic reviews with meta-analysis. Br J Sports Med. 2017 Jan;51(2):113-125. doi: 10.1136/bjsports-2016-096178.
25.	van Ochten JM, van Middelkoop M, Meuffels D, Bierma-Zeinstra SM. Chronic complaints after ankle sprains: a systematic review on effectiveness of treatments. J Orthop Sports Phys Ther. 2014 Nov;44(11):862-71, C1-23. doi: 10.2519/jospt.2014.5221.	No meta-analysis.	Doherty C, Bleakley C, Delahunt E, Holden S. Treatment and prevention of acute and recurrent ankle sprain: an overview of systematic reviews with meta-analysis. Br J Sports Med. 2017 Jan;51(2):113-125. doi: 10.1136/bjsports-2016-096178.
