# Supplementary material for: Protecting Athletes: The Clinical Relevance of Meta-Analyses on Injury Prevention Programs for Sports and Musculoskeletal Body Regions: An Overview of Systematic Reviews with Meta-Analyses of Randomized Clinical Trials
Source: Healthcare (Basel). 2025 Jun 27;13(13):1530. doi: 10.3390/healthcare13131530 (PMC12250077; doi:10.3390/healthcare13131530)
Supplement: Supplementary file 1 [file healthcare-13-01530-s001.zip › Suppl File S5 Overlap ACL.pdf]

Supplementary file S5. Matrices of evidence and the corrected covered area (CCA) calculations for meta-analyses evaluating anterior cruciate ligament injuries. Note: The following reviews were not included in the overlap calculation because they did not specify the studies that were included in the meta-analysis.

$$CCA = \frac{N-r}{rc-r} = \frac{13-8}{24-8} = \frac{5}{16} = 0.3125 = 31\%$$

Note: N is the total number of original studies (including duplicates) in the meta-analyses of interest (the sum of all checked boxes in the citation matrix). Furthermore, r is the number of original studies without accounting for duplicates. Finally, c is the number of systematic reviews included in the evidence matrix (k=3). CCA = corrected covered area.

**Number of studies without accounting for duplicates**	**Primary research (references)**	**Systematic reviews where primary research appear including primary research duplicates**
1.	Al Attar WSA, Soomro N, Pappas E, Sinclair PJ, Sanders RH. Adding a post-training FIFA 11+ exercise program to the pre-training FIFA 11+ injury prevention program reduces injury rates among male amateur soccer players: A clusterrandomised trial. J Physiother 2017; 63: 235-42.	1. Al Attar et al. 2022

2.	Gilchrist J, Mandelbaum BR, Melancon H, et al. A randomized controlled trial to prevent noncontact anterior cruciate ligament injury in female collegiate soccer players. Am J Sports Med 2008;36:1476–83.	2. Crossley et al. 2020<br>3. Grimm et al. 2015
3.	Heidt RS, Sweeterman LM, Carlonas RL, et al. Avoidance of soccer injuries with preseason conditioning. Am J Sports Med 2000;28:659–62.	4. Crossley et al. 2020
4.	LaBella CR, Huxford MR, Grissom J, et al. Effect of neuromuscular warm-up on injuries in female soccer and Basketball athletes in urban public high schools. Arch Pediatr Adolesc Med 2011;165:1033–40.	5. Crossley et al. 2020
5.	Silvers-Granelli H, Mandelbaum B, Adeniji O, Insler S, Bizzini M, Pohlig R, et al. Efficacy of the FIFA 11+ injury prevention program in the collegiate male soccer player. Am J Sports Med 2015; 43: 2628-37.	6. Al Attar et al. 2022
6.	Soderman K, Werner S, Pietila T, Engstrom B, Alfredson H. Balance board training: prevention of traumatic injuries of the lower extremities in female soccer players? A prospective randomized intervention study. Knee Surg Sports Traumatol Arthrosc. 2000;8(6):356- 363	7. Grimm et al. 2015
7.	Steffen K, Myklebust G, Olsen OE, Holme I, Bahr R. Preventing injuries in female youth football – a cluster-randomized controlled trial. Scand J Med Sci Sports 2008; 18: 605-14.	8. Al Attar et al. 2022<br>9. Crossley et al. 2020<br>10. Grimm et al. 2015
8.	Waldén M, Atroshi I, Magnusson H, Wagner P, Hägglund M. Prevention of acute knee injuries in adolescent female football players: Cluster randomised controlled trial. BMJ 2012; 344: e3042.	11. Al Attar et al. 2022<br>12. Crossley et al. 2020<br>13. Grimm et al. 2015
