# Supplementary material for: Protecting Athletes: The Clinical Relevance of Meta-Analyses on Injury Prevention Programs for Sports and Musculoskeletal Body Regions: An Overview of Systematic Reviews with Meta-Analyses of Randomized Clinical Trials
Source: Healthcare (Basel). 2025 Jun 27;13(13):1530. doi: 10.3390/healthcare13131530 (PMC12250077; doi:10.3390/healthcare13131530)
Supplement: Supplementary file 1 [file healthcare-13-01530-s001.zip › Suppl File S6 Overlap knee injuries.pdf]

Supplementary file S6. Matrices of evidence and the corrected covered area (CCA) calculations for meta-analyses evaluating knee injuries.

Note: The following reviews were not included in the overlap calculation because they did not specify the studies that were included in the meta-analysis.

$$CCA = \frac{N-r}{rc-r} = \frac{55-23}{161-23} = \frac{32}{138} = 0.2318 = 23\%$$

Note: N is the total number of original studies (including duplicates) in the meta-analyses of interest (the sum of all checked boxes in the citation matrix). Furthermore, r is the number of original studies without accounting for duplicates. Finally, c is the number of systematic reviews included in the evidence matrix (k=7). CCA = corrected covered area.

Number of studies without accounting for duplicates	Primary research (references)	Systematic reviews where primary research appear including primary research duplicates
1.	Al Attar WSA, Soomro N, Pappas E, Sinclair PJ, Sanders RH. Adding a post-training FIFA 11+ exercise program to the pre-training FIFA 11+ injury prevention program reduces injury rates among male amateur soccer players: A clusterrandomised trial. J Physiother 2017; 63: 235-42.	1. Al Attar et al. 2022<br>2. Al Attar et al. 2023

2.	Al Attar, W.S.A.; Bizzini, M.; Alzahrani, H.; Alarifi, S.; Ghulam, H.; Alyami, M.; Alzhrani, M.; Sanders, R.H. The FIFA11+ Kids Injury Prevention Program Reduces Injury Rates Among Male Children Soccer Players: A Clustered Randomized Controlled Trial. Sports Health 2022, 1941738122110922	3. Yang et al. 2022
3.	Barber Foss KD, Thomas S, Khoury JC, et al. A school-based neuromuscular training program and sport-related injury incidence: a prospective randomized controlled clinical trial. J Athl Train 2018;53:20–8.	4. Crossley et al. 2020
4.	Ekstrand J, Gillquist J, Liljedahl SO. Prevention of soccer injuries: supervision by doctor and physiotherapist. Am J Sports Med. 1983;11(3):116-120.	5. Grimm et al. 2015
5.	Emery CA, Meeuwisse WH. The effectiveness of a neuromuscular prevention strategy to reduce injuries in youth soccer: A cluster-randomised controlled trial. Br J Sports Med 2010; 44: 555-62.	6. Al Attar et al. 2022<br>7. Al Attar et al. 2023<br>8. Crossley et al. 2020<br>9. Grimm et al. 2015<br>10. Obërtinca et al. 2023
6.	Engebretsen AH, Myklebust G, Holme I, Engebretsen L, Bahr R. Prevention of injuries among male soccer players: a prospective, randomized intervention study targeting players with previous injuries or reduced function. Am J Sports Med. 2008;36(6):1052-1060.	11. Grimm et al. 2015
7.	Finch CF, Twomey DM, Fortington LV, Doyle TL, Elliott BC, Akram M, et al. Preventing Australian football injuries with a targeted neuromuscular control exercise programme: comparative injury rates from a training intervention delivered in a clustered randomised controlled trial. Inj Prev. 2016;22(2):123–8. https:// doi.org/10.1136/injuryprev-2015-041667	12. Obërtinca et al. 2023
8.	Gilchrist J, Mandelbaum BR, Melancon H, et al. A randomized controlled trial to prevent noncontact anterior cruciate ligament injury in female collegiate soccer players. Am J Sports Med 2008;36:1476–83.	13. Crossley et al. 2020<br>14. Grimm et al. 2015<br>15. Obërtinca et al. 2023

9.	Hammes D, aus der Fünten K, Kaiser S, Frisen E, Bizzini M, Meyer T. Injury prevention in male veteran football players – a randomised controlled trial using “FIFA 11+”. J Sports Sci 2014; 33: 873-81	16. Al Attar et al. 2022<br>17. Al Attar et al. 2023<br>18. Obërtinca et al. 2023<br>19. Thorborg et al. 2017
10.	Heidt RS, Sweeterman LM, Carlonas RL, et al. Avoidance of soccer injuries with preseason conditioning. Am J Sports Med 2000;28:659–62.	20. Crossley et al. 2020
11.	LaBella CR, Huxford MR, Grissom J, et al. Effect of neuromuscular warm-up on injuries in female soccer and Basketball athletes in urban public high schools. Arch Pediatr Adolesc Med 2011;165:1033–40.	21. Crossley et al. 2020
12.	Ma, Y. Application Research of FIFA11+ Kids in Primary School Physical Education Class; RCT, Tianjin Normal University: Tianjin, China, 2019.	22. Yang et al. 2022
13.	Nuhu A, Jelsma J, Dunleavy K, Burgess T. Efect of the FIFA 11 + soccer specifc warm up programme on the incidence of injuries: a cluster-randomised controlled trial. PLoS ONE. 2021;16(5): e0251839. https://doi.org/10.1371/journal.pone.0251839	23. Obërtinca et al. 2023
14.	Owoeye OB, Akinbo SRA, Tella BA, Olawale OA. Efficacy of the FIFA 11+ warm-up program in male youth football: A cluster randomised controlled trial. J Sports Sci Med 2014; 13: 321-8.	24. Al Attar et al. 2022<br>25. Al Attar et al. 2023<br>26. Obërtinca et al. 2023<br>27. Thorborg et al. 2017
15.	Rössler R, Junge A, Bizzini M, et al. A multinational cluster randomised controlled trial to assess the efficacy of ’11+ Kids’: a warm-up programme to prevent injuries in children’s football. Sports Med 2018;48:1493–504.	28. Crossley et al. 2020<br>29. Obërtinca et al. 2023<br>30. Yang et al. 2022
16.	Silvers-Granelli H, Mandelbaum B, Adeniji O, Insler S, Bizzini M, Pohlig R, et al. Efficacy of the FIFA 11+ injury prevention program in the collegiate male soccer player. Am J Sports Med 2015; 43: 2628-37.	31. Al Attar et al. 2022<br>32. Al Attar et al. 2023<br>33. Thorborg et al. 2017

17.	Silvers-Granelli HJ, Bizzini M, Arundale A, Mandelbaum BR, Snyder-Mackler L. Does the FIFA 11 + injury prevention program reduce the incidence of ACL injury in male soccer players? Clin Orthop Relat Res. 2017;475(10):2447–55. https://doi.org/10. 1007/s11999-017-5342-5.	34. Obërtinca et al. 2023
18.	Soderman K, Werner S, Pietila T, Engstrom B, Alfredson H. Balance board training: prevention of traumatic injuries of the lower extremities in female soccer players? A prospective randomized intervention study. Knee Surg Sports Traumatol Arthrosc. 2000;8(6):356- 363.	35. Grimm et al. 2015
19.	Soligard T, Myklebust G, Steffen K, Holme I, Silvers H, Bizzini M, et al. Comprehensive warm-up programme to prevent injuries in young female footballers: Cluster randomised controlled trial. BMJ 2008; 337: a2469.	36. Al Attar et al. 2022<br>37. Al Attar et al. 2023<br>38. Crossley et al. 2020<br>39. Grimm et al. 2015<br>40. Obërtinca et al. 2023<br>41. Thorborg et al. 2017
20.	Steffen K, Myklebust G, Olsen OE, Holme I, Bahr R. Preventing injuries in female youth football – a cluster-randomized controlled trial. Scand J Med Sci Sports 2008; 18: 605-14.	42. Al Attar et al. 2022<br>43. Al Attar et al. 2022<br>44. Crossley et al. 2020<br>45. Grimm et al. 2015<br>46. Obërtinca et al. 2023
21.	van Beijsterveldt AMC, van de Port IGL, Krist MR, Schmikli SL, Stubbe JH, Frederiks JE, et al. Effectiveness of an injury prevention programme for adult male amateur soccer players: A cluster-randomised controlled trial. Br J Sports Med 2012; 46: 1114-8.	47. Al Attar et al. 2022<br>48. Al Attar et al. 2023<br>49. Grimm et al. 2015
22.	Waldén M, Atroshi I, Magnusson H, Wagner P, Hägglund M. Prevention of acute knee injuries in adolescent female football players: Cluster randomised controlled trial. BMJ 2012; 344: e3042.	50. Al Attar et al. 2022<br>51. Al Attar et al. 2023<br>52. Crossley et al. 2020<br>53. Grimm et al. 2015<br>54. Obërtinca et al. 2023

23.	Zarei, M.; Abbasi, H.; Namazi, P.; Asgari, M.; Rommers, N.; Rössler, R. The 11+ Kids warm-up programme to prevent injuries in young Iranian male high-level football (soccer) players: A cluster-randomised controlled trial. J. Sci. Med. Sport 2020, 23, 469–474.	55. Yang et al. 2022
