# Supplementary material for: Protecting Athletes: The Clinical Relevance of Meta-Analyses on Injury Prevention Programs for Sports and Musculoskeletal Body Regions: An Overview of Systematic Reviews with Meta-Analyses of Randomized Clinical Trials
Source: Healthcare (Basel). 2025 Jun 27;13(13):1530. doi: 10.3390/healthcare13131530 (PMC12250077; doi:10.3390/healthcare13131530)
Supplement: Supplementary file 1 [file healthcare-13-01530-s001.zip › Suppl File S7 Overlap hamstring injuries.pdf]

Supplementary file S7. Matrices of evidence and the corrected covered area (CCA) calculations for meta-analyses evaluating hamstring injuries.

Note: The following reviews were not included in the overlap calculation because they did not specify the studies that were included in the meta-analysis.

- Ripley et al. 2021

$$CCA = \frac{N-r}{rc-r} = \frac{29-19}{114-19} \quad \frac{10}{95} = 0.1052 = 10\%$$

Note: N is the total number of original studies (including duplicates) in the meta-analyses of interest (the sum of all checked boxes in the citation matrix). Furthermore, r is the number of original studies without accounting for duplicates. Finally, c is the number of systematic reviews included in the evidence matrix (k=6). CCA = corrected covered area.

**Number of studies without accounting for duplicates**	**Primary research (references)**	**Systematic reviews where primary research appear including primary research duplicates**
1.	Al Attar WSA, Soomro N, Pappas E, Sinclair PJ, Sanders RH. Adding a post-training FIFA 11+ exercise program to the pre-training FIFA 11+ injury prevention program reduces injury rates among male amateur soccer players: a cluster-randomised trial. J Physiother. 2017;63(4):235-242.	1.<br>2. Al Attar et al. 2023 B

2.	Cadu, J. P., Goreau, V., & Lacourpaille, L. (2022). A Very Low Volume of Nordic Hamstring Exercise Increases Maximal Eccentric Strength and Reduces Hamstring Injury Rate in Professional Soccer Players. J Sport Rehabil, 31(8), 1061-1066. doi:10.1123/jsr.2021-0445	3. Avila-Quintero et al. 2024
3.	Chebbi, S., Chamari, K., Van Dyk, N., Gabbett, T., & Tabben, M. (2022). Hamstring Injury Prevention for Elite Soccer Players: A Real-World Prevention Program Showing the Effect of Players' Compliance on the Outcome. J Strength Cond Res, 36(5), 1383- 1388. doi:10.1519/jsc.0000000000003505	4. Avila-Quintero et al. 2024
4.	Hasebe, Y., Akasaka, K., Otsudo, T., Tachibana, Y., Hall, T., & Yamamoto, M. (2020). Effects of Nordic Hamstring Exercise on Hamstring Injuries in High School Soccer Players: A Randomized Controlled Trial. Int J Sports Med, 41(3), 154-160. doi:10.1055/a-1034-7854	5. Avila-Quintero et al. 2024
5.	Elerian, A. E., El-Sayyad, M. M., & Dorgham, H. A. A. (2019). Effect of Pre-training and Post-training Nordic Exercise on Hamstring Injury Prevention, Recurrence, and Severity in Soccer Players. Ann Rehabil Med, 43(4), 465-473. doi:10.5535/arm.2019.43.4.465	6. Avila-Quintero et al. 2024
6.	Engebretsen AH, Myklebust G, Holme I, Engebretsen L, Bahr R. Prevention of injuries among male soccer players: a prospective, randomized intervention study targeting players with previous injuries or reduced function. Am J Sports Med. 2008;36(6):1052-1060.	7. Al Attar et al. 2023 B
7.	Espinosa, G., Pöyhönen, T., Aramendi, J., Samaniego, J., Emparanza, J., & Kyröläinen, H. (2015). Effects of an eccentric training programme on hamstring strain injuries in women football players. Biomedical Human Kinetics. 7(1), 125–134. doi:doi:10.1515/bhk-2015-0019	8. Avila-Quintero et al. 2024<br>9. Crossley et al. 2020

8.	Hammes D, Aus der Fünten K, Kaiser S, et al. Injury prevention in male veteran football players - a randomised controlled trial using "FIFA 11+". J Sports Sci 2015;33:873–81.	10. Lemes et al. 2021
9.	Hasebe Y, Akasaka K, Otsudo T, et al. Effects of Nordic hamstring exercise on hamstring injuries in high school soccer players: a randomized controlled trial. Int J Sports Med 2020;41:154–60.	11. Lemes et al. 2021
10.	Heidt RS, Sweeterman LM, Carlonas RL, et al. Avoidance of soccer injuries with preseason conditioning. Am J Sports Med 2000;28:659–62.	12. Crossley et al. 2020
11.	Owoeye OBA, Akinbo SRA, Tella BA, et al. Efficacy of the FIFA 11+ warm-up programme in male youth football: a cluster randomised controlled trial. J Sports Sci Med 2014;13:321–8.	13. Lemes et al. 2021
12.	Petersen, J., Thorborg, K., Nielsen, M., Budtz-Jørgensen, E., & Hölmich, P. (2011). Preventive effect of eccentric training on acute hamstring injuries in men's soccer: a cluster-randomized controlled trial. Am J Sports Med, 39(11), 2296-2303. doi:10.1177/0363546511419277	14. Avila-Quintero et al. 2024
13.	Silvers-Granelli H, Mandelbaum B, Adeniji O, et al. Efficacy of the FIFA 11+ injury prevention program in the collegiate male soccer player. Am J Sports Med. 2015;43(11):2628-2637.	15. Al Attar et al. 2023 B<br>16. Thorborg et al. 2017
14.	Söderman K, Werner S, Pietilä T, et al. Balance board training: prevention of traumatic injuries of the lower extremities in female soccer players? A prospective randomized intervention study. Knee Surg Sports Traumatol Arthrosc 2000;8:356–63.	17. Crossley et al. 2020

15.	Soligard T, Myklebust G, Steffen K, et al. Comprehensive warm-up programme to prevent injuries in young female footballers: cluster randomised controlled trial. BMJ. 2008;337:a2469	18. Al Attar et al. 2023 B<br>19. Avila-Quintero et al. 2024<br>20. Crossley et al. 2020<br>21. Lemes et al. 2021<br>22. Obërtinca et al. 2023<br>23. Thorborg et al. 2017
16.	van de Hoef PA, Brink MS, Huisstede BMA, et al. Does a bounding exercise program prevent hamstring injuries in adult male soccer players? - A cluster-RCT. Scand J Med Sci Sports 2019;29:515–23.	24. Lemes et al. 2021<br>25. Obërtinca et al. 2023
17.	van der Horst N, Hoef Svande, Otterloo Pvan, et al. Effective but not adhered to: how can we improve adherence to evidence-based hamstring injury prevention in amateur football? Clin J Sport Med 2021;31:42–8.	26. Lemes et al. 2021
18.	van der Horst N, Smits DW, Petersen J, Goedhart EA, Backx FJG. The preventive effect of the Nordic hamstring exercise on hamstring injuries in amateur soccer players: a randomized controlled trial. Am J Sports Med. 2015;43(6):1316-1323.	27. Al Attar et al. 2023 B<br>28. Avila-Quintero et al. 2024
19.	Whalan, M., Lovell, R., Steele, J. R., & Sampson, J. A. (2019). Rescheduling Part 2 of the 11+ reduces injury burden and increases compliance in semi-professional football. Scand J Med Sci Sports, 29(12), 1941-1951. doi:10.1111/sms.13532	29. Avila-Quintero et al. 2024
