# Supplementary material for: Protecting Athletes: The Clinical Relevance of Meta-Analyses on Injury Prevention Programs for Sports and Musculoskeletal Body Regions: An Overview of Systematic Reviews with Meta-Analyses of Randomized Clinical Trials
Source: Healthcare (Basel). 2025 Jun 27;13(13):1530. doi: 10.3390/healthcare13131530 (PMC12250077; doi:10.3390/healthcare13131530)
Supplement: Supplementary file 1 [file healthcare-13-01530-s001.zip › Suppl File S8 Overlap groin and hip injuries.pdf]

Supplementary file S8. Matrices of evidence and the corrected covered area (CCA) calculations for meta-analyses evaluating hip/groin injuries.

Note: The following reviews were not included in the overlap calculation because they did not specify the studies that were included in the meta-analysis.

$$CCA = \frac{N-r}{rc-r} = \frac{10-6}{18-6} \quad \frac{4}{12} = 0.3333 = 33\%$$

Note: N is the total number of original studies (including duplicates) in the meta-analyses of interest (the sum of all checked boxes in the citation matrix). Furthermore, r is the number of original studies without accounting for duplicates. Finally, c is the number of systematic reviews included in the evidence matrix (k=3). CCA = corrected covered area.

**Number of studies without accounting for duplicates**	**Primary research (references)**	**Systematic reviews where primary research appear including primary research duplicates**
1.	Heidt RS, Sweeterman LM, Carlonas RL, et al. Avoidance of soccer injuries with preseason conditioning. Am J Sports Med 2000;28:659–62.	1. Crossley et al. 2020
2.	Rössler R, Junge A, Bizzini M, et al. A multinational cluster randomised controlled trial to assess the efficacy of '11+ Kids': a warm-up programme to prevent injuries in children's football. Sports Med 2018;48:1493–504.	2. Crossley et al. 2020<br>3. Obërtinca et al. 2023

3.	Silvers-Granelli H, Mandelbaum B, Adeniji O, et al. Efficacy of the FIFA 11+ injury prevention program in the collegiate male soccer player. Am J Sports Med 2015;43:2628–37.	4. Thorborg et al. 2017
4.	Söderman K, Werner S, Pietilä T, et al. Balance board training: prevention of traumatic injuries of the lower extremities in female soccer players? A prospective randomized intervention study. Knee Surg Sports Traumatol Arthrosc 2000;8:356–63.	5. Crossley et al. 2020
5.	Soligard T, Myklebust G, Steffen K, et al. Comprehensive warm-up programme to prevent injuries in young female footballers: cluster randomised controlled trial. BMJ 2008;337:a2469.	6. Crossley et al. 2020<br>7. Obërtinca et al. 2023<br>8. Thorborg et al. 2017
6.	Steffen K, Myklebust G, Olsen OE, et al. Preventing injuries in female youth football - a cluster-randomized controlled trial. Scand J Med Sci Sports 2008;18:605–14.	9. Crossley et al. 2020<br>10. Obërtinca et al. 2023
