# Supplementary material for: Protecting Athletes: The Clinical Relevance of Meta-Analyses on Injury Prevention Programs for Sports and Musculoskeletal Body Regions: An Overview of Systematic Reviews with Meta-Analyses of Randomized Clinical Trials
Source: Healthcare (Basel). 2025 Jun 27;13(13):1530. doi: 10.3390/healthcare13131530 (PMC12250077; doi:10.3390/healthcare13131530)
Supplement: Supplementary file 1 [file healthcare-13-01530-s001.zip › Suppl File S9 Overlap ankle injuries.pdf]

Supplementary file S9. Matrices of evidence and the corrected covered area (CCA) calculations for meta-analyses evaluating ankle injuries.

Note: The following reviews were not included in the overlap calculation because they did not specify the studies that were included in the meta-analysis.

$$CCA = \frac{N-r}{rc-r} = \frac{39-21}{126-21} = \frac{18}{105} = 0.1714 = 17\%$$

Note: N is the total number of original studies (including duplicates) in the meta-analyses of interest (the sum of all checked boxes in the citation matrix). Furthermore, r is the number of original studies without accounting for duplicates. Finally, c is the number of systematic reviews included in the evidence matrix (k=6). CCA = corrected covered area.

**Number of studies without accounting for duplicates**	**Primary research (references)**	**Systematic reviews where primary research appear including primary research duplicates**
1.	Al Attar WSA, Soomro N, Pappas E, Sinclair PJ, Sanders RH. Adding a post-training FIFA 111 exercise program to the pre-training FIFA 111 injury prevention program reduces injury rates among male amateur soccer players: a cluster-randomised trial. J Physiother. 2017;63:235–242.	1. Al Attar et al. 2022 B

2.	Al Attar, W.S.A.; Bizzini, M.; Alzahrani, H.; Alarifi, S.; Ghulam, H.; Alyami, M.; Alzhrani, M.; Sanders, R.H. The FIFA11+ Kids Injury Prevention Program Reduces Injury Rates Among Male Children Soccer Players: A Clustered Randomized Controlled Trial. Sports Health 2022, 19417381221109224	2. Yang et al. 2022
3.	Ekstrand J, Gillquist J, Liljedahl SO. Prevention of soccer injuries. Supervision by doctor and physiotherapist. Am J Sports Med. 1983 May-Jun;11(3):116-20.	3. Grimm et al. 2016
4.	Emery C, Meeuwisse W. The effectiveness of a neuromuscular prevention strategy to reduce injuries in youth soccer: a cluster-randomised controlled trial. Br J Sports Med. 2010;44:555–562.	4. Al Attar et al. 2022 B<br>5. Crossley et al. 2020<br>6. Grimm et al. 2016<br>7. Obërtinca et al. 2023
5.	Engebretsen AH, Myklebust G, Holme I, Engebretsen L, Bahr R. Prevention of injuries among male soccer players: a prospective, randomized intervention study targeting players with previous injuries or reduced function. Am J Sports Med. 2008;36:1052–1060.	8. Al Attar et al. 2022 B<br>9. Grimm et al. 2016
6.	Foss KDB, Thomas S, Khoury JC, Myer GD, Hewett TE. A school-based neuromuscular training program and sport-related injury incidence: a prospective randomized controlled clinical trial. J Athl Train. 2018;53:20–28.	10. Al Attar et al. 2022 B<br>11. Crossley et al. 2020
7.	Heidt RS, Sweeterman LM, Carlonas RL, et al. Avoidance of soccer injuries with preseason conditioning. Am J Sports Med 2000;28:659–62.	12. Crossley et al. 2020<br>13. Grimm et al. 2016
8.	LaBella CR, Huxford MR, Grissom J, et al. Effect of neuromuscular warm-up on injuries in female soccer and Basketball athletes in urban public high schools. Arch Pediatr Adolesc Med 2011;165:1033–40.	14. Crossley et al. 2020
9.	Ma, Y. Application Research of FIFA11+ Kids in Primary School Physical Education Class; RCT, Tianjin Normal University: Tianjin, China, 2019.	15. Yang et al. 2022

10.	McGuine TA, Keene JS. The effect of a balance training program on the risk of ankle sprains in high school athletes. Am J Sports Med. 2006;34:1103–1111.	16. Al Attar et al. 2022 B
11.	Mohammadi F. Comparison of 3 preventive methods to reduce the recurrence of ankle inversion sprains in male soccer players. Am J Sports Med. 2007 Jun;35 (6):922-6. Epub 2007 Mar 22.	17. Grimm et al. 2016
12.	Nuhu A, Jelsma J, Dunleavy K, Burgess T. Efect of the FIFA 11 + soccer specifc warm up programme on the incidence of injuries: a cluster-randomised controlled trial. PLoS ONE. 2021;16(5): e0251839. https://doi.org/10.1371/journal.pone.0251839.	18. Obërtinca et al. 2023
13.	Owoeye OB, Akinbo SR, Tella BA, Olawale OA. Efficacy of the FIFA 111 warm-up programme in male youth football: a cluster randomised controlled trial. J Sports Sci Med. 2014;13:321–328.	19. Al Attar et al. 2022 B<br>20. Obërtinca et al. 2023<br>21. Thorborg et al. 2017
14.	Rössler R, Junge A, Bizzini M, Verhagen E, Chomiak J, Meyer T, et al. A multinational cluster randomised controlled trial to assess the efficacy of '111 Kids': a warm-up programme to prevent injuries in children's football. Sports Med. 2018;48:1493–1504.	22. Al Attar et al. 2022 B<br>23. Crossley et al. 2020<br>24. Obërtinca et al. 2023<br>25. Yang et al. 2022
15.	Silvers-Granelli H, Mandelbaum B, Adeniji O, Insler S, Bizzini M, Pohlig R, et al. Efficacy of the FIFA 111 injury prevention program in the collegiate male soccer player. Am J Sports Med. 2015;43:2628–2637.	26. Al Attar et al. 2022 B<br>27. Thorborg et al. 2017
16.	Soderman K, Werner S, Pietil¨a T, Engstr¨om B, Alfredson H. Balance board training: prevention of traumatic injuries of the lower extremities in female soccer players? A prospective randomized intervention study. Knee Surg Sports Traumatol Arthrosc. 2000;8(6):356-63.	28. Grimm et al. 2016

17.	Soligard T, Myklebust G, Steffen K, Holme I, Silvers H, Bizzini M, et al. Comprehensive warm-up programme to prevent injuries in young female footballers: cluster randomised controlled trial. BMJ. 2008;337:a2469.	29. Al Attar et al. 2022 B<br>30. Crossley et al. 2020<br>31. Grimm et al. 2016<br>32. Obërtinca et al. 2023<br>33. Thorborg et al. 2017
18.	Steffen K, Myklebust G, Olsen OE, et al. Preventing injuries in female youth football - a cluster-randomized controlled trial. Scand J Med Sci Sports 2008;18:605–14.	34. Crossley et al. 2020<br>35. Obërtinca et al. 2023
19.	Tropp H, Askling C, Gillquist J. Prevention of ankle sprains. Am J Sports Med. 1985 Jul-Aug;13(4):259-62.	36. Grimm et al. 2016
20.	van Beijsterveldt AM, van de Port IG, Krist MR, Schmikli SL, Stubbe JH, Frederiks JE, Backx FJ. Effectiveness of an injury prevention programme for adult male amateur soccer players: a cluster-randomised controlled trial. Br J Sports Med. 2012 Dec;46(16):1114-8. Epub 2012 Aug 9.	37. Grimm et al. 2016
21.	Zarei M, Abbasi H, Namazi P, Asgari M, Rommers N, Rössler R. The 11+ Kids warm-up programme to prevent injuries in young Iranian male high-level football (soccer) players: a cluster-randomised controlled trial. J Sci Med Sport. 2020;23(5):469–74. https://doi.org/10.1016/j.jsams.2019.12.001.	38. Obërtinca et al. 2023<br>39. Yang et al. 2022
